# Preparation of conjugated dienoates with Bestmann ylide: Towards the synthesis of zampanolide and dactylolide using a facile linchpin approach

**DOI:** 10.3762/bjoc.11.197

**Published:** 2015-10-05

**Authors:** Jingjing Wang, Samuel Z Y Ting, Joanne E Harvey

**Affiliations:** 1 Centre for Biodiscovery, School of Chemical and Physical Sciences, Victoria University of Wellington, PO Box 600, Wellington 6140, New Zealand

**Keywords:** Bestmann ylide, dactylolide, dienoate, (triphenylphosphoranylidene)ketene, zampanolide

## Abstract

Bestmann ylide [(triphenylphosphoranylidene)ketene] acts as a chemical linchpin that links nucleophilic entities, such as alcohols or amines, with carbonyl moieties to produce unsaturated esters and amides, respectively. In this work, the formation of α,β,γ,δ-unsaturated esters (dienoates) is achieved through the coupling of Bestmann ylide, an alcohol and an α,β-unsaturated aldehyde. Primary and secondary alcohols, including allylic alcohols, are suitable substrates; the newly formed alkene has an *E*-geometry. Strategically, this represents a highly efficient route to unsaturated polyketide derivatives. A linchpin approach to the synthesis of a major fragment of the natural products zampanolide and dactylolide is investigated using Bestmann ylide to link the C16–C20 alcohol with the C3–C8 aldehyde fragment.

## Introduction

(Triphenylphosphoranylidene)ketene, Ph_3_P=C=C=O (**1**), was first reported in 1966 [1–2]. It initially attracted attention due to its unique structure, namely the 145.5° angle of the C=C=P moiety and the unusually short C=C bond (1.210 Å). Its utility was subsequently explored, with pioneering work by Bestmann and co-workers [3–5] lending the name *Bestmann ylide* to this versatile and readily obtained reagent [6–8]. Studies revealed that the ylide readily reacts with alcohols and amines to form α-phosphoranylidene esters or amides, providing diverse isolable Wittig reagents that can be used in subsequent transformations [4–5,9–11]. Furthermore, if the α-phosphoranylidene ester or amide is formed in the presence of an aldehyde, ketone or ester, an additional in situ Wittig reaction can occur [12–18]. In this way, amides, esters and thioesters can be obtained through three-component couplings [14,16]. Intramolecular couplings with a Bestmann ylide linchpin have enabled direct lactone and lactam synthesis [12–15], including the preparation of macrolactones [16–18]. An extension of this methodology to γ-hydroxyenone substrates allows the preparation of α-alkylidene-γ-butyrolactones through tandem acylation and Michael addition, followed by a Wittig reaction [19–20].

**Figure 1:**
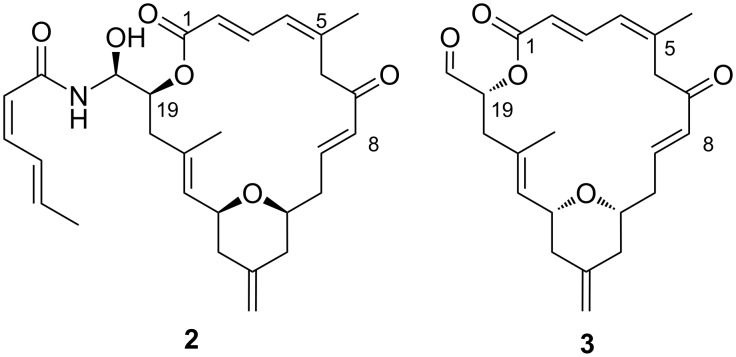
Structures of (−)-zampanolide (**2**) and (+)-dactylolide (**3**).

Although the utility of the Bestmann ylide in the synthesis of acyclic α,β-unsaturated esters and dienamides has already been reported [11,13,16], its application to the synthesis of α,β,γ,δ-unsaturated esters (i.e., dienoates) remains uncharted. In this paper, a three-component reaction between α,β-unsaturated aldehydes, alcohols and the Bestmann ylide is described. The scope of this esterification–Wittig reaction sequence in the synthesis of α,β,γ,δ-unsaturated esters is studied, and the method is applied in an approach towards the structurally related marine natural products, zampanolide (**2**) and dactylolide (**3**, Figure 1), wherein the Bestmann ylide represents a C1–C2 linchpin that connects two segments of the macrocylic ring.

(−)-Zampanolide (**2**, Figure 1) was first isolated from the marine sponge *Fasciospongia rimosa* found at Cape Zampa, Japan [21], and subsequently from a Tongan sponge *Cacospongia mycofijiensis* [22]. It was found to exhibit potent anticancer activities, with IC_50_ values in the nM scale against a number of cell lines [22–23]. The structurally related compound, (+)-dactylolide, was discovered in the marine sponge *Dactylospongia* [24] and has significantly lower cytotoxicity. The absolute configuration of the natural material is not firmly established due to discrepancies in optical rotation values between natural and synthetic samples [25].

**Scheme 1:**
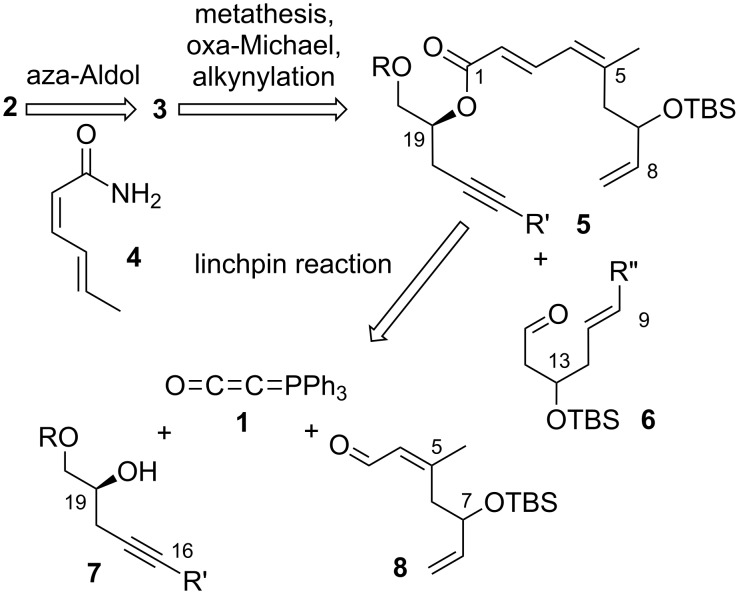
Retrosynthesis of zampanolide involving a Bestmann ylide linchpin strategy.

Zampanolide and dactylolide have engendered world-wide interest from the synthetic community, culminating in a number of total syntheses [26–41]. Zampanolide is invariably prepared by appending the amide side-chain **4** to the aldehyde moiety of dactylolide, so the synthesis of zampanolide requires the prior generation of dactylolide. Although fragment syntheses vary, the late-stage fragment assembly of the dactylolide macrocycle has centred mostly around construction of the C1–C5 dienoate by Wittig-type olefination reactions followed by ester hydrolysis and esterification with the C19 hydroxy group, combined with metathesis to form the alkene at C8–C9. In our synthesis, the doubly unsaturated ester moiety will be formed through an efficient linchpin reaction between the C16–C20 fragment (alcohol **7**) [42–45], the C3–C8 fragment (α,β-unsaturated aldehyde **8**) and Bestmann ylide (**1**, Scheme 1). We plan to then attach the C9–C15 aldehyde fragment **6** by asymmetric alkynylation, and form the pyran using an oxa-Michael addition, in a manner reminiscent of that employed by Uenishi and co-workers [34]. Finally, macrocyclisation will be achieved through the well-established strategy of ring-closing metathesis at C8–C9.

## Results and Discussion

### Evaluation of the three-component reaction for the synthesis of α,β,γ,δ-unsaturated esters

The study of the reactions between Bestmann ylide, alcohols and α,β-unsaturated aldehydes began with the investigation of the coupling between linchpin **1** [46], *E*-hex-2-en-1-ol (**9a**) and *E*-cinnamaldehyde (**10a**) (Table 1, entry 1). Typically, Bestmann ylide reactions are performed at elevated temperature in a high boiling and/or ether solvent, such as toluene, 1,4-dioxane or THF [11,14–17,19–20]. To investigate the necessity for high temperature, the primary reaction in this study was initiated at room temperature (19 °C) in toluene and then progressively warmed to reflux (110 °C) while monitoring the progress by TLC. It was noted that the Bestmann ylide reagent was insoluble up to 80 °C and no reaction was observed until the reaction mixture was heated at reflux. Under these conditions, incomplete consumption of the starting materials was seen after 18 h and the poor conversion was attributed to the instability of Bestmann ylide over long periods at elevated temperatures and in the presence of any adventitious nucleophilic source. Nonetheless, the product **11a** was obtained in a modest yield (Table 1, entry 1). In response to these observations, the reaction was attempted in THF, a solvent in which Bestmann ylide readily dissolved at room temperature. This provided better conversion, although the isolated yield was only marginally improved (Table 1, entry 2). Gratifyingly, the reaction of oct-2-en-1-ol (**9b**) with cinnamaldehyde (**10a**) was efficient and high yielding (Table 1, entry 3). Use of a *Z*-allylic alcohol **9c**, likewise produced excellent amounts of the product dienoate (Table 1, entry 4), although a longer reaction time was required to achieve this. The *Z*-geometry of the allylic alcohol was retained, as expected. After this, the secondary allylic alcohol **9d** was investigated and a reasonable yield of the product was obtained when the reaction was carried out in THF (Table 1, entry 5). A comparative reaction in toluene was also performed and found to deliver a better yield of the product (Table 1, entry 6). The saturated secondary alcohol menthol (**9e**), with additional steric encumbrance and stereogenic centres, provided a good yield of the product **11e** [47] in THF, despite incomplete conversion (Table 1, entry 7). Full conversion but a poor isolated yield of the product was achieved in toluene after reaction for 23 hours (Table 1, entry 8). Decreasing the reaction time provided better results (Table 1, entry 9), indicating that the product may decompose upon prolonged periods at elevated temperature. The Bestmann ylide coupling of menthol (**9e**) and octa-2,4-dienal (**10b**) delivered the trienoate product **11f** in good yields at both 0.1 and 0.3 mmol scale (Table 1, entries 10 and 11). Taken together, these results indicate that both primary and secondary alcohol substrates react effectively with conjugated unsaturated aldehydes and the Bestmann ylide linchpin in either THF or toluene, although prolonged reflux in toluene may cause degradation of the products. Small quantities (<10%) of isomeric products, presumed to be the corresponding 2*Z*,4*E*-dienes of **11a**, **11b** and **11c**, were observed in the reaction mixtures derived from the primary alcohols. Only barely trace amounts of *Z*-alkenes were ever observed with secondary alcohols. As the minor isomers were not able to be isolated, their identities are unconfirmed.

**Table 1:** Coupling reactions of alcohols **9** and aldehydes **10** with Bestmann ylide (**1**)^a^.

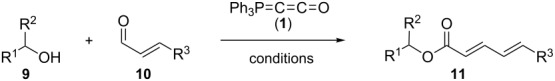					

Entry	Alcohol	Aldehyde	Conditions^b^	Product	Yield^c^

1	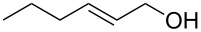 **9a**	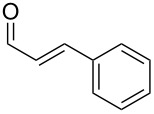 **10a**	toluene, rt to 110 °C, 18 h^d^	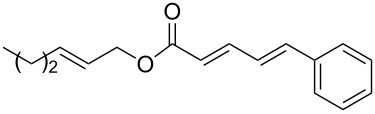 **11a**	53% (60%)

2	**9a**	**10a**^e^	THF, 66 °C, 2 h^f^	**11a**	55% (94%)

3	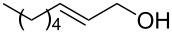 **9b**	**10a**	THF, 66 °C, 1.5 h	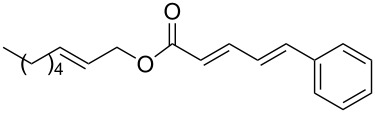 **11b**	93% (100%)

4	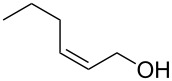 **9c**	**10a**	THF, 66 °C, 6 h^g^	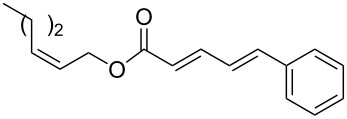 **11c**	91% (100%)

5	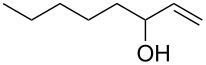 **9d**	**10a**	THF, 66 °C, 4.5 h	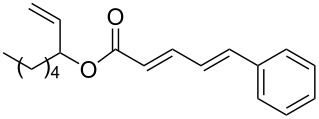 **11d**	61% (100%)

6	**9d**	**10a**	toluene, 110 °C, 5.5 h	**11d**	71% (94%)

7	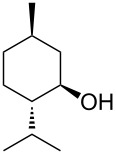 **9e**	**10a**	THF, 66 °C, 22 h	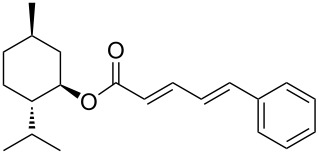 **11e**	77% (85%)

8	**9e**	**10a**	toluene, 110 °C, 23 h	**11e**	29% (100%)

9	**9e**	**10a**	toluene, 110 °C, 9.5 h^g^	**11e**	53% (100%)

10	**9e**	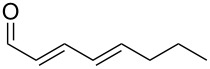 **10b**	toluene, 110 °C, 3 h	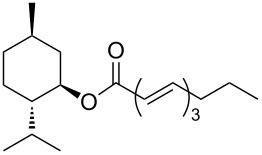 **11f**	67% (90%)

11	**9e**	**10b**	toluene, 110 °C, 4.5 h^g^	**11f**	66% (100%)

12	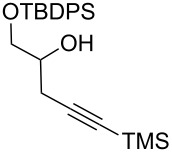 **7a**	**10a**	THF, 66 °C, 3.5 h	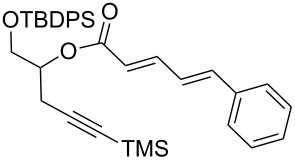 **11g**	<36% (43%)^h^

13	**7a**	**10a**	toluene, 110 °C, 2 h	**11g**	<49% (73%)^h^

14	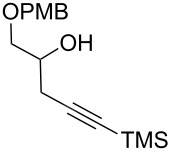 **7b**	**10a**	THF, 66 °C, 8 h	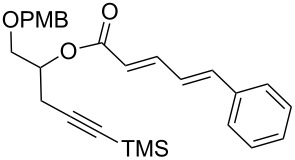 **11h**	42% (69%)

15	**7b**	**10a**	toluene, 110 °C, 4 h^g^	**11h**	70% (100%)

^a^Unless otherwise stated, reactions were performed on a 0.1 mmol scale using approximately 1:1:1 ratio of alcohol/Bestmann ylide/aldehyde. ^b^Solvent, temperature, reaction time. ^c^Isolated yield. Conversion (given in brackets) was calculated based on relative integrations of peaks assigned to the limiting reagent (aldehyde) and product in ^1^H NMR spectra of the crude reaction mixture after work-up. ^d^Reaction was carried out on 0.8 mmol scale. ^e^0.57 equiv of aldehyde **10a** were used. ^f^Reaction was carried out on 0.2 mmol scale. ^g^Reaction was carried out on 0.3 mmol scale. ^h^Product **11g** was contaminated with the regioisomer resulting from silyl migration and esterification of the primary hydroxy group (3:2 ratio **11g**:isomeric C20 ester).

After these promising results, the homopropargylic secondary alcohol **7a** [42], representing the C16–C20 fragment of zampanolide, was subjected to Bestmann ylide linchpin reactions with cinnamaldehyde (**10a**) in THF and toluene (Table 1, entries 12 and 13). In an attempt to avoid degradation, the reactions were terminated prior to full conversion, leading to the dienoate product in modest yields. Unfortunately, it was found that the product was not isolable in pure form but was contaminated with further isomeric material. Careful analysis of the product mixtures led to the realisation that silyl migration from the primary to the secondary hydroxy group was occurring in the reaction, leading to the C20-linked ester isomer in addition to the desired C19 ester **11g**. In contrast, reactions of the PMB-protected variant **7b** [43–45] with cinnamaldehyde afforded the product **11h** in pure form and reasonable-to-good yields in THF and toluene (Table 1, entries 14 and 15, respectively).

### Preparation of the C3–C8 fragment of dactylolide/zampanolide

**Scheme 2:**
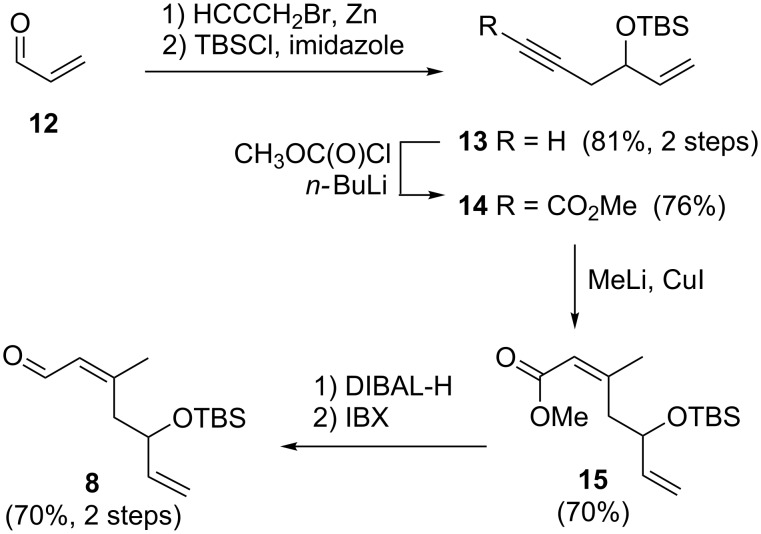
Synthesis of aldehyde **8**.

The aldehyde **8**, representing the C3–C8 fragment of zampanolide, was synthesised from acrolein (**12**) (Scheme 2). Firstly, Barbier reaction of acrolein with propargyl bromide followed by silyl protection of the resulting alcohol afforded enyne **13**. Treatment of the lithium alkynylide derived from **13** with methyl chloroformate produced the methyl ester **14** [48]. Cuprate-mediated conjugate addition of a methyl nucleophile to the ynoate **14** provided the *Z*-enoate **15**. Our attempts to directly reduce the ester **15** to the aldehyde **8** were unsuccessful due to competitive over-reduction to the corresponding alcohol. Therefore, a two-step reduction–oxidation process was undertaken to afford the aldehyde **8** in a good yield.

### Three-component coupling of dactylolide/zampanolide fragments with Bestmann ylide

**Scheme 3:**
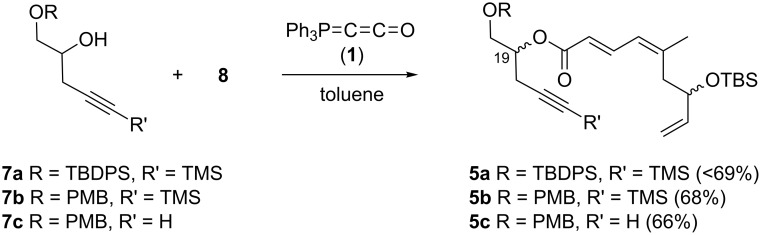
Bestmann ylide linchpin coupling of the C16–C20 and C3–C8 fragments of zampanolide/dactylolide.

With aldehyde **8** in hand, a reaction with Bestmann ylide (**1**) was performed in toluene using the TBDPS-protected alcohol **7a**. The α,β,γ,δ-unsaturated ester **5a** was formed as a mixture of diastereomers (Scheme 3), as expected, but was again contaminated with the regioisomer resulting from silyl migration (2:1 ratio **5a**:isomeric C20 ester). Employing the PMB-protected C19 alcohols **7b** [43–45] and **7c** [43–45,49] led to the desired products **5b** and **5c**, respectively. While the reaction of **7b** with aldehyde **8** in THF took 11 h to go to completion (62% isolated yield of **5b**), the equivalent reaction in toluene required only 5 h (68% yield). Alcohol **7c** was used to assess the compatibility of an unprotected, terminal alkyne in this linchpin reaction with a view to future synthetic ease [50]. Pleasingly, the reaction of aldehyde **8** with alcohol **7c** in toluene provided the desired product **5c** in a comparable yield (66%) after 5 h. In these reactions, only the desired *E*,*Z*-diene isomer was observed.

## Conclusion

In summary, an efficient three-component reaction between (triphenylphosphoranylidene)ketene (Bestmann ylide, **1**), an alcohol and an unsaturated aldehyde delivers α,β,γ,δ-unsaturated esters. This methodology enabled the facile synthesis of *E,Z*-dienoate products **5b** and **5c**, which represent two-thirds of the dactylolide/zampanolide macrocycle, suitably functionalised for transformation to the natural products. This highly efficient method for connecting the C3–C8 and C16–C20 fragments through a C1–C2 linchpin in a single step contrasts with other routes that require multiple-step sequences including Wittig-type reaction, ester hydrolysis and coupling to the C19 alcohol. Elaboration of these compounds into the targets – dactylolide, zampanolide and analogues thereof – is currently under development in our lab.

## Experimental

**General procedure for Bestmann ylide linchpin reaction:** To a mixture of alcohol (1 equiv, 0.1–0.3 M) and Bestmann ylide (1 equiv) in solvent (toluene or tetrahydrofuran) heated at reflux, a solution of aldehyde (1 equiv, 1.0 M) was added. The reaction was heated at reflux until full consumption of starting material aldehyde was observed by TLC. After cooling to rt, the reaction was concentrated and purified by silica column chromatography.

**(2′*E*,2*E*,4*E*)-Hex-2′-enyl 5-phenylpenta-2,4-dienoate (11a).** *R*_f_ = 0.23 (20:1 pet. ether:Et_2_O); ^1^H NMR (500 MHz, CDCl_3_) δ 7.48–7.43 (complex m, 3H), 7.36 (app. t, *J* = 7.6 Hz, 2H), 7.30 (t, *J* = 6.9 Hz, 1H), 6.93–6.82 (complex m, 2H), 6.01 (d, *J* = 15.6 Hz, 1H), 5.81 (dt, *J* = 15.4, 6.6 Hz, 1H), 5.62 (dt, *J* = 15.4, 6.2 Hz, 1H), 4.63 (d, *J* = 6.6 Hz, 2H), 2.14 (app. q, *J* = 7.1 Hz, 2H), 1.43 (app. sext, *J* = 7.4 Hz, 2H), 0.92 (t, *J* = 7.3 Hz, 3H); ^13^C NMR (125 MHz, CDCl_3_) δ 166.8, 144.7, 140.4, 136.3, 136.0, 129.0, 128.8, 127.2, 126.2, 124.0, 121.2, 65.2, 34.3, 22.0, 13.6; IR (neat) cm^−1^: 2958 (m), 2929 (m), 1706 (s), 1625 (s), 1449 (m), 1236 (s), 1172 (s), 997 (m), 689 (m); HRMS (ESI) *m*/*z*: [M + H]^+^ calcd for C_17_H_21_O_2_, 257.1536 found, 257.1529, (Δ = 2.7 ppm).

**(1′*R*,2′*S*,5′*R*,2*E*,4*E*,6*E*)-2′-Isopropyl-5′-methylcyclohex-1′-yl deca-2,4,6-trienoate (11f).** *R*_f_ = 0.14 (40:1 pet. ether:Et_2_O); ^1^H NMR (500 MHz, CDCl_3_) δ 7.28 (dd, *J* = 15.4, 11.2 Hz, 1H), 6.53 (dd, *J* = 14.9, 10.7 Hz, 1H), 6.21 (dd, *J* = 14.9, 11.2 Hz, 1H), 6.13 (dd, *J* = 15.1, 10.7 Hz, 1H), 5.92 (dt, *J* = 14.9, 7.2 Hz, 1H), 5.83 (d, *J* = 15.4 Hz, 1H), 4.75 (app. td, *J* = 10.9, 4.4 Hz, 1H), 2.12 (app. q, *J* = 7.3 Hz, 2H), 2.02 (br d, *J* = 12.2 Hz, 1H), 1.88 (septd, *J* = 6.8, 2.3 Hz, 1H), 1.71–1.65 (complex m, 2H), 1.50 (partially obs. m, 1H), 1.44 (m, 2H), 1.40 (partially obs. m, 1H), 1.07 (app. qd, *J* = 12.9, 2.9 Hz, 1H), 0.99 (app. q, *J* = 11.5 Hz, 1H), 0.91 (t, *J* = 7.3 Hz, 3H), 0.90 (d, *J* = 6.1 Hz, 3H), 0.89 (d, *J* = 6.8 Hz, 3H), 0.87 (partially obs. m, 1H), 0.76 (d, *J* = 7.1 Hz, 3H); ^13^C NMR (125 MHz, CDCl_3_) δ 166.8, 144.6, 141.0, 140.2, 130.0, 127.8, 120.5, 73.9, 47.2, 41.0, 35.0, 34.3, 31.4, 26.3, 23.6, 22.2, 22.0, 20.7, 16.4, 13.7; IR (neat) cm^−1^: 2955 (s), 2930 (s), 2869 (s), 1694 (s), 1615 (s), 1456 (m), 1342 (m), 1133 (s), 1007 (s); HRMS (ESI) *m*/*z*: [M + H]^+^ calcd for C_20_H_33_O_2_, 305.2475; found, 305.2486, (Δ = 3.6 ppm). [α]_D_^22^ = −26 (*c* 0.42, CH_2_Cl_2_).

**(2*E*,4*E*)-[1′-(*para*-Methoxybenzyloxy)-5′-trimethylsilyl]pent-4′-yn-2′-yl 5-phenylpenta-2,4-dienoate (11h). ***R*_f_ = 0.28 (80% CH_2_Cl_2_ in *n*-hexane); ^1^H NMR (500 MHz, CDCl_3_) δ 7.51–7.44 (complex m, 3H), 7.37 (app. t, *J* = 7.1 Hz, 2H), 7.33 (m, 1H), 7.28 (br d, *J* = 8.8 Hz, 2H), 6.95–6.84 (complex m, 4H), 6.02 (d, *J* = 15.4 Hz, 1H), 5.20 (app. dt, *J* = 10.5, 5.7 Hz, 1H), 4.55 (d, *J* = 11.5 Hz, 1H), 4.50 (d, *J* = 11.7 Hz, 1H), 3.80 (s, 3H), 3.72–3.67 (m, 2H), 2.68 (dd, *J* = 17.1, 7.1 Hz, 1H), 2.63 (dd, *J* = 16.9, 5.9 Hz, 1H), 0.14 (s, 9H); ^13^C NMR (125 MHz, CDCl_3_) δ 166.2, 159.2, 145.2, 140.7, 136.0, 130.1, 129.3, 129.1, 128.8, 127.2, 126.2, 121.0, 113.8, 101.9, 87.1, 73.0, 70.7, 69.4, 55.2, 22.3, 0.00; IR (neat) cm^−1^: 3028 (w), 2957 (m), 2901 (m), 2178 (m), 1709 (s), 1625 (s), 1512 (m), 1245 (s), 1128 (s), 840 (s), 757 (s); HRMS (ESI) *m*/*z*: [M + H]^+^ calcd for C_27_H_33_O_4_Si, 449.2143; found, 449.2146, (Δ = 0.67 ppm).

**(2*E*,4*Z*)-1′-(*para*-Methoxybenzyloxy)-5′-(trimethylsilyloxy)pent-4′-yn-2′-yl 7-(*tert*-butyldimethylsilyloxy)-5-methylnona-2,4,8-trienoate (5b).** *R*_f_ = 0.30 (80% CH_2_Cl_2_ in *n*-hexane); ^1^H NMR (500 MHz, CDCl_3_) δ 7.57 (dd, *J* = 14.9, 11.8 Hz, 1H), 7.26 (d, *J* = 8.3 Hz, 2H), 6.87 (d, *J* = 8.3 Hz, 2H), 6.07 (d, *J* = 11.5 Hz, 1H), 5.80 (obs. ddd, *J* = 16.8, 10.6, 5.7 Hz, 1H), 5.80 (obs. d, *J* = 15.2 Hz, 1H), 5.23–5.11 (obs. m, 1H), 5.18 (obs. d, *J* = 17.2 Hz, 1H), 5.05 (d, *J* = 10.3 Hz, 1H), 4.53 (d, *J* = 11.5 Hz, 1H), 4.48 (d, *J* = 11.5 Hz, 1H), 4.25 (app. q, *J* = 6.2 Hz, 1H), 3.80 (s, 3H), 3.67 (app. d, *J* = 4.9 Hz, 2H), 2.69–2.54 (complex m, 3H), 2.36 (ddd, *J* = 13.0, 6.5, 5.5 Hz, 1H), 1.92 (s, 3H), 0.87 (s, 9H), 0.13 (s, 9H), 0.01 (s, 6H); ^13^C NMR (500 MHz, CDCl_3_) δ 166.71, 166.69, 159.2, 146.58, 146.56, 141.70, 141.67, 140.9, 130.1, 129.3, 126.09, 129.07, 118.89, 118.87, 114.2, 113.8, 102.0, 86.92, 86.90, 73.00, 72.99, 72.86, 72.82, 70.48, 70.47, 69.45, 69.41, 55.2, 41.7, 25.83, 25.82, 25.64, 25.62, 22.32, 22.29, 18.1, 0.00, −0.01, −4.54, −4.88; IR (neat) cm^−1^: 2956 (s), 2857 (m), 2180 (w), 1714 (s), 1636 (m), 1513 (m), 1249 (s), 1033 (m), 837 (s), 775 (m), 760 (m); HRMS (ESI) *m*/*z*: [M + H]^+^ calcd for C_32_H_51_O_5_Si_2_, 571.3270; found, 571.3276, (Δ = 1.1 ppm).

**(2*E*,4*Z*)-1′-(*para*-Methoxybenzyloxy)pent-4′-yn-2′-yl 7-(*tert*-butyldimethylsilyloxy)-5-methylnona-2,4,8-trienoate (5c). ***R*_f_ = 0.32 (80% CH_2_Cl_2_ in *n*-hexane); ^1^H NMR (500 MHz, CDCl_3_) δ 7.58 (dd, *J* = 14.9, 11.7 Hz, 1H), 7.26 (d, *J* = 8.6 Hz, 2H), 6.87 (d, *J* = 8.6 Hz, 2H), 6.07 (d, *J* = 11.5 Hz, 1H), 5.80 (obs. ddd, *J* = 17.1, 10.5, 5.9 Hz, 1H), 5.80 (obs. d, *J* = 14.9 Hz, 1H), 5.23–5.14 (obs. m, 1H), 5.18 (obs. d, *J* = 16.9 Hz, 1H), 5.05 (d, *J* = 10.3 Hz, 1H), 4.53 (d, *J* = 11.5 Hz, 1H), 4.48 (d, *J* = 11.5 Hz, 1H), 4.25 (app. q, *J* = 5.6 Hz, 1H), 3.80 (s, 3H), 3.71–3.62 (m, 2H), 2.65 (ddd, *J* = 16.9, 6.6, 2.7 Hz, 1H), 2.58 (ddd, *J* = 16.9, 5.4, 2.5 Hz, 1H), 2.61–2.54 (obs. m, 1H), 2.36 (app. dt, *J* = 13.1, 4.9 Hz, 1H), 1.97 (m, 1H), 1.92 (s, 3H), 0.86 (s, 9H), 0.01 (s, 3H), 0.00 (s, 3H); ^13^C NMR (500 MHz, CDCl_3_) δ 166.7, 159.2, 146.81, 146.78, 141.92, 141.89, 140.95, 140.93, 130.1, 129.31, 129.30, 126.07, 126.05, 118.71, 118.68, 114.2, 113.8, 79.6, 73.0, 72.81, 72.77, 70.33, 70.30, 70.18, 70.13, 69.3, 55.2, 41.8, 25.8, 25.66, 25.65, 20.97, 20.95, 18.1, −4.54, −4.88; IR (neat) cm^−1^: 3308 (w), 2930 (m), 2857 (s), 2214 (w), 1712 (s), 1635 (m), 1612 (m), 1514 (s), 1248 (s), 1033 (m), 836 (s), 776 (s); HRMS (ESI) *m*/*z*: [M + H]^+^ calcd for C_29_H_43_O_5_Si, 499.2874; found, 499.2868, (Δ = 1.2 ppm).

## Supporting Information

File 1 Full experimental methods; spectroscopic data and NMR spectra of all new compounds.
