# Supplementary material for: Preparation of conjugated dienoates with Bestmann ylide: Towards the synthesis of zampanolide and dactylolide using a facile linchpin approach
Source: Beilstein J Org Chem. 2015 Oct 5;11:1815–22. doi: 10.3762/bjoc.11.197 (PMC4660958; doi:10.3762/bjoc.11.197)
Supplement: File 1 — Full experimental methods; spectroscopic data and NMR spectra of all new compounds. [file Beilstein_J_Org_Chem-11-1815-s001.pdf]

Supporting Information

for

Preparation of conjugated dienoates with Bestmann ylide: Towards the synthesis of zampanolide and dactylolide using a facile linchpin approach

Jingjing Wang[1], Samuel Z. Y. Ting[1] and Joanne E. Harvey*[1]

Address: [1]Centre for Biodiscovery, School of Chemical and Physical Sciences, Victoria University of Wellington, PO Box 600, Wellington 6140, New Zealand

Email: Dr Joanne Harvey - joanne.harvey@vuw.ac.nz

* Corresponding author

Full experimental methods; spectroscopic data and NMR spectra of all new compounds

General experimental information

All reactions were carried out in oven-dried glassware under a positive pressure of nitrogen or argon. Dry tetrahydrofuran, dichloromethane and toluene were obtained from a PureSolv MD 5 solvent purification system (Innovative Technology). Dry dimethylformamide was purchased from Acros and used without further purification. Dry DMSO, triethylamine and oxalyl chloride were purified by distillation with calcium hydride as drying agent. Analytical grade solvents were used for aqueous workup and column chromatography (petroleum ether, ethyl acetate, diethyl ether, dichloromethane and methanol). Column chromatography was performed on silica gel 60Å (Pure Science, 40–63 micron) with the eluent mixtures as stated in the corresponding procedures. Thin-layer chromatography was performed on silica-coated plastic plates (Macherey-Nagel, POLYGRAM® Sil G/UV_{254}). UV-active compounds were detected under UV irradiation (λ = 254 nm), while non-UV-active compounds were visualised with anisaldehyde or potassium permanganate staining solutions.

PMBCl was prepared freshly using the previously reported method.[1] Zinc powder[2] and copper(I) iodide[3] were purified prior to reactions. All other chemicals were purchased from Pure Science, Sigma-Aldrich, Acros, Merck, British Drug House, Burkes Research, Thermo Fisher Scientific, Avocado and Apollo. Infra-red (IR) spectra were collected from liquid films or in neat form on a Perkin Elmer Spectrum One FT-IR spectrometer or an ALPHA FT-IR spectrometer (Bruker) fitted with attenuated total reflectance (ATR). Mass spectra were collected on an Agilent 6530 Accurate-Mass Q-TOF LC/MS high-resolution mass spectrometer (HRMS).

Nuclear magnetic resonance (NMR) spectra were obtained in deuterated chloroform using Varian Inova instruments operating at 300, 500 or 600 MHz for proton and 75, 125 or 150 MHz for carbon. Proton and carbon chemical shifts are reported in parts per million (ppm) relative to residual $CHCl_3$ [$\delta(^1H)$ = 7.26 ppm] and $CDCl_3$ [$\delta(^{13}C)$ = 77.0 ppm], respectively. Signals are defined as: s = singlet, d = doublet, t = triplet, q = quartet, quin = quintet, sext = sextet, sept = septet, m = multiplet, app. = apparent, obs. = obscured peak, br = broad. Coupling constants (*J*) are reported in Hertz (Hz). Assignments were determined by two-dimensional NMR experiments (COSY, NOESY, HSQC, HMBC and CIGAR-HMBC [CIGAR-HMBC = constant time inverse-detection gradient accordion rescaled long-range heteronuclear multiple bond correlation]).

Synthesis of 1-(*tert*-butyldiphenylsilyloxy)-5-trimethylsilyl-4-pentyn-2-ol (7a)

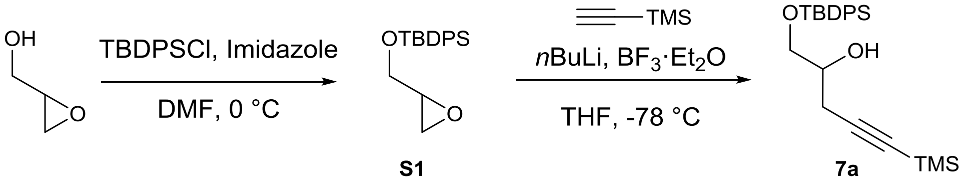

2-(*tert*-Butyldiphenylsilyloxy)methyloxirane (S1)

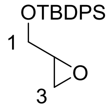

To a solution of glycidol (116 mg, 1.56 mmol) and imidazole (138 mg, 2.03 mmol) in DMF (2.2 mL) at 0 °C, TBDPSCl (0.47 mL, 1.8 mmol) was added dropwise. After stirring at rt for 3 h 30 min, the reaction mixture was quenched with H_2O (10 mL) and diluted with Et_2O (15 mL). The organic layer was separated and washed sequentially with saturated aqueous $NaHCO_3$ solution (2 × 15 mL), H_2O (15 mL), brine (15 mL), and then dried over $MgSO_4$, filtered and concentrated under reduced pressure. This crude product was purified by column chromatography (silica, 50:1 pet. ether/EtOAc, R_f = 0.18) to yield the title compound as a colourless oil (396 mg, 84%).

^{1}H NMR (500 MHz, $CDCl_3$): δ 7.71–7.67 (complex m, 4H, CH, Ph), 7.46–7.37 (complex m, 6H, CH, Ph), 3.85 (dd, J = 11.7, 3.2 Hz, 1H, one of 1-CH_2), 3.71 (dd, J = 11.8, 4.8 Hz, 1H, one of 1-CH_2), 3.14 (m, 1H, 2-CH), 2.75 (t, J = 4.6 Hz, 1H, one of 3-CH_2), 2.62 (dd, J = 5.1, 2.7 Hz, 1H, one of 3-CH_2), 1.06 (s, 9H, CH_3, *t*Bu).

^{13}C NMR (125 MHz, $CDCl_3$): δ = 135.6 (CH, Ph), 135.6 (CH, Ph), 133.3 (C, Ph), 129.7 (CH, Ph), 127.7 (CH, Ph), 64.3 (CH_2, C1), 52.3 (CH, C2), 44.5 (CH_2, C3), 26.7 (CH_3, *t*Bu), 19.2 (C, *t*Bu).

These data matched those reported previously.[1]

1-(*tert*-Butyldiphenylsilyloxy)-5-trimethylsilyl-4-pentyn-2-ol (7a)

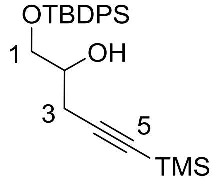

To a solution of ethynyltrimethylsilane (375 mg, 3.82 mmol) in THF (12.7 mL) at -78 °C, was added dropwise a solution of *n*-BuLi (2.0 mL, 1.9 M in cyclohexane, 3.8 mmol). The reaction mixture was stirred at −78 °C for 50 min, before $BF_3{\cdot}Et_2O$ (0.48 mL, 3.81 mmol) was added dropwise. After 10 min stirring, a solution of epoxide **S1** (991 mg, 3.17 mmol) in THF (4.0 mL) was added dropwise. The reaction was stirred at −78 °C for 2 h 40 min, and then quenched with saturated aqueous NH_4Cl (15 mL), and extracted with Et_2O (3 × 40 mL). The organic layers were combined, dried over $MgSO_4$, filtered and concentrated under reduced pressure. This crude product was purified by column chromatography (silica, 20:1 pet. ether/EtOAc, R_f = 0.23) to yield a pale yellow oil (1.18 g, 91%).

^{1}H NMR (500 MHz, $CDCl_3$): δ 7.71–7.67 (complex m, 4H, CH, Ph), 7.44 (t, *J* = 7.2 Hz, 2H, CH, Ph), 7.41 (t, *J* = 7.2 Hz, 4H, CH, Ph), 3.88 (m, 1H, 2-CH), 3.76 (dd, *J* = 10.1, 4.3 Hz, 1H, one of 1-CH_2), 3.69 (dd, *J* = 10.0, 5.9 Hz, 1H, one of 1-CH_2), 2.54–2.48 (m, 2H, 3-CH_2), 1.07 (s, 9H, CH_3, *t*Bu), 0.11 (s, 9H, CH_3, TMS).

^{13}C NMR (125 MHz, $CDCl_3$): δ 135.5 (CH, Ph), 133.3 (C, Ph), 129.8 (CH, Ph), 127.8 (CH, Ph), 102.6 (C, C4), 87.1 (C, C5), 70.2 (CH_2, C1), 66.4 (CH, C2), 26.8 (CH_3, *t*Bu), 24.7 (CH_2, C3), 19.3 (C, *t*Bu), 0.01 (CH_3, TMS).

These data matched those reported previously.[4]

Synthesis of 1-(*para*-methoxybenzyloxy)-5-trimethylsilyl-4-pentyn-2-ol (7b) and 1-(*para*-methoxybenzyloxy)-4-pentyn-2-ol (7c)

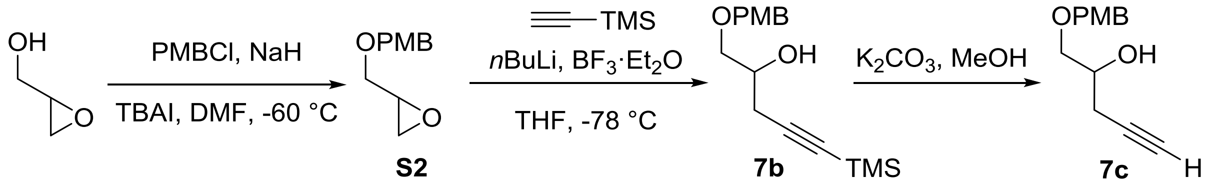

2-(*para*-Methoxybenzyloxy)methyloxirane (S2)

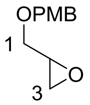

To a suspension of NaH (378 mg, 15.8 mmol) in DMF (7.8 mL) maintained between −60 and −70 °C was added, dropwise, a solution of glycidol (0.60 mL, 674 mg, 9.01 mmol) in DMF (3.5 mL). This mixture was stirred for 1 h. PMBCl (1.95 mg, 12.5 mmol, freshly prepared[1]) was added dropwise, followed by TBAI (2 mg, 0.005 mmol). The reaction was warmed up to rt, and stirred for 4 h 10 min. Reaction was quenched with H_2O (20 mL), and then extracted with Et_2O (3 × 30 mL). The organic layer was dried over $MgSO_4$, filtered and concentrated under reduced pressure. The crude product was purified by column chromatography (silica, 5:1 pet. ether/EtOAc, R_f = 0.25) to yield a colourless oil (1.18 g, 68%).

^{1}H NMR (500 MHz, $CDCl_3$): δ 7.28 (d, J = 9.3 Hz, 2H, CH, PMB), 6.89 (d, J = 8.5 Hz, 2H, CH, PMB), 4.55 (d, J = 11.5 Hz, 1H, one of CH_2, PMB), 4.49 (d, J = 11.5 Hz, 1H, one of CH_2, PMB), 3.81 (s, 3H, CH_3, PMB), 3.73 (dd, J = 11.4, 3.1 Hz, 1H, one of 1-CH_2), 3.42 (dd, J = 11.5, 5.9 Hz, 1H, one of 1-CH_2), 3.18 (ddt, J = 5.9, 4.1, 2.9 Hz, 1H, 2-CH), 2.80 (dd, J = 5.0, 4.3 Hz, 1H, one of 3-CH_2), 2.61 (dd, J = 5.1, 2.7 Hz, 1H, one of 3-CH_2).

^{13}C NMR (125 MHz, $CDCl_3$): δ 159.3 (C, PMB), 129.9 (C, PMB), 129.4 (CH, PMB), 113.8 (CH, PMB), 73.0 (CH_2, PMB), 70.5 (CH_2, C1), 55.3 (CH_3, PMB), 50.9 (CH, C2), 44.4 (CH_2, C3).

These data matched those reported previously.[5]

1-(*para*-Methoxybenzyloxy)-5-trimethylsilyl-4-pentyn-2-ol (7b)

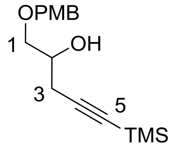

To a solution of ethynyltrimethylsilane (724 mg, 7.37 mmol) in THF (24.3 mL) at −78 °C, a solution of *n*-BuLi (4.1 mL, 1.8 M in cyclohexane, 7.4 mmol) was added dropwise. This solution was stirred for 1 h 30 min. $BF_3 \cdot Et_2O$ (0.28 mL, 2.2 mmol) was added dropwise. After 15 min, a solution of PMB-protected glycidol **S2** (1.18 g, 6.08 mmol) in THF (6.1 mL) was added dropwise. The reaction was stirred for 2 h 50 min at −78 °C. The reaction was quenched with aqueous NH_4Cl solution (10 mL), and then extracted with Et_2O (3 × 30 mL). The organic layer was dried over $MgSO_4$, and concentrated under reduced pressure. The crude product was purified by column chromatography (silica, 5:1 pet. ether/EtOAc, R_f = 0.20) to yield a colourless oil (1.48 g, 83%).

^{1}H NMR (500 MHz, $CDCl_3$): δ 7.27 (d, *J* = 8.7 Hz, 2H, CH, PMB), 6.89 (d, *J* = 8.8 Hz, 2H, CH, PMB), 4.51 (s, 2H, CH_2, PMB), 3.98–3.91 (m, 1H, 2-CH), 3.81 (s, 3H, CH_3, PMB), 3.58 (dd, *J* = 9.6, 3.9 Hz, 1H, one of CH_2, 1-CH), 3.47 (dd, *J* = 9.8, 6.5 Hz, 1H, one of CH_2, 1-CH), 2.51 (dd, *J* = 16.8, 6.0 Hz, 1H, one of 3-CH_2), 2.46 (dd, *J* = 16.8, 6.8 Hz, 1H, one of 3-CH_2), 2.42 (d, *J* = 4.1 Hz, 1H, OH), 0.14 (s, 9H, CH_3, TMS).

^{13}C NMR (125 MHz, $CDCl_3$): δ 159.3 (C, PMB), 129.9 (C, PMB), 129.4 (CH, PMB), 113.8 (CH, PMB), 102.5 (C, C4), 87.3 (C, C5), 73.1 (CH_2, PMB), 72.4 (CH_2, C1), 68.8 (CH, C2), 55.3 (CH_3, PMB), 25.0 (CH_2, C3), 0.03 (CH_3, TMS).

IR (neat) cm^{-1}: 3433 (br, OH), 2956 (m, C–H), 2921 (m, C–H), 2836 (m, C–H), 2179 (m, C≡C), 1612 (m, Ar C–C), 1247 (s, C–O), 1073 (br, C–O), 1033 (s, C–O), 839 (s, C–Si), 739 (m, C–H).

HRMS (ESI) *m/z*: found 293.1571, calcd for $C_{16}H_{25}O_3Si$ $[M+H]^+$ 293.1567 (Δ = 1.4 ppm).

The ^{1}H NMR data matched those reported previously.[6]

1-(*para*-Methoxybenzyloxy)-4-pentyn-2-ol (7c)

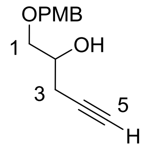

To a solution of TMS-protected alkyne **7b** (850 mg, 2.91 mmol) in MeOH (20 mL) at rt, was added solid K_2CO_3 (2.00 g, 14.5 mmol). After 4 h of stirring, reaction was diluted with H_2O (20 mL) and extracted with Et_2O (3 × 40 mL). The organic layer was dried over $MgSO_4$, and concentrated under reduced pressure. The crude product was purified by gradient column chromatography (silica, 10:1 to 5:1 pet. ether/EtOAc, R_f = 0.11 in 5:1 pet. ether/EtOAc) to yield a colourless oil (520 mg, 81%).

^{1}H NMR (500 MHz, $CDCl_3$): δ 7.27 (d, *J* = 8.1 Hz, 2H, CH, PMB), 6.89 (d, *J* = 8.3 Hz, 2H, CH, PMB), 4.51 (s, 2H, CH_2, PMB), 3.97 (m, 1H, 2-CH), 3.81 (s, 3H, CH_3, PMB), 3.59 (dd, *J* = 9.5, 3.7 Hz, 1H, one of 1-CH_2), 3.48 (dd, *J* = 9.4, 6.7 Hz, 1H, one of 1-CH_2), 2.51 (br. d, *J* = 6.2 Hz, 2H, 3-CH_2), 2.47 (d, *J* = 4.9 Hz, 1H, OH), 2.03 (br. s, 1H, 5-CH).

^{13}C NMR (125 MHz, $CDCl_3$): δ 159.3 (C, PMB), 129.9 (C, PMB), 129.4 (CH, PMB), 113.9 (CH, PMB), 80.2 (C, C4), 73.1 (CH_2, PMB), 72.5 (CH_2, C1), 70.6 (CH, C5), 68.7 (CH, C2), 55.3 (CH_3, PMB), 23.5 (CH_2, C3).

IR (neat) cm^{-1}: 3436 (br, OH), 3289 (m, C–H), 2911 (m, C–H), 2863 (m, C–H), 2180 (w, C≡C), 1612 (m, Ar C–C), 1245 (s, C–O), 1078 (m, C–O), 1031 (s, C–O), 817 (m, C–H), 638 (m, C–H).

HRMS (ESI) *m/z*: found 243.1000, calcd for $C_{13}H_{16}O_3Na$ $[M+Na]^+$ 243.0992 (Δ = 3.3 ppm).

The IR and ^{1}H NMR data matched those reported previously.[7]

Synthesis of aldehyde **8**

(Hex-1-en-5-yn-3-yloxy)*tert*-butyldimethylsilane (13)

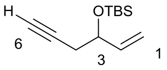

To a suspension of activated zinc powder (982 mg, 15.0 mmol) in THF (25 mL), a propargyl bromide solution (1.8 mL, 80% w/w in toluene, 16 mmol) was added dropwise. The reaction mixture was stirred at rt for 1 h 30 min. After the addition of further activated zinc powder (410 mg, 6.27 mmol), the reaction was cooled down to −78 °C. A solution of acrolein (**12**) (0.50 mL, 7.5 mmol) in THF (4.2 mL) was added dropwise at −78 °C. The reaction mixture was stirred at −78 °C for 1 h and at r.t. for 2 h. The reaction was quenched with sat. aq. NH_4Cl (20 mL) and filtered through a Celite pad. The filtrate was extracted with Et_2O (3 × 50 mL). The organic layers were combined, dried over $MgSO_4$, filtered and concentrated under reduced pressure. The yellow oil obtained was purified by column chromatography (silica, 3:1 pet. ether/EtOAc, R_f = 0.27). The relevant fractions were partially concentrated to yield a solution of the desired intermediate alcohol in EtOAc. This mixture was combined with imidazole (620 mg, 9.11 mmol) and dissolved in DMF (9.0 mL). After cooling down to 0 °C, a solution of TBSCl (1.32 g, 6.27 mmol) in DMF (9.0 mL) was added dropwise. The reaction was stirred at rt for 14 h, then diluted with Et_2O (50 mL), washed with H_2O (3 × 50 mL) and brine (50 mL), dried over $MgSO_4$, filtered and concentrated under reduced pressure. This crude product was purified by column chromatography (silica, 10:1 pet. ether/EtOAc, R_f = 0.80) to yield the TBS ether **13** as a colourless oil (1.28 g, 81% yield).

^{1}H NMR (500 MHz, $CDCl_3$): δ 5.92 (ddd, *J* = 16.8, 10.8, 5.6 Hz, 1H, 2-CH), 5.26 (d, *J* = 17.1 Hz, 1H, one of 1-CH_2), 5.12 (d, *J* = 10.5 Hz, 1H, one of 1-CH_2), 4.27 (app. q, *J* = 5.6 Hz, 1H, 3-CH), 2.42 (ddd, *J* = 16.6, 6.1, 2.9 Hz, 1H, one of 4-CH_2), 2.33 (ddd, *J* = 16.6, 7.1, 2.4 Hz, 1H, one of 4-CH_2), 1.99 (t, *J* = 2.6 Hz, 1H, 6-CH), 0.91 (s, 9H, *t*Bu), 0.10 (s, 3H, Me), 0.07 (s, 3H, Me).

^{13}C NMR (125 MHz, $CDCl_3$): δ 139.9 (CH, C2), 114.7 (CH_2, C1), 81.3 (C, C5), 72.2 (CH, C3), 69.9 (CH, C6), 28.3 (CH_2, C4), 25.8 (CH_3, *t*Bu), 18.2 (C, *t*Bu), -4.6 (CH_3, Me), -4.9 (CH_3, Me).

These data were consistent with those reported previously.[8]

Methyl 5-(*tert*-butyldimethylsilyloxy)hept-6-en-2-ynoate (14)

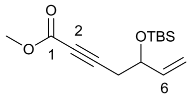

To a solution of terminal alkyne **13** (905 mg, 4.30 mmol) in THF (43 mL) at −78 °C, *n*-BuLi (2.6 mL, 1.8 M in cyclohexane, 4.68 mmol) was added dropwise. The reaction was stirred at −78 °C for 40 min. Methyl chloroformate (8.60 mmol, 0.66 mL) was added dropwise. After stirring for 2.5 h at −78 °C, the reaction was quenched with sat. aq. NH_4Cl (30 mL) and warmed up to rt. The aqueous layer was separated and extracted with Et_2O (3×30 mL). The organic layers were combined, dried over $MgSO_4$, filtered and concentrated under reduced pressure. This crude product was purified by column chromatography (silica, 30:1 pet. ether/EtOAc, R_f = 0.16) to yield the title compound **14** as a pale yellow oil (875 mg, 76%).

^{1}H NMR (500 MHz, $CDCl_3$): δ 5.87 (ddd, *J* = 16.7, 10.7, 5.9 Hz, 1H, 6-CH), 5.27 (d, *J* = 17.1 Hz, 1H, one of 7-CH_2), 5.14 (d, *J* = 10.5 Hz, 1H, one of 7-CH_2), 4.32 (app. q, *J* = 6.3 Hz, 1H, 5-CH), 3.76 (s, 3H, OCH_3), 2.55 (dd, *J* = 16.9, 6.3 Hz, 1H, one of 4-CH_2), 2.47 (dd, *J* = 16.9, 6.3 Hz, 1H, one of 4-CH_2), 0.90 (s, 9H, *t*Bu), 0.10 (s, 3H, Me), 0.06 (s, 3H, Me).

^{13}C NMR (125 MHz, $CDCl_3$): δ 154.1 (C, C1), 139.4 (CH, C6), 115.4 (CH_2, C7), 86.5 (C, C3), 74.2 (C, C2), 71.5 (CH, C5), 52.6 (OCH_3), 28.6 (CH_2, C4), 25.7 (CH_3, *t*Bu), 18.2 (C, *t*Bu), -4.6 (CH_3, Me), -5.0 (CH_3, Me).

IR (neat) cm^{-1}: 2954 (m, C–H), 2930 (m, C–H), 2857 (m, C–H), 2241 (m, C≡C), 1716 (s, C=O), 1248 (s, C–O), 1073 (s, C–H), 930 (s, C–Si), 836 (s, C–Si), 777 (s, C–Si).

HRMS (ESI) *m/z*: found 286.1837, calcd for $C_{14}H_{28}O_3SiN$ $[M+NH_4]^+$ 286.1833 (Δ = 2.1 ppm).

(2*Z*)-Methyl 5-(*tert*-butyldimethylsilyloxy)-3-methyl-2,6-heptadienoate (15)

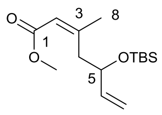

To a suspension of CuI (811 mg, 4.25 mmol) in THF (17 mL) at 0 °C, MeLi was added dropwise (3.0 mL, 2.8 M in Et_2O, 8.4 mmol). After stirring at 0 °C for 1 h, a solution of the ynoate **14** (732 mg, 2.73 mmol) in THF (1.8 mL) was added dropwise at −78 °C. The reaction mixture was stirred at −78 °C for 1 h 30 min. The reaction was quenched with sat. aq. NH_4Cl (30 mL) and warmed up to rt. The aqueous layer was separated and extracted with Et_2O (3 × 50 mL). The organic layers were combined, dried

over $MgSO_4$, filtered and concentrated under reduced pressure. This crude product was purified by column chromatography (silica, 36:1 pet. ether/ Et_2O, R_f = 0.22) to yield the title compound **15** as a yellow oil (547 mg, 70% yield).

^{1}H NMR (500 MHz, $CDCl_3$): δ 5.86 (ddd, *J* = 16.4, 10.5, 5.9 Hz, 1H, 6-CH), 5.73 (s, 1H, 2-CH), 5.20 (d, *J* = 17.1 Hz, 1H, one of 7-CH_2), 5.03 (d, *J* = 10.3 Hz, 1H, one of 7-CH_2), 4.42 (m, 1H, 5-CH), 3.68 (s, 3H, OCH_3), 2.88 (dd, *J* = 12.2, 4.6 Hz, 1H, 4-CH_2), 2.71 (dd, *J* = 12.6, 7.9 Hz, 1H, 4-CH_2), 1.95 (s, 3H, 8-CH_3), 0.88 (s, 9H, *t*Bu), 0.01 (s, 6H, Me).

^{13}C NMR (125 MHz, $CDCl_3$): δ 166.7 (C, C1), 158.4 (C, C3), 141.3 (CH, C6), 116.9 (CH, C2), 113.7 (CH_2, C7), 73.6 (CH, C5), 50.8 (OCH_3), 41.9 (CH_2, C4), 27.5 (CH_3, C8), 25.9 (CH_3, *t*Bu), 18.1 (C, *t*Bu), -4.5 (CH_3, Me), -4.9 (CH_3, Me).

IR (neat) cm^{-1}: 2952 (m, C–H), 2929 (m, C–H), 2857 (m, C–H), 1718 (s, C=O), 1645 (m, C=C), 1251 (s, C–O), 1196 (s, C–H), 1027 (s, C–H), 834 (s, C–Si), 775 (s, C–Si).

HRMS (ESI) *m/z*: found 285.1869, calcd for $C_{15}H_{29}O_3Si$ $[M+H]^+$ 285.1880 (Δ = 3.9 ppm).

(2*Z*)-5-(*tert*-Butyldimethylsilyloxy)-3-methylhepta-2,6-dien-1-ol (S3)

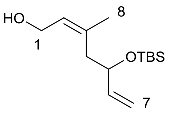

To a solution of the ester **15** (306 mg, 1.08 mmol) in DCM (10.5 mL) at −78 °C, a solution of DIBAl-H in DCM (3.5 mL, 1.0 M in THF, 3.5 mmol) was added dropwise. The reaction was stirred at −78 °C for 30 min, followed by 0 °C for 30 min and rt for 30 min. It was then quenched with a saturated aqueous solution of Rochelle’s salt (15 mL) and stirred vigorously for 1 h. The mixture was extracted with CH_2Cl_2 (3 × 15 mL). The organic layers were combined and dried over $MgSO_4$ and under reduced pressure. This crude product was purified by column chromatography (silica, 5:1 pet. ether/ EtOAc, R_f = 0.41) to yield the product **S3** as a yellow oil (229 mg, 85% yield).

^{1}H NMR (500 MHz, $CDCl_3$): δ 5.83 (ddd, *J* = 17.0, 10.5, 6.6 Hz, 1H, 6-CH), 5.65 (t, *J* = 7.1 Hz, 1H, 2-CH), 5.17 (dd, *J* = 17.3, 1.2 Hz, 1H, one of 7-CH_2), 5.06 (dd, *J* = 10.3, 1.0 Hz, 1H, one of 7-CH_2), 4.25 (m, 1H, 5-CH), 4.19 (m, 1H, one of 1-CH_2), 4.00 (app. dt, *J* = 12.1, 6.5 Hz, 1H, one of 1-CH_2), 2.51 (dd, *J* = 13.3, 8.7 Hz, 1H, one of 4-CH_2), 2.10 (dd, *J* = 13.4, 4.6 Hz, 1H, one of 4-CH_2), 1.96 (t, *J* = 5.4 Hz, 1H, OH), 1.78 (s, 3H, 8-CH_3), 0.89 (s, 9H, *t*Bu), 0.05 (s, 3H, Me), 0.05 (s, 3H, Me).

^{13}C NMR (125 MHz, $CDCl_3$): δ 141.4 (CH, C6), 136.8 (C, C3), 127.1 (CH, C2), 114.1 (CH_2, C7), 72.1 (CH, C5), 58.6 (CH_2, C1), 41.0 (CH_2, C4), 25.9 (CH_3, *t*Bu), 23.9 (CH_3, C8), 18.3 (C, *t*Bu), -4.5 (CH_3, Me), -4.7 (CH_3, Me).

IR (film from CH_2Cl_2) cm^{-1}: 3410 (br, O–H), 2956 (s, C–H), 2930 (s, C–H), 2886 (s, C–H), 2857 (s, C–H), 1678 (s, C=C), 1472 (m, C–H), 1253 (s, C–O), 937 (s, C–H), 8345 (s, C–Si), 776 (s, C–Si).

HRMS (ESI) *m/z*: found 279.1756, calcd for $C_{14}H_{28}O_2SiNa$ $[M+Na]^+$ 279.1751 (Δ = 1.8 ppm).

NOESY(600 MHz, $CDCl_3$):

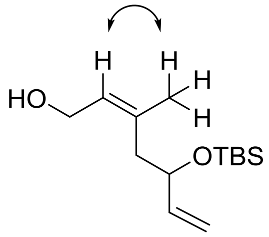

(2*Z*)-5-(*tert*-Butyldimethylsilyloxy)-3-methylhepta-2,6-dienal (8)

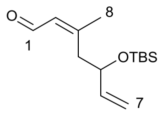

To a solution of the alcohol **S3** (374 mg, 1.46 mmol) in DMSO (2.5 mL) at rt, a solution of IBX (1.23 g, 4.37 mmol) in DMSO (18.8 mL) was added dropwise. After stirring at rt for 17 h, EtOAc (20 mL) was added to the reaction mixture to precipitate out IBX. It was filtered through celite, and the filtrate was dried under reduced pressure. This crude product was purified by column chromatography (silica, 20:1 pet. ether/ EtOAc, Rf = 0.38) to yield the aldehyde **8** as a colourless oil (306 mg, 82% yield).

^{1}H NMR (500 MHz, $CDCl_3$): δ 9.90 (d, *J* = 8.1 Hz, 1H, 1-CH=O), 5.96 (d, *J* = 7.7 Hz, 1H, 2-CH), 5.83 (dddd, *J* = 17.9, 10.3, 6.4, 2.1 Hz, 1H, 6-CH), 5.22 (d, *J* = 17.3 Hz, 1H, 7-CH_2), 5.11 (d, *J* = 10.5 Hz, 1H, 7-CH_2), 4.32 (m, 1H, 5-CH), 2.91 (ddd, *J* = 13.0, 8.2, 1.5 Hz, 1H, 4-CH_2), 2.54 (ddd, *J* = 13.1, 4.3, 1.3 Hz, 1H, 4-CH_2), 2.02 (s, 3H, 8-CH_3), 0.86 (s, 9H, *t*Bu), 0.02 (s, 3H, Me), 0.01 (s, 3H, Me).

^{13}C NMR (125 MHz, $CDCl_3$): δ 191.5 (CH, C1), 159.8 (C, C3), 140.6 (CH, C6), 130.4 (CH, C2), 114.9 (CH_2, C7), 72.6 (CH, C5), 41.5 (CH_2, C4), 26.1 (CH_3, C8), 25.8 (CH_3, *t*Bu), 18.1 (C, *t*Bu), -4.5 (CH_3, Me), -4.9 (CH_3, Me).

IR (neat) cm^{-1}: 2956 (m, C–H), 2930 (m, C–H), 2857 (m, C–H), 1676 (s, C=O), 1631 (w, C=C), 1609 (w, C=C), 1073 (s, C–O), 835 (s, C–Si), 775 (s, C–Si).

HRMS (ESI) *m/z*: found 255.1776, calcd for $C_{14}H_{27}O_2Si$ $[M+H]^+$ 255.1775 (Δ = 0.4 ppm).

NOESY (600 MHz, $CDCl_3$):

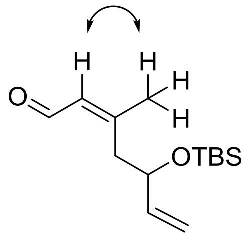

General procedure for Bestmann ylide linchpin reaction:

To a mixture of alcohol (1 eq., 0.1-0.3 M) and Bestmann ylide (1 eq.) in solvent (toluene or tetrahydrofuran) heated at reflux, a solution of aldehyde (1 eq., 1.0 M) was added. The reaction was heated at reflux until full consumption of starting material aldehyde was observed by TLC. After cooling to r.t., the reaction was concentrated and purified by silica column chromatography to afford the respective product as a colourless to very pale yellow oil.

(2′*E*,2*E*,4*E*)-Hex-2′-enyl 5-phenylpenta-2,4-dienoate (11a)

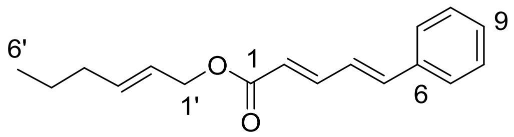

TLC: R_f = 0.23 (20:1 Pet. ether: Et_2O).

^{1}H NMR (500 MHz, $CDCl_3$): δ 7.48–7.43 (complex m, 3H, 3-CH & 7-CH), 7.36 (app. t, *J* = 7.6 Hz, 2H, 8-CH), 7.30 (t, *J* = 6.9 Hz, 1H, 9-CH), 6.82–6.93 (complex m, 2H, 4-CH & 5-CH), 6.01 (d, *J* = 15.6 Hz, 1H, 2-CH), 5.81 (dt, *J* = 15.4, 6.6 Hz, 1H, 3′-CH), 5.62 (dt, *J* = 15.4, 6.2 Hz, 1H, 2′-CH), 4.63 (d, *J* = 6.6 Hz, 2H, 1′-CH_2), 2.14 (app. q, *J* = 7.1 Hz, 2H, 4′-CH_2), 1.43 (app. sext, *J* = 7.4 Hz, 2H, 5′-CH_2), 0.92 (t, *J* = 7.3 Hz, 3H, 6′-CH_3).

^{13}C NMR (125 MHz, $CDCl_3$): δ 166.8 (C, C=O), 144.7 (CH, C3), 140.4 (CH, C5), 136.3 (CH, C3′), 136.0 (C, C6), 129.0 (CH, C9), 128.8 (CH, C8), 127.2 (CH, C7), 126.2 (CH, C4),

124.0 (CH, C2′), 121.2 (CH, C2), 65.2 (CH_2, C1′), 34.3 (CH_2, C4′), 22.0 (CH_2, C5′), 13.6 (CH_3, C6′).

IR (neat) cm^{-1}: 2958 (m, C–H), 2929 (m, C–H), 1706 (s, C=O), 1625 (s, C=C), 1449 (m, C–H), 1236 (s, C–O), 1172 (s, C–O), 997 (m, C–H), 689 (m, C–H).

HRMS (ESI) *m/z*: found 257.1529, calcd for $C_{17}H_{21}O_2$ $[M+H]^+$ 257.1536 (Δ = 2.7 ppm).

(2′*E*,2*E*,4*E*)-Oct-2′-enyl 5-phenylpenta-2,4-dienoate (11b)

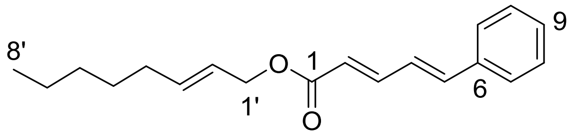

TLC: R_f = 0.08 (40:1 pet. ether: Et_2O).

^{1}H NMR (500 MHz, $CDCl_3$): δ 7.57–7.50 (complex m, 3H, 3-CH & 7-CH), 7.35 (t, *J* = 7.3 Hz, 2H, 8-CH), 7.31 (t, *J* = 7.3 Hz, 1H, 9-CH), 6.93–6.82 (complex m, 2H, 4-CH & 5-CH), 6.01 (d, *J* = 15.4 Hz, 1H, 2-CH), 5.81 (dt, *J* = 15.0, 7.0 Hz, 1H, 3′-CH), 5.62 (dt, *J* = 15.1, 6.6 Hz, 1H, 2′-CH), 4.62 (d, *J* = 6.3 Hz, 2H, 1′-CH_2), 2.07 (app. q, *J* = 7.1 Hz, 2H, 4′-CH_2), 1.40 (app. quin *J* = 7.3 Hz, 2H, 5′-CH_2), 1.34–1.24 (complex m, 4H, 6′-CH_2 & 7′-CH_2), 0.90 (t, *J* = 6.8 Hz, 3H, 8′-CH_3).

^{13}C NMR (125 MHz, $CDCl_3$): δ 166.8 (C, C1), 144.7 (CH, C3), 140.4 (CH, C5), 136.7 (CH, C3′), 136.0 (C, C6), 129.0 (CH, C9), 128.8 (CH, C8), 127.2 (CH, C7), 126.2 (CH, C4), 123.8 (CH, C2′), 121.2 (CH, C2), 65.3 (CH_2, C1′), 32.3 (CH_2, C4′), 31.4 (CH_2, C6′), 28.6 (CH_2, C5′), 22.5 (CH_2, C7′), 14.0 (CH_3, C8′).

IR (neat) cm^{-1}: 2956 (m, C–H), 2955 (m, C–H), 1709 (s, br, C=O), 1625 (s, C=C), 1235 (s, C–O), 1129 (s, C–O), 969 (s, C–H), 691 (m, C–H).

HRMS (ESI) *m/z*: found 307.1666, calcd for $C_{19}H_{24}O_2Na$ $[M+Na]^+$ 307.1669 (Δ = 0.9 ppm).

(2′*Z*,2*E*,4*E*)-Hex-2′-enyl 5-phenylpenta-2,4-dienoate (11c)

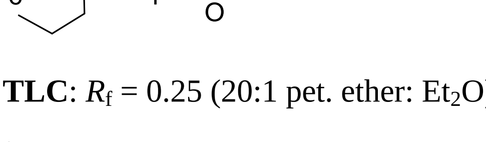

TLC: R_f = 0.25 (20:1 pet. ether: Et_2O).

^{1}H NMR (500 MHz, $CDCl_3$): δ 7.49–7.44 (complex m, 3H, 3-CH & 7-CH), 7.36 (app. t, *J* = 7.6 Hz, 2H, 8-CH), 7.30 (t, *J* = 6.9 Hz, 1H, 9-CH), 6.98–6.83 (complex m, 2H, 4-CH & 5-

CH), 6.03 (d, *J* = 14.9 Hz, 1H, 2-CH), 5.69 (dt, *J* = 11.0, 7.5 Hz, 1H, 2′-CH), 5.63 (dt, *J* = 11.8, 6.4 Hz, 1H, 3′-CH), 4.76 (d, *J* = 7.2 Hz, 2H, 1′-CH), 2.14 (app. q, *J* = 7.2 Hz, 2H, 4′-CH), 1.45 (app. sext, *J* = 7.2 Hz, 2H, 5′-CH), 0.95 (t, *J* = 7.3 Hz, 3H, 6′-CH).

^{13}C NMR (125 MHz, $CDCl_3$): δ 167.0 (C, C1), 144.7 (CH, C3), 140.5 (CH, C5), 136.0 (C, C6), 135.2 (CH, C2′), 129.0 (CH, C9), 128.8 (CH, C8), 127.2 (CH, C7), 126.2 (CH, C4), 123.6 (CH, C3′), 121.1 (CH, C2), 60.3 (CH_2, C1′), 29.6 (CH_2, C4′), 22.6 (CH_2, C5′), 13.7 (CH_3, C6′).

IR (neat) cm^{-1}: 2959 (m, C–H), 2930 (m, C–H), 1707 (s, C=O), 1624 (s, C=C), 1449 (m, C–H), 1234 (s, C–O), 997 (s, C–H), 690 (m, C–H).

HRMS (ESI) *m/z*: found 257.1527, calcd for $C_{17}H_{21}O_2$ $[M+H]^+$ 257.1536 (Δ = 3.5 ppm).

(2*E*,4*E*)-Oct-1′-en-3′-yl 5-phenylpenta-2,4-dienoate (11d)

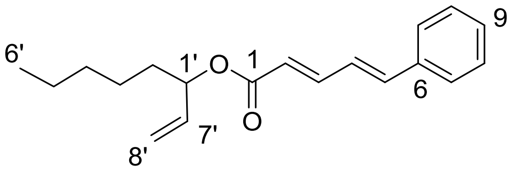

TLC: R_f = 0.16 (40:1 pet. ether: Et_2O).

^{1}H NMR (500 MHz, $CDCl_3$): δ 7.47 (d, *J* = 7.8 Hz, 2H, 7-CH), 7.46 (dd, *J* = 14.9, 8.8 Hz, 1H, 3-CH), 7.36 (app. t, *J* = 7.6 Hz, 2H, 8-CH), 7.31 (t, *J* = 7.2 Hz, 1H, 9-CH), 6.95–6.84 (complex m, 2H, 4-CH & 5-CH), 6.02 (d, *J* = 14.9 Hz, 1H, 2-CH), 5.83 (ddd, *J* = 17.1, 10.6, 6.2 Hz, 1H, 7′-CH), 5.34 (app. q, *J* = 6.6 Hz, 1H, 1′-CH), 5.27 (dd, *J* = 17.3, 1.2 Hz, 1H, one of 8′-CH_2), 5.18 (dd, *J* = 10.5, 1.0 Hz, 1H, one of 8′-CH_2), 1.74–1.59 (complex m, 2H, 2′-CH_2), 1.39–1.26 (complex m, 6H, 3′-CH_2 & 4′-CH_2 & 5′-CH_2), 0.89 (t, *J* = 6.1Hz, 3H, 6′-CH_3).

^{13}C NMR (125 MHz, $CDCl_3$): δ 166.3 (C, C1), 144.6 (CH, C3), 140.4 (CH, C5), 136.7 (CH, C7′), 136.0 (C, C6), 129.0 (CH, C9), 128.8 (CH, C8), 127.2 (CH, C7), 126.2 (CH, C4), 121.5 (CH, C2), 116.4 (CH_2, C8′), 74.7 (CH, C1′), 34.3 (CH_2, C2′), 31.6 and 24.8 and 22.5 (CH_2, C3′ and C4′ and C5′), 14.0 (CH_3, C6′).

IR (neat) cm^{-1}: 2930 (m, C–H), 2859 (m, C–H), 1706 (s, C=O), 1625 (s, C=C), 1235 (s, C–O), 1130 (s, C–O), 996 (s, C–H), 688 (m, C–H).

HRMS (ESI) *m/z*: found 307.1667, calcd for $C_{19}H_{24}O_2Na$ $[M+Na]^+$ 307.1669 (Δ = 0.65 ppm).

(1′*R*,2′*S*,5′*R*,2*E*,4*E*)-2′-Isopropyl-5′-methylcyclohex-1′-yl 5-phenylpenta-2,4-dienoate (11e)

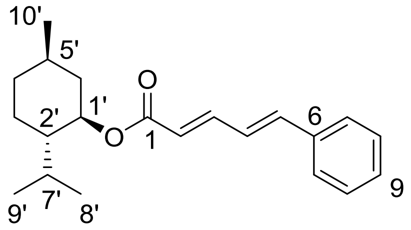

TLC: R_f = 0.14 (40:1 pet. ether: Et_2O).

^{1}H NMR (500 MHz, $CDCl_3$): δ 7.46 (d, *J* = 7.8 Hz, 2H, 7-CH), 7.43 (partially obs. ddd, *J* = 15.3, 9.2, 1.0 Hz, 1H, 3-CH), 7.36 (app. t, *J* = 7.6 Hz, 2H, 8-CH), 7.30 (tt, *J* = 7.3, 2.4 Hz, 1H, 9-CH), 6.93–6.83 (complex m, 2H, 4-CH & 5-CH), 5.99 (d, *J* = 15.4 Hz, 1H, 2-CH), 4.79 (td, *J* = 10.9, 4.4 Hz, 1H, 1′-CH), 2.05 (dddd, *J* = 11.9, 4.4, 3.0, 2.0 Hz, 1H, one of 6′-CH_2), 1.91 (septd, *J* = 7.0, 2.7 Hz, 1H, 7′-CH), 1.74–1.66 (complex m, 2H, one of 3′-CH_2 & one of 4′-CH_2), 1.53 (m, 1H, 5′-CH), 1.43 (tt, *J* = 11.7, 2.9 Hz, 1H, 2′-CH), 1.09 (qd, *J* = 13.2, 3.3 Hz, 1H, one of 4′-CH_2), 1.02 (q, *J* = 11.5 Hz, 1H, one of 6′-CH_2), 0.92 (d, *J* = 6.6 Hz, 3H, 10′-CH_3), 0.91 (d, *J* = 7.1 Hz, 3H, 8′-CH_3), 0.94–0.84 (partially obscured m, 1H, one of 3′-CH_2), 0.78 (d, *J* = 7.1 Hz, 3H, 9′-CH_3).

^{13}C NMR (125 MHz, $CDCl_3$): δ 166.6 (C, C1), 144.3 (CH, C3), 140.2 (CH, C5), 136.1 (C, C6), 129.0 (CH, C9), 128.8 (CH, C8), 127.1 (CH, C7), 126.3 (CH, C4), 121.9 (CH, C2), 74.1 (CH, C1′), 47.2 (CH, C2′), 41.0 (CH_2, C6′), 34.3 (CH_2, C3′), 31.4 (CH, C5′), 26.3 (CH, C7′), 23.6 (CH_2, C4′), 22.0 (CH_3, C10′), 20.8 (CH_3, C8′), 16.5 (CH_3, C9′).

IR (neat) cm^{-1}: 2953 (m, C–H), 2925 (m, C–H), 2868 (m, C–H), 1702 (s, C=O), 1625 (s, C=C), 1237 (s, C–Si), 1131 (s, C–O), 995 (s, C–H), 754 (s, C–Si), 689 (s, C–Si).

HRMS (ESI) *m/z*: found 335.1969, calcd for $C_{21}H_{28}O_2Na$ $[M+Na]^+$ 335.1982 (Δ = 3.9 ppm).

Specific rotation: $[\alpha]_D^{22°C}$ = -60 (*c* = 0.40, CH_2Cl_2).

This compound has been reported previously.[9] There is close correlation between the IR and ^{13}C NMR data entered above and those in the earlier report. However, the ^{1}H NMR data quoted previously do not match those obtained by us. We are very confident of the identity of the sample prepared in our work and all NMR assignments were made on the basis of thorough 2D NMR experiments (COSY and HSQC). We can only suppose that the 90 MHz instrument used in the earlier work did not allow accurate identification of the ^{1}H NMR signals. Our HRMS data provide further assurance of the identity of the sample we prepared; the earlier report does not indicate either HRMS or elemental analytical results.

(1′*R*,2′*S*,5′*R*,2*E*,4*E*,6*E*)- 2′-*iso*-Propyl-5′-methylcyclohex-1′-yl deca-2,4,6-trienoate (11f)

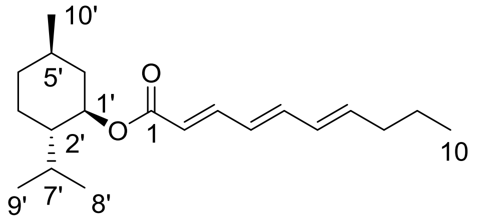

TLC: R_f = 0.14 (40:1 Pet. ether: Et_2O).

^{1}H NMR (500 MHz, $CDCl_3$): δ 7.28 (dd, *J* = 15.4, 11.2 Hz, 1H, 3-CH), 6.53 (dd, *J* = 14.9, 10.7 Hz, 1H, 5-CH), 6.21 (dd, *J* = 14.9, 11.2 Hz, 1H, 4-CH), 6.13 (dd, *J* = 15.1, 10.7 Hz, 1H, 6-CH), 5.92 (dt, *J* = 14.9, 7.2 Hz, 1H, 7-CH), 5.83 (d, *J* = 15.4 Hz, 1H, 2-CH), 4.75 (app. td, *J* = 10.9, 4.4 Hz, 1H, 1′-CH), 2.12 (app. q, *J* = 7.3 Hz, 2H, 8-CH_2), 2.02 (br d, *J* = 12.2 Hz, 1H, one of 6′-CH_2), 1.88 (septd, *J* = 6.8, 2.3 Hz, 1H, 7′-CH), 1.65–1.71 (complex m, 2H, one of 3′-CH_2 & one of 4′-CH), 1.50 (partially obs. m, 1H, 5′-CH_2), 1.44 (m, 2H, 9-CH_2), 1.40 (partially obs. m, 1H, 2′-CH), 1.07 (app. qd, *J* = 12.9, 2.9 Hz, 1H, one of 4′-CH_2), 0.99 (app. q, *J* = 11.5 Hz, 1H, one of 6′-CH_2), 0.91 (t, *J* = 7.3 Hz, 3H, 10-CH_3), 0.90 (d, *J* = 6.1 Hz, 3H, 10′-CH_3), 0.89 (d, *J* = 6.8 Hz, 3H, 8′-CH_3), 0.87 (partially obs. m, 1H, one of 3′-CH_2), 0.76 (d, *J* = 7.1 Hz, 3H, 9′-CH_3).

^{13}C NMR (125 MHz, $CDCl_3$): δ 166.8 (C, C1), 144.6 (CH, C3), 141.0 (CH, C5), 140.2 (CH, C7), 130.0 (CH, C6), 127.8 (CH, C4), 120.5 (CH, C2), 73.9 (CH, C1′), 47.2 (CH, C2′), 41.0 (CH_2, C6′), 35.0 (CH_2, C8), 34.3 (CH_2, C3′), 31.4 (CH, C5′), 26.3 (CH, C7′), 23.6 (CH_2, C4′), 22.2 (CH_2, C9), 22.0 (CH_3, C10′), 20.7 (CH_3, C8′), 16.4 (CH_2, C9′), 13.7 (CH_3, C10).

IR (neat) cm^{-1}: 2955 (s, C–H), 2930 (s, C–H), 2869 (s, C–H), 1694 (s, C=O), 1615 (s, C=C), 1456 (m, C–H), 1342 (m, C–H), 1133 (s, C–O), 1007 (s, C–H).

HRMS (ESI) *m/z*: found 305.2486, caled for $C_{20}H_{33}O_2$ $[M+H]^+$ 305.2475 (Δ = 3.6 ppm).

Specific rotation: $[\alpha]_D^{22°C}$ = -26 (*c* = 0.42, CH_2Cl_2).

Mixture of (2*E*,4*E*)-[1′-(*tert*-butyldiphenylsilyloxy)-5′-trimethylsilyl]pent-4′-yn-2′-yl 5-phenylpenta-2,4-dienoate (11g) and the silyl migrated product (2*E*,4*E*)-[2′-(*tert*-butyldiphenylsilyloxy)-5′-trimethylsilyl]pent-4′-yn-1′-yl 5-phenylpenta-2,4-dienoate (3:2)

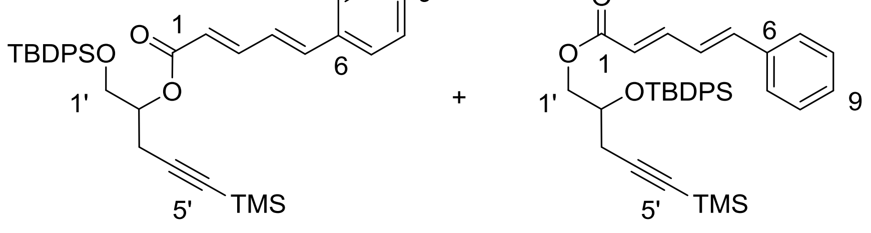

Major (A) **Minor (B)**

TLC: R_f = 0.14 (36:1 pet. ether: Et_2O).

^{1}H NMR (500 MHz, $CDCl_3$): δ 7.75–7.67 (complex m, 4H, CH, Ph), 7.55–7.30 (complex m, 11H, CH, Ph), 7.47 (obs. m, 0.6H, 3-CH-A), 7.32 (obs. m, 0.4H, 3-CH-B), 6.94–6.79 (complex m, 2H, 4-CH & 5-CH), 5.99 (d, *J* = 15.3 Hz, 0.6H, 2-CH-A), 5.83 (d, *J* = 15.6 Hz, 0.4H, 2-CH-B), 5.16 (app. quin, *J* = 5.6 Hz, 0.6H, 2′-CH-A), 4.23 (dd, *J* = 11.4, 4.4 Hz, 0.4H, one of 1′-CH_2-B), 4.20 (dd, *J* = 11.2, 5.9 Hz, 0.4H, one of 1′-CH_2-B), 4.10 (app. quin, *J* = 5.3 Hz, 0.4H, 2′-CH-B), 3.91 (dd, *J* = 11.2, 5.0 Hz, 0.6H, one of 1′-CH_2-A), 3.86 (dd, *J* = 10.9, 4.1 Hz, 0.6H, one of 1′-CH_2-A), 2.77 (dd, *J* = 16.7, 7.0 Hz, 0.6H, one of 3′-CH_2-A), 2.67 (dd, *J* = 16.7, 5.9 Hz, 0.6H, one of 3′-CH_2-A), 2.47 (dd, *J* = 17.0, 6.5 Hz, 0.4H, one of 3′-CH_2-B), 2.43 (dd, *J* = 17.0, 5.1 Hz, 0.4H, one of 3′-CH_2-B), 1.08 (s, 2.7H, CH_3, *t*Bu-B), 1.06 (s, 5.4H, CH_3, *t*Bu-A), 0.14 (s, 2.7H, CH_3, Me-B), 0.13 (s, 5.4H, CH_3, Me-A).

^{13}C NMR (125 MHz, $CDCl_3$): δ 166.6 (C, C1-B), 166.1 (C, C1-A), 145.0 (CH, C3-A), 144.8 (CH, C3-B), 140.6 (CH, C5-A), 140.5 (CH, C5-B), 136.0 (CH, Ph), 135.9 (CH, C6), 135.8 (CH, Ph), 135.54 (CH, Ph), 135.52 (CH, Ph), 133.7 (C, Ph), 133.5 (C, Ph), 133.3 (C, Ph), 129.8 (CH, Ph), 129.7 (CH, Ph), 129.08 (CH, C7-A), 129.06 (CH, C7-B), 128.8 (CH, C9), 127.70 (CH, Ph), 127.68 (CH, Ph), 127.65 (CH, Ph), 127.59 (CH, Ph), 127.21 (CH, C8-A), 127.18 (CH, C8-B), 126.22 (CH, C4-A), 126.18 (CH, C4-B), 121.1 (CH, C2-A), 120.8 (CH, C2-B), 102.6 (C, C4′-B), 102.1 (C, C4′-A), 87.2 (C, C5′-B), 86.9 (C, C5′-A), 72.1 (CH, C2′-A), 69.6 (CH, C2′-B), 66.9 (CH_2, C1′-B), 63.7 (CH_2, C1′-A), 26.9 (CH_3, *t*Bu-B), 26.7 (CH_3, *t*Bu-A), 25.8 (CH_2, C3′-B), 21.9 (CH_2, C3′-A), 19.4 (C, *t*Bu-B), 19.3 (C, *t*Bu-B), -0.01 (CH_3, Me).

IR (neat) cm^{-1}: 3071 (w, C–H), 2958 (m, C–H), 2857 (m, C–H), 2178 (m, C≡C), 1710 (s, C=O), 1625 (s, C=C), 1235 (s, Si–C), 1112 (s, C–O), 997 (s, C–H), 840 (s, Si–C), 700 (s, Si–C), 504 (s, Si–C).

HRMS (ESI) *m/z*: found 605.2314, calcd for $C_{35}H_{42}O_3Si_2K$ $[M+K]^+$ 605.2304 (Δ = -1.7 ppm).

The three-bond C–H correlations across the esters in both compounds were confirmed by CIGAR NMR.

(2*E*,4*E*)-[1′-(*para*-Methoxybenzyloxy)-5′-trimethylsilyl]pent-4′-yn-2′-yl 5-phenylpenta-2,4-dienoate (11h)

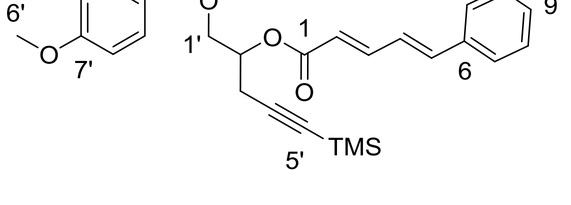

TLC: R_f = 0.28 (80% CH_2Cl_2 in *n*-hexane).

^{1}H NMR (500 MHz, $CDCl_3$): δ 7.51–7.44 (complex m, 3H, 3-CH & 8-CH), 7.37 (app. t, *J* = 7.1 Hz, 2H, 7-CH), 7.33 (m, 1H, 9-CH), 7.28 (br d, *J* = 8.8 Hz, 2H, 9′-CH), 6.95–6.84 (complex m, 4H, 8′-CH & 4-CH & 5-CH), 6.02 (d, *J* = 15.4 Hz, 1H, 2-CH), 5.20 (app. dt, *J* = 10.5, 5.7 Hz, 1H, 2′-CH), 4.55 (d, *J* = 11.5 Hz, 1H, one of 11′-CH_2), 4.50 (d, *J* = 11.7 Hz, 1H, one of 11′-CH_2), 3.80 (s, 3H, 6′-CH_3), 3.72–3.67 (m, 2H, 1′-CH_2), 2.68 (dd, *J* = 17.1, 7.1 Hz, 1H, one of 3′-CH_2), 2.63 (dd, *J* = 16.9, 5.9 Hz, 1H, one of 3′-CH_2), 0.14 (s, 9H, CH_3, TMS).

^{13}C NMR (125 MHz, $CDCl_3$): δ 166.2 (C, C1), 159.2 (C, C7′), 145.2 (CH, C3), 140.7 (CH, C5), 136.0 (C, C6), 130.1 (C, C10′), 129.3 (CH, C9′), 129.1 (CH, C7), 128.8 (CH, C9), 127.2 (CH, C8), 126.2 (CH, C4), 121.0 (CH, C2), 113.8 (CH, C8′), 101.9 (C, C4′), 87.1 (C, C5′), 73.0 (CH_2, C11′), 70.6 (CH, C2′), 69.4 (CH_2, C1′), 55.2 (CH_3, C6′), 22.3 (CH_2, C3′), -0.03 (CH_3, TMS).

IR (neat) cm^{-1}: 3028 (w, C–H), 2957 (m, C–H), 2901 (m, C–H), 2178 (m, C≡C), 1709 (s, C=O), 1625 (s, C=C), 1512 (m, C–O), 1245 (s, C–O), 1128 (s, C–O), 840 (s, C–Si), 757 (s, C–Si).

HRMS (ESI) *m/z*: found 449.2146, calcd for $C_{27}H_{33}O_4Si$ $[M+H]^+$ 449.2143 (Δ = 0.67 ppm).

Reactions of zampanolide/dactylolide fragments with Bestmann ylide linchpin

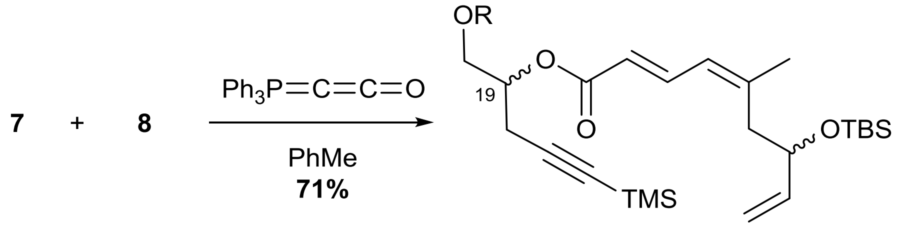

5

Mixture of (2*E*,4*Z*)-1′-(*tert*-butyldiphenylsilyloxy)-5′-(trimethylsilyl)pent-4′-yn-2′-yl 7-(*tert*-butyldimethylsilyloxy)-5-methylnona-2,4,8-trienoate (5a) and the silyl migrated product, (2*E*,4*Z*)-2′-(*tert*-butyldiphenylsilyloxy)-5′-(trimethylsilyl)pent-4′-yn-1′-yl 7-(*tert*-butyldimethylsilyloxy)-5-methylnona-2,4,8-trienoate (2:1)

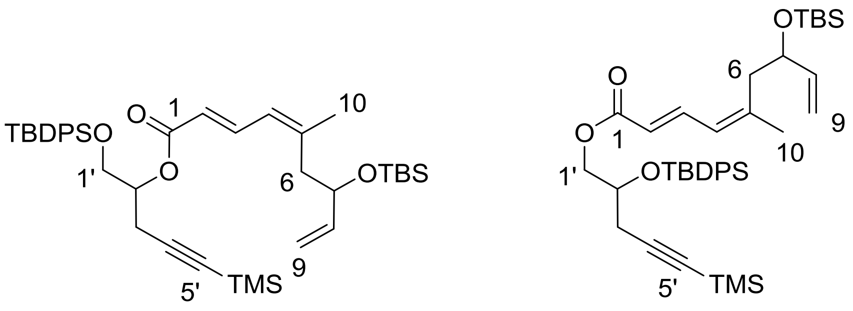

Major (A) **Minor (B)**

TLC: R_f = 0.21 (40:1 pet. ether:Et_2O).

^{1}H NMR (500 MHz, $CDCl_3$): δ 7.75–7.64 (complex m, 4H, CH, Ph), 7.57 (partially obs. ddd, *J* = 15.3, 11.6, 3.9 Hz, 0.67H, 3-CH-A), 7.51 (partially obs. ddd, *J* = 15.4, 11.7, 3.9 Hz, 0.33H, 3-CH-B), 7.46–7.34 (complex m, 6H, CH, Ph), 6.09 (d, *J* = 11.7 Hz, 0.67H, 4-CH-A), 6.04 (d, *J* = 11.7 Hz, 0.33H, 4-CH-B), 5.85–5.74 (complex m, 1.67H, 8-CH & 2-CH-A), 5.64 (d, *J* = 15.1 Hz, 0.33H, 2-CH-B), 5.22–5.10 (complex m, 1.67H, 2′-CH-A & one of 9-CH_2), 5.07–5.00 (complex m, 1H, one of 9-CH_2), 4.25 (dt, *J* = 6.6, 5.9 Hz, 1H, 7-CH), 4.18 (dd, *J* = 5.1, 1.5 Hz, 0.66H, 1′-CH_2-B), 4.09–4.04 (complex m, 0.33H, 2′-CH), 3.89 (ddd, *J* = 11.0, 4.6, 2.4 Hz, 0.67H, one of 1′-CH_2-A), 3.84 (ddd, *J* = 11.0, 4.4, 1.7 Hz, 0.67H, one of 1′-CH_2-A), 2.75 (ddd, *J* = 16.9, 7.1, 1.5 Hz, 0.67H, one of 3′-CH_2-A), 2.64 (obs. ddd, *J* = 16.9, 5.9, 4.2 Hz, 0.67H, one of 3′-CH_2-A), 2.60–2.52 (complex m, 1H, one of 6-CH_2), 2.44–2.39 (complex m, 0.66H, 3′-CH_2-B), 2.36 (dd, *J* = 13.4, 5.4 Hz, 1H, one of 6-CH_2), 1.93 (s, 2.0H, 10-CH_3-A), 1.92 (s, 1.0H, 10-CH_3-B), 1.07 (s, 3.0H, CH_3, *t*Bu, TBDPS-B), 1.05 (s, 6.0H, CH_3, *t*Bu, TBDPS-A), 0.86 (s, 3.0H, CH_3, *t*Bu, TBS-B), 0.86 (s, 6.0H, CH_3, *t*Bu, TBS-A), 0.13 (s, 3.0H, CH_3, TMS-B), 0.12 (s, 6.0H, CH_3, TMS-A), 0.01 (s, 6H, CH_3, Me).

^{13}C NMR (500 MHz, $CDCl_3$): δ 167.14 and 167.13 (C, C1-B), 166.67 and 166.64 (C, C1-A), 146.53 and 146.50 and 146.45 (C, C5), 141.48 and 141.46 (CH, C3-A), 141.29 (CH, C3-B), 140.89 and 140.87 and 140.83 (CH, C8), 135.9 (CH, Ph), 135.8 (CH, Ph), 135.60 (CH, Ph), 135.57 (CH, Ph), 135.53 (CH, Ph), 135.50 (CH, Ph), 135.49 (CH, Ph), 133.61 and 133.60 (C, Ph), 133.56 (C, Ph), 133.4 (C, Ph), 133.3 (C, Ph), 129.74 (CH, Ph), 129.67 (CH, Ph), 129.65 (CH, Ph), 129.64 (CH, Ph), 129.59 (CH, Ph), 129.31 (CH, Ph), 127.68 (CH, Ph), 127.66 (CH, Ph), 127.63 (CH, Ph), 127.61 (CH, Ph), 127.57 (CH, Ph), 126.09 and 126.05 and 126.02 (CH, C4), 119.01 and 118.95 (CH, C2-A), 118.76 and 118.75 (CH, C2-B), 114.2 (CH_2, C9), 102.73 and 102.71 (C, C4′-B), 102.22 and 102.20 (C, C4′-A), 87.0 (C, C5′-B), 86.76 and 86.75 (C, C5′-A), 72.85 (CH, C7-B), 72.83 (CH, C7-A), 71.86 and 71.82 (CH, C2′-A), 69.52 (CH, C2′-B), 66.58 and 66.53 (CH_2, C1′-B), 63.71 and 63.68 (CH_2, C1′-A), 41.7 (CH_2, C6), 26.9 (CH_3, *t*Bu), 26.73 (CH_3, *t*Bu), 26.71 (CH_3, *t*Bu), 25.82 and 25.81 (CH_3, C10-A), 25.67 (CH_3, C10-B), 25.65 and 25.64 (CH_2, C3′-B), 21.9 (CH_2, C3′-A), 19.4 (C, *t*Bu), 19.3 (C, *t*Bu), 19.2 (C, *t*Bu), 18.1 (C, *t*Bu), 1.02 (CH_3, Me), 0.00 (CH_3, Me), -0.01 (CH_3, Me), -0.04 (CH_3, Me), -4.5 (CH_3, Me), -4.88 (CH_3, Me), -4.90 (CH_3, Me).

IR (Et_2O film) cm^{-1}: 3072 (w, C–H), 2959 (s, C–H), 2858 (s, C–H), 2179 (m, C≡C), 1716 (m, C=O), 1637 (m, C=C), 1251 (m, C–Si), 1114 (s, Si–O), 776 (m, Si–Me), 702 (s, Si–C).

HRMS (ESI) *m/z*: found 689.3838, calcd for $C_{40}H_{61}O_4Si_3$ $[M+H]^+$ 689.3872 (Δ = -4.8 ppm).

The three-bond C-H correlations across the esters in both compounds were confirmed by CIGAR NMR.

(2*E*,4*Z*)-1′-(*para*-Methoxybenzyloxy)-5′-(trimethylsilyloxy)pent-4′-yn-2′-yl 7-(*tert*-butyldimethylsilyloxy)-5-methylnona-2,4,8-trienoate (5b)

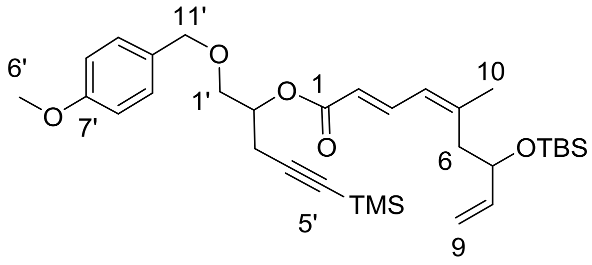

TLC: R_f = 0.30 (80% CH_2Cl_2 in *n*-hexane).

^{1}H NMR (500 MHz, $CDCl_3$): δ 7.57 (dd, *J* = 14.9, 11.8 Hz, 1H, 3-CH), 7.26 (d, *J* = 8.3 Hz, 2H, 9′-CH), 6.87 (d, *J* = 8.3 Hz, 2H, 8′-CH), 6.07 (d, *J* = 11.5 Hz, 1H, 4-CH), 5.80 (obs. ddd, *J* = 16.8, 10.6, 5.7 Hz, 1H, 8-CH), 5.80 (obs. d, *J* = 15.2 Hz, 1H, 2-CH), 5.23–5.11 (obs. m, 1H, 2′-CH), 5.18 (obs. d, *J* = 17.2 Hz, 1H, one of 9-CH_2), 5.05 (d, *J* = 10.3 Hz, 1H, one of 9-CH_2), 4.53 (d, *J* = 11.5 Hz, 1H, one of 11′-CH_2), 4.48 (d, *J* = 11.5 Hz, 1H, one of 11′-CH_2), 4.25 (app. q, *J* = 6.2 Hz, 1H, 7-CH), 3.80 (s, 3H, 6′-CH_3), 3.67 (app. d, *J* = 4.9 Hz, 2H, 1′-CH_2), 2.69–2.54 (complex m, 3H, 3′-CH_2 & one of 6-CH_2), 2.36 (ddd, *J* = 13.0, 6.5, 5.5 Hz, 1H, one of 6-CH_2), 1.92 (s, 3H, 10-CH_3), 0.87 (s, 9H, CH_3, *t*Bu), 0.13 (s, 9H, CH_3, TMS), 0.01 (s, 6H, CH_3, Me).

^{13}C NMR (500 MHz, $CDCl_3$): δ 166.70 and 166.69 (C, C1), 159.2 (C, C7′), 146.58 and 146.56 (C, C5), 141.70 and 141.67 (CH, C3), 140.9 (CH, C8), 130.1 (C, C10′), 129.3 (CH, C9′), 126.09 and 129.07 (CH, C4), 118.89 and 118.87 (CH, C2), 114.2 (CH_2, C9), 113.8 (CH, C8′), 102.0 (C, C4′), 86.92 and 86.90 (C, C5′), 73.00 and 72.99 (CH_2, C11′), 72.86 and 72.82 (CH, C7), 70.48 and 70.47 (CH, C2′), 69.45 and 69.41 (CH_2, C1′), 55.2 (CH_3, C6′), 41.7 (CH_2, C6), 25.83 and 25.80 (CH_3, *t*Bu), 25.65 and 25.62 (CH_3, C10), 22.32 and 22.29 (CH_2, C3′), 18.1 (C, *t*Bu), 0.00 and -0.01 (CH_3, TMS), -4.54 and -4.88 (CH_3, TBS).

IR (neat) cm^{-1}: 2956 (s, C–H), 2857 (m, C–H), 2180 (w, C≡C), 1714 (s, C=O), 1636 (m, C=C), 1513 (m, C=C), 1249 (s, C–O), 1033 (m, C-H), 837 (s, C–Si), 775 (m, C–Si), 760 (m, C–Si).

HRMS (ESI) *m/z*: found 571.3276, calcd for $C_{32}H_{51}O_5Si_2$ $[M+H]^+$ 571.3270 (Δ = 1.1 ppm).

(2*E*,4*Z*)-1′-(*para*-Methoxybenzyloxy)pent-4′-yn-2′-yl 7-(*tert*-butyldimethylsilyloxy)-5-methylnona-2,4,8-trienoate (5c)

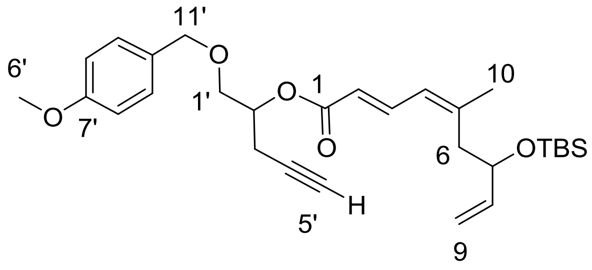

TLC: R_f = 0.32 (80% CH_2Cl_2 in *n*-hexane).

^{1}H NMR (500 MHz, $CDCl_3$): δ 7.58 (dd, *J* = 14.9, 11.7 Hz, 1H, 3-CH), 7.26 (d, *J* = 8.6 Hz, 2H, 9′-CH), 6.87 (d, *J* = 8.6 Hz, 2H, 8′-CH), 6.07 (d, *J* = 11.5 Hz, 1H, 4-CH), 5.80 (obs. ddd, *J* = 17.1, 10.5, 5.9 Hz, 1H, 8-CH), 5.80 (obs. d, *J* = 14.9 Hz, 1H, 2-CH), 5.23–5.14 (obs. m, 1H, 2′-CH), 5.18 (obs. d, *J* = 16.9 Hz, 1H, one of 9-CH_2), 5.05 (d, *J* = 10.3 Hz, 1H, one of 9-CH_2), 4.53 (d, *J* = 11.5 Hz, 1H, one of 11′-CH_2), 4.48 (d, *J* = 11.5 Hz, 1H, one of 11′-CH_2), 4.25 (app. q, *J* = 5.6 Hz, 1H, 7-CH), 3.80 (s, 3H, 6′-CH_3), 3.71–3.62 (m, 2H, 1′-CH_2), 2.65 (ddd, *J* = 16.9, 6.6, 2.7 Hz, 1H, one of 3′-CH_2), 2.58 (ddd, *J* = 16.9, 5.4, 2.5 Hz, 1H, one of 3′-CH_2), 2.61–2.54 (obs. m, 1H, one of 6-CH_2), 2.36 (app. dt, *J* = 13.1, 4.9 Hz, 1H, one of 6-CH_2), 1.97 (m, 1H, 5′-CH), 1.92 (s, 3H, 10-CH_3), 0.86 (s, 9H, CH_3, *t*Bu), 0.01 (s, 3H, CH_3, Me), 0.00 (s, 3H, CH_3, Me).

^{13}C NMR (500 MHz, $CDCl_3$): δ 166.7 (C, C1), 159.2 (C, C7′), 146.79 and 146.76 (C, C5), 141.90 and 141.88 (CH, C3), 140.93 and 140.92 (CH, C8), 130.0 (C, C10′), 129.29 and 129.28 (CH, C9′), 126.06 and 126.03 (CH, C4), 118.70 and 118.66 (CH, C2), 114.2 (CH_2, C9), 113.77 and 113.74 (CH, C8′), 79.6 (C, C4′), 73.0 (CH_2, C11′), 72.79 and 72.76 (CH, C7), 70.32 and 70.28 (CH, C5′), 70.17 and 70.11 (CH, C2′), 69.3 (CH_2, C1′), 55.2 (CH_3, C6′), 41.7 (CH_2, C6), 25.8 (CH_3, *t*Bu), 25.64 (CH_3, C10), 20.95 and 20.94 (CH_2, C3′), 18.1 (C, *t*Bu), -4.56 and -4.90 (CH_3, TBS).

IR (neat) cm^{-1}: 3308 (w, C–H), 2930 (m, C–H), 2857 (s, C–H), 2214 (w, C≡C), 1712 (s, C=O), 1635 (m, C=C), 1612 (m, C=C), 1514 (s, C–H), 1248 (s, C–O), 1033 (m, C–O), 836 (s, C–Si), 776 (s, C–Si).

HRMS (ESI) *m/z*: found 499.2868, calcd for $C_{29}H_{43}O_5Si$ $[M+H]^+$ 499.2874 (Δ = 1.2 ppm).

References:

(1) F. A. Luzzio and J. Chen, *J. Org. Chem.*, 2008, **73**, 5621.

(2) C. R. Smith, *Synlett*, 2009, **9**, 1522.

(3) R. K. Dieter, L. A. Silks III, J. R. Fishpaugh and M. E. Kastner, *J. Am. Chem. Soc.*, 1985, **107**, 4679.

(4) B. M. Trost, M. R. Machacek and B. D. Faulk, *J. Am. Chem. Soc.*, 2006, **128**, 6745.

(5) K. C. Nicolaou, K. C. Fylaktakidou, H. Monenschein, Y. Li, B. Weyershausen, H. J. Mitchell, H. Wei, P. Guntupalli, D. Hepworth and K. Sugita, *J. Am. Chem. Soc.*, 2003, **125**, 15433.

(6) Y. Kiyotsuka and Y. Kobayashi, *J. Org. Chem.*, 2009, **74**, 7489.

(7) R. J. Maguire, S. P. Munt and E. J. Thomas, *J. Chem. Soc. Perkin Trans. 1*, 1998, 2853.

(8) E. Roulland, C. Monneret, J. C. Florent, C. Bennejean, P. Renard and S. Leonce, *J. Org .Chem.*, 2002, **67**, 4399.

(9) G. L. Larson, F. Quiroz and J. Suárez, *Synth. Commun.* 1983, **13**, 833.

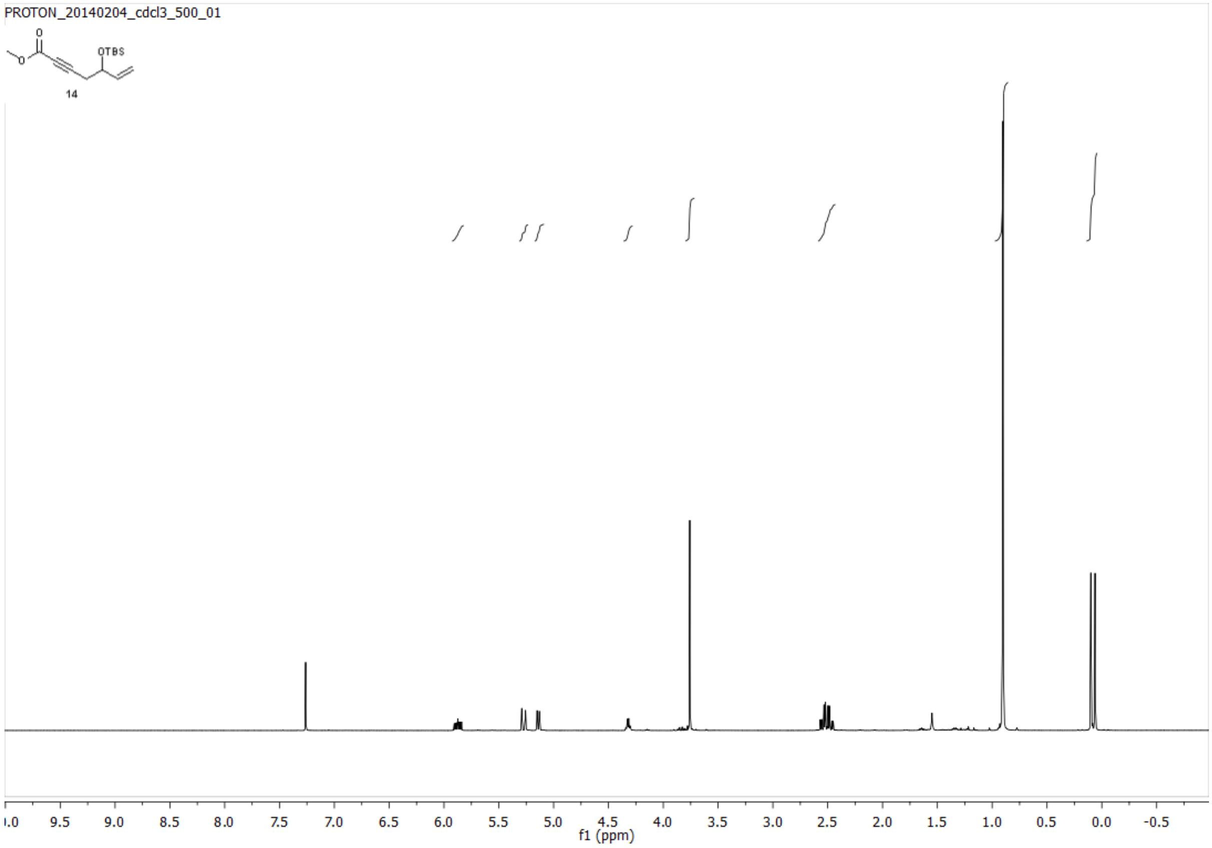
PROTON_20140204_cdcl3_500_01
OTBS
14
f1 (ppm)

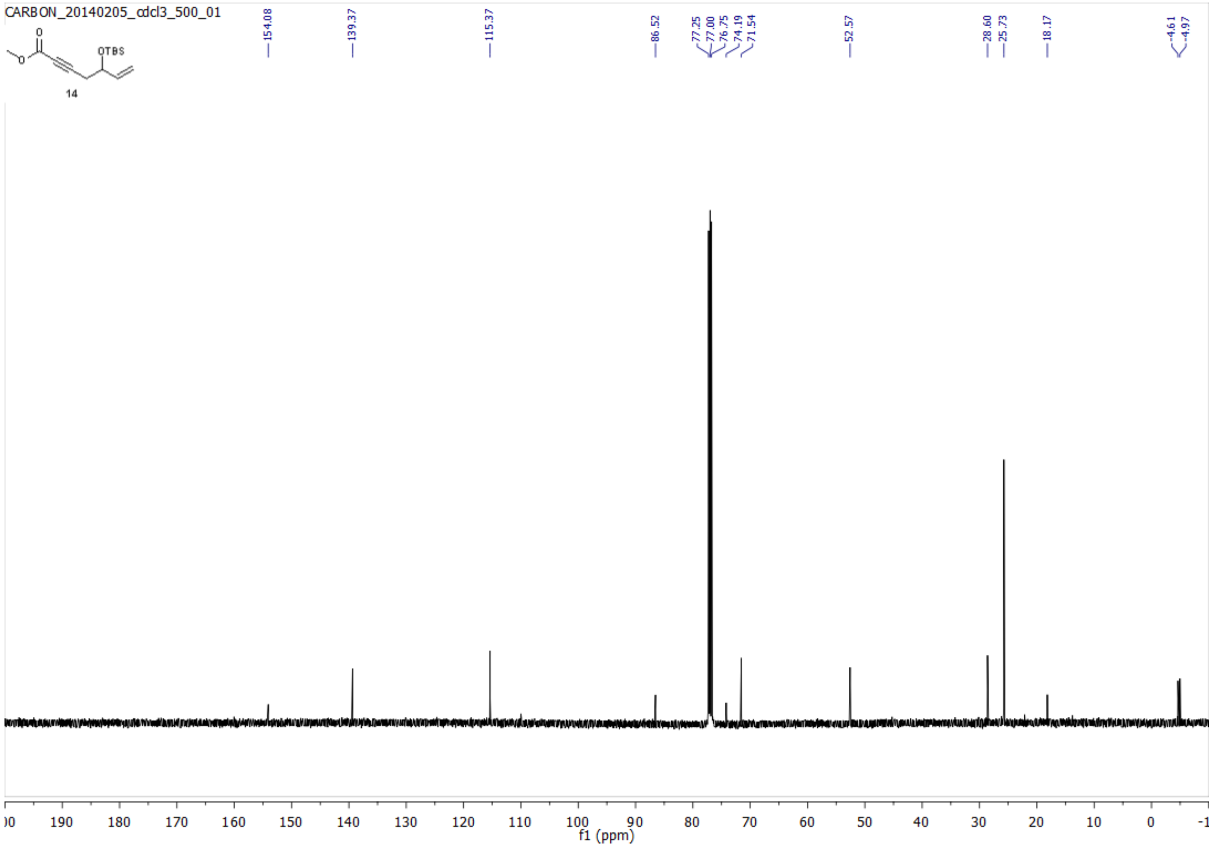
CARBON_20140205_cdcl3_500_01
OTBS
14
154.08
139.37
115.37
86.52
77.25
77.00
76.75
74.19
71.54
52.57
28.60
25.73
18.17
-4.61
-4.97
f1 (ppm)

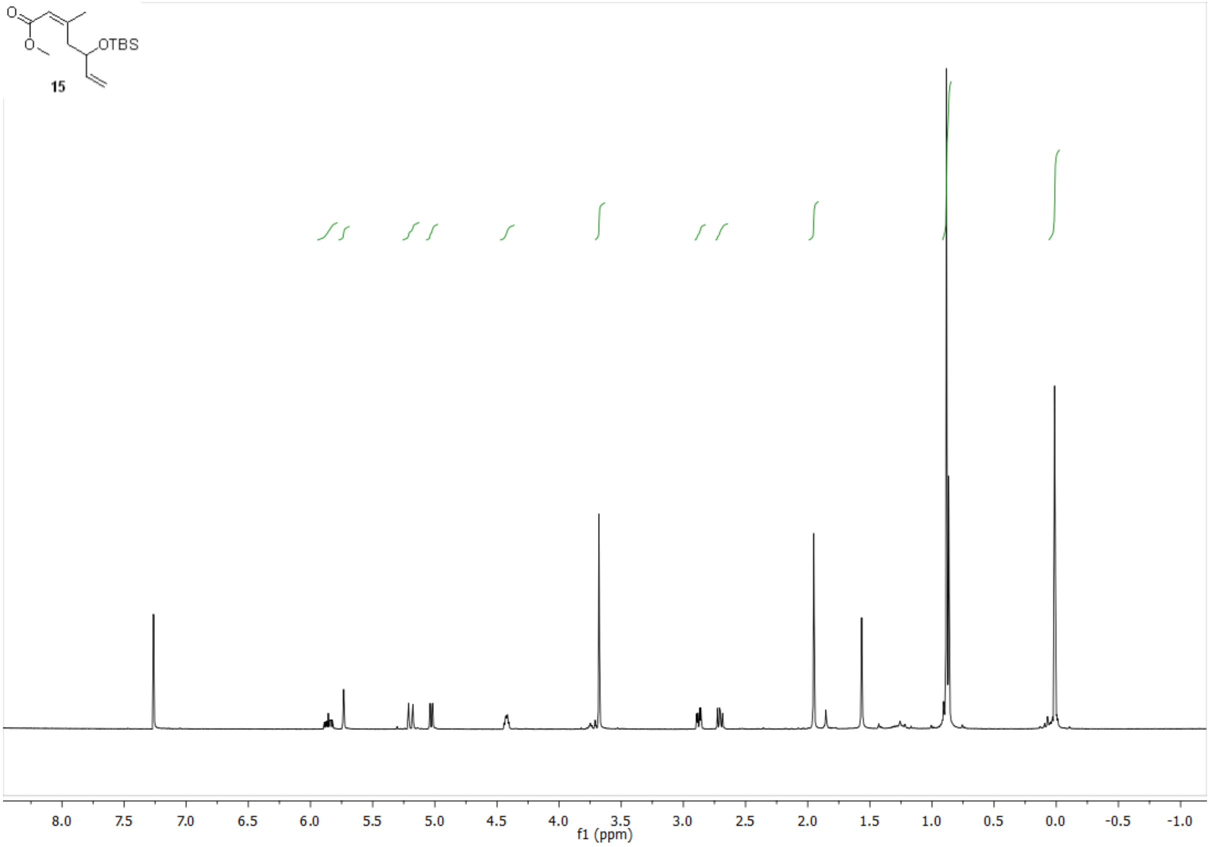
OTBS
15
8.0 7.5 7.0 6.5 6.0 5.5 5.0 4.5 4.0 3.5 3.0 2.5 2.0 1.5 1.0 0.5 0.0 -0.5 -1.0
f1 (ppm)

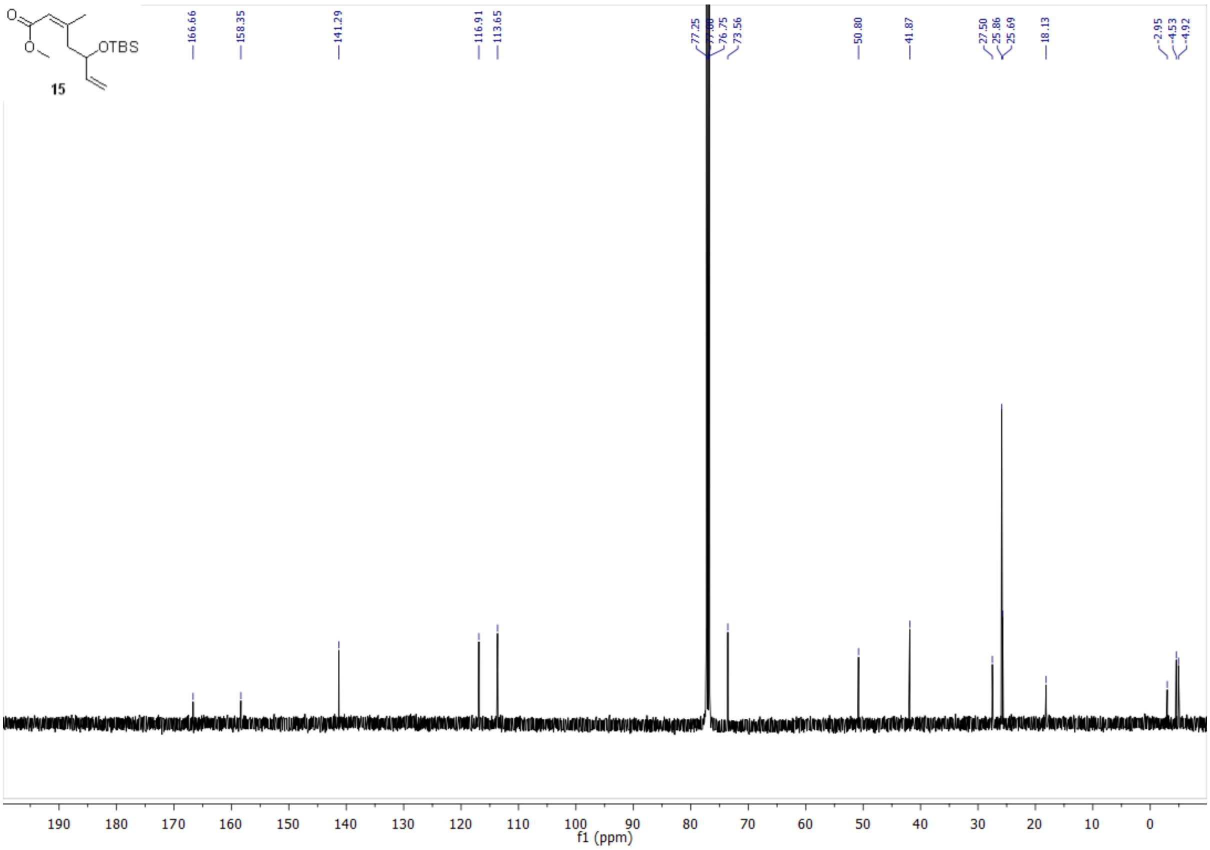
OTBS
15
166.66
158.35
141.29
116.91
113.65
77.25
76.75
73.56
50.80
41.87
27.50
25.86
25.69
18.13
-2.95
-4.53
-4.92
190 180 170 160 150 140 130 120 110 100 90 80 70 60 50 40 30 20 10 0
f1 (ppm)

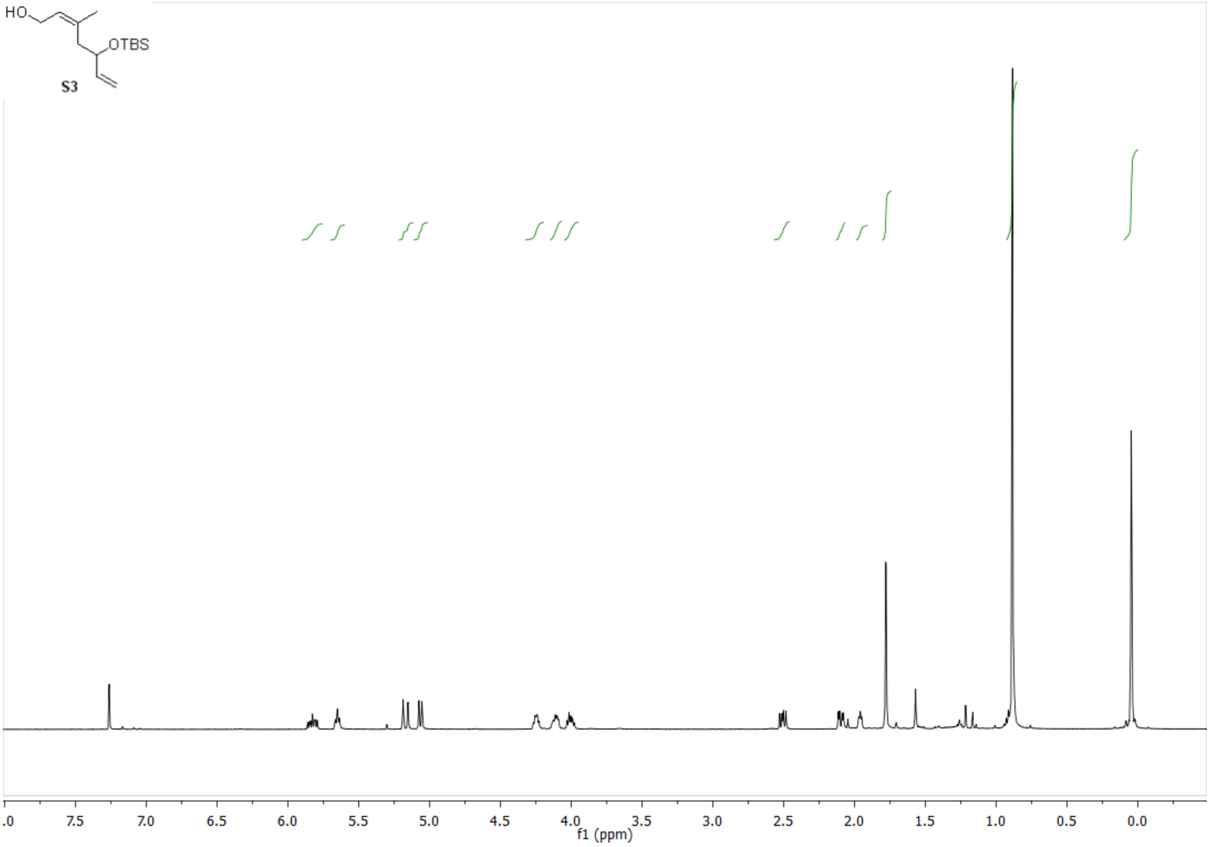
HO
OTBS
S3
f1 (ppm)

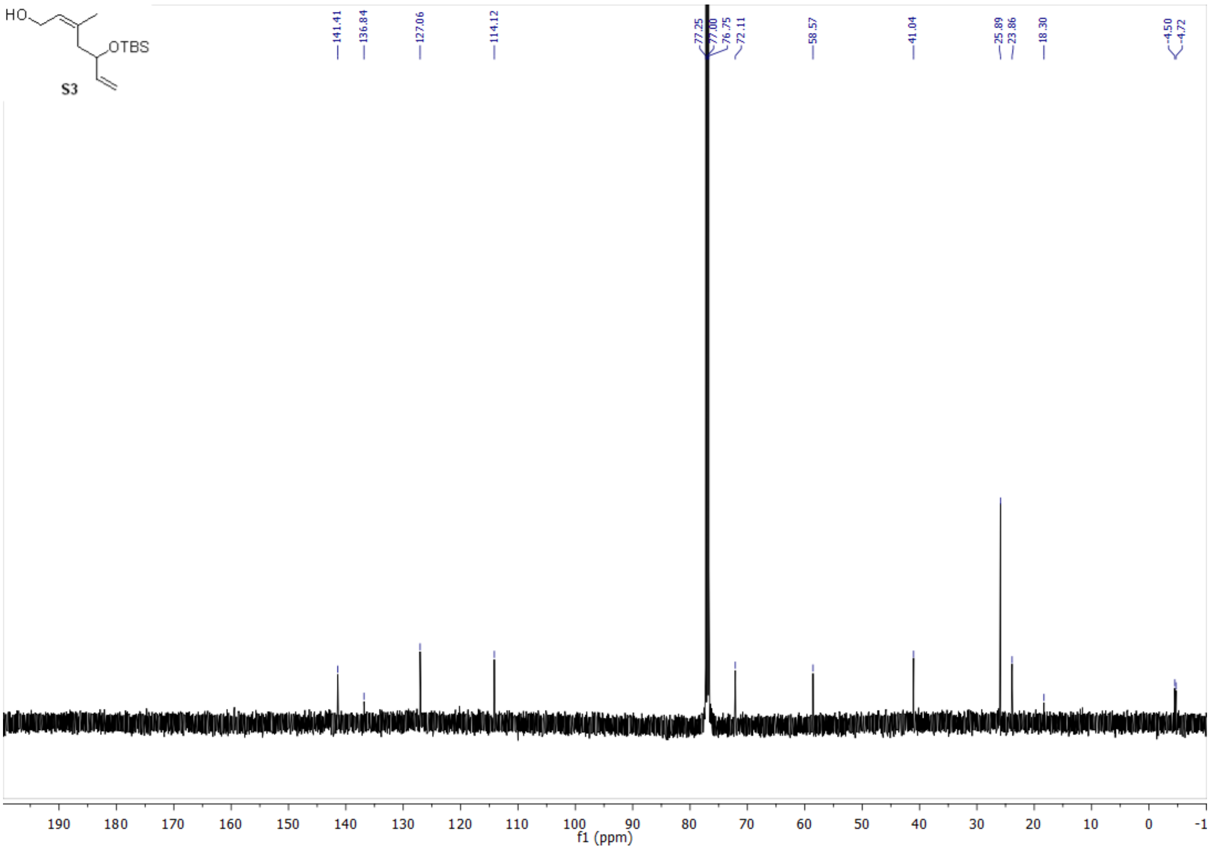
HO
OTBS
S3
141.41
136.84
127.06
114.12
77.25
77.00
76.75
72.11
58.57
41.04
25.89
23.86
18.30
-4.50
-4.72
f1 (ppm)

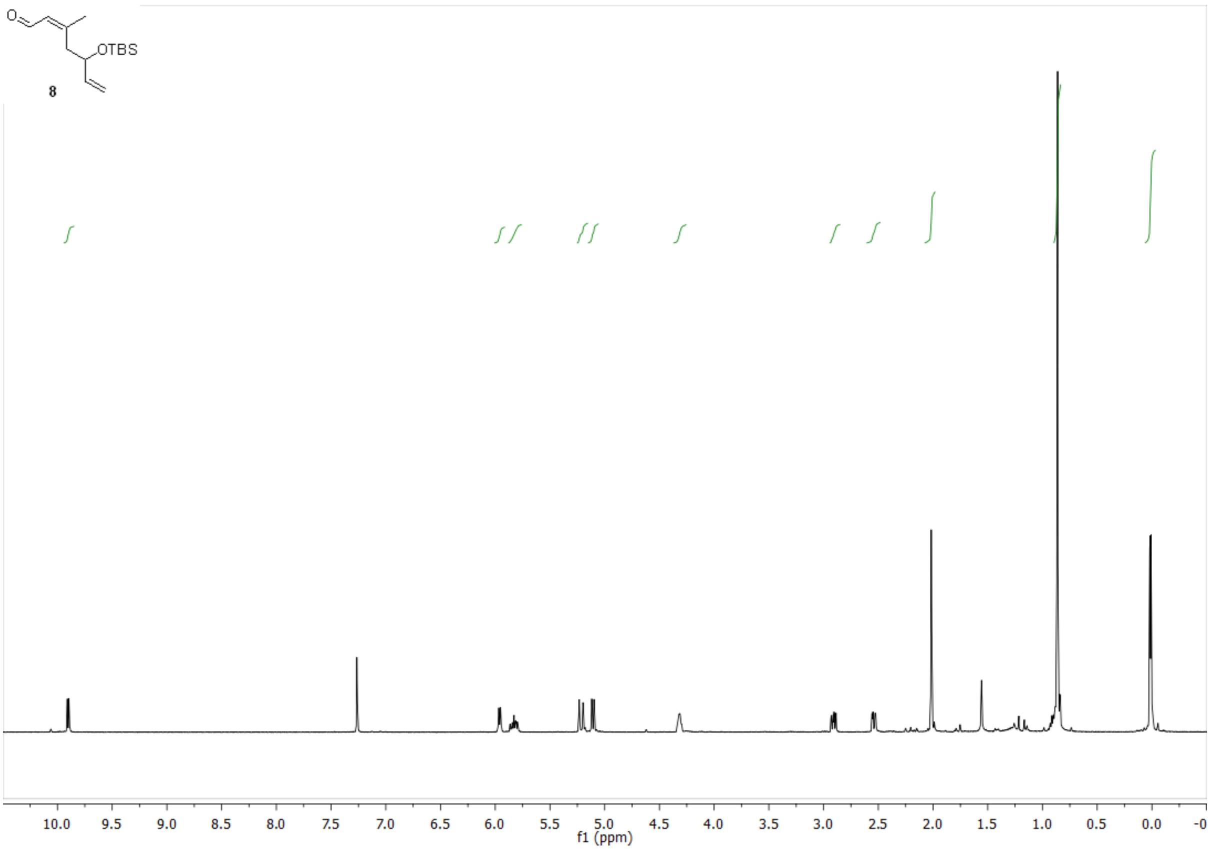

OTBS
8
10.0
9.5
9.0
8.5
8.0
7.5
7.0
6.5
6.0
5.5
5.0
4.5
4.0
3.5
3.0
2.5
2.0
1.5
1.0
0.5
0.0
f1 (ppm)

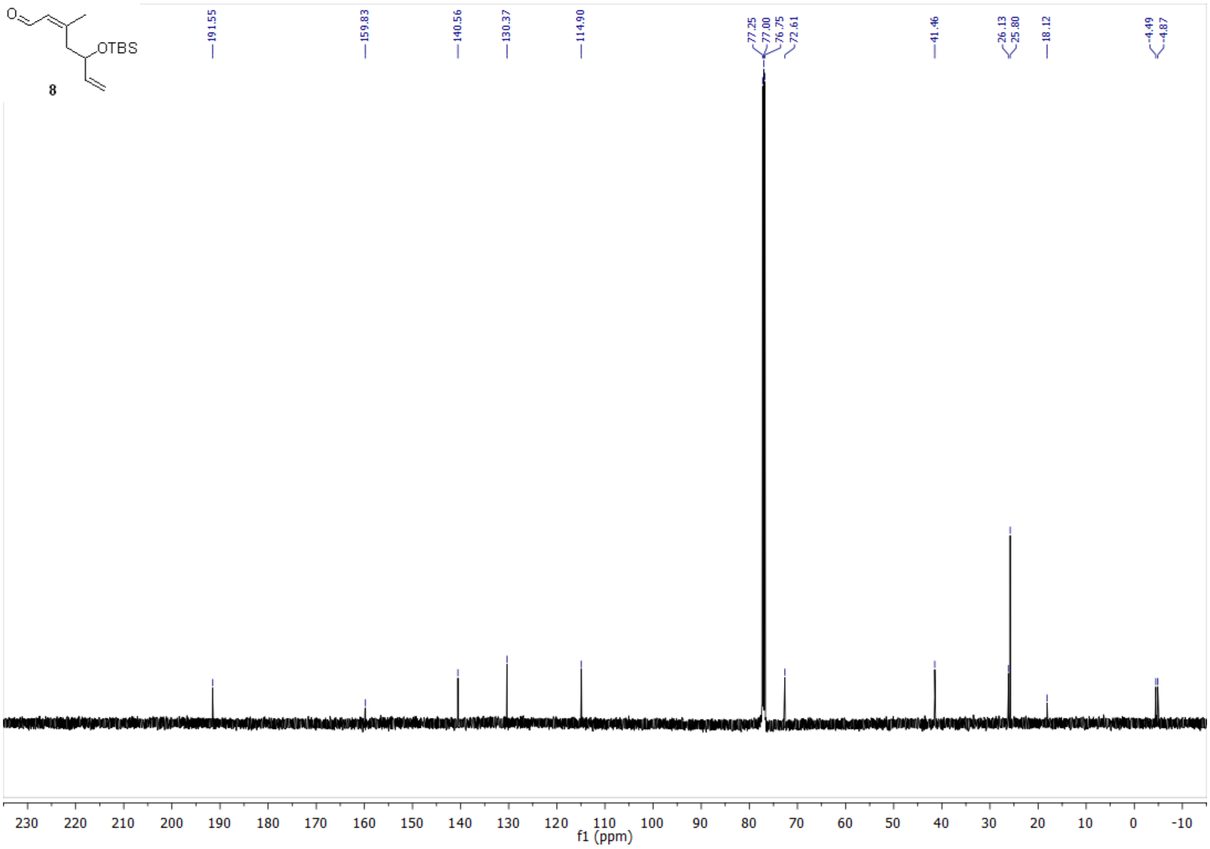

OTBS
8
191.55
159.83
140.56
130.37
114.90
77.25
77.00
76.75
72.61
41.46
26.13
25.80
18.12
-4.49
-4.87
230
220
210
200
190
180
170
160
150
140
130
120
110
100
90
80
70
60
50
40
30
20
10
0
-10
f1 (ppm)

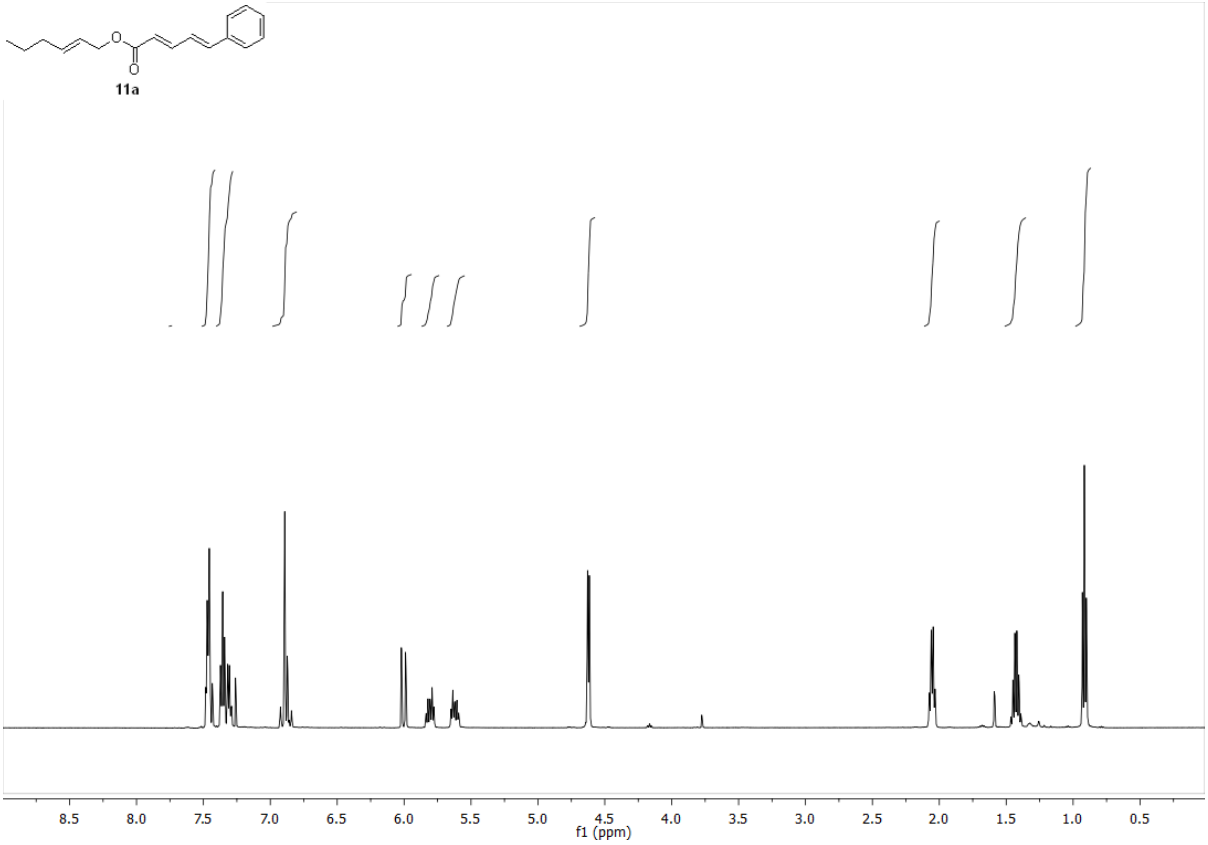
11a
8.5 8.0 7.5 7.0 6.5 6.0 5.5 5.0 4.5 4.0 3.5 3.0 2.5 2.0 1.5 1.0 0.5
f1 (ppm)

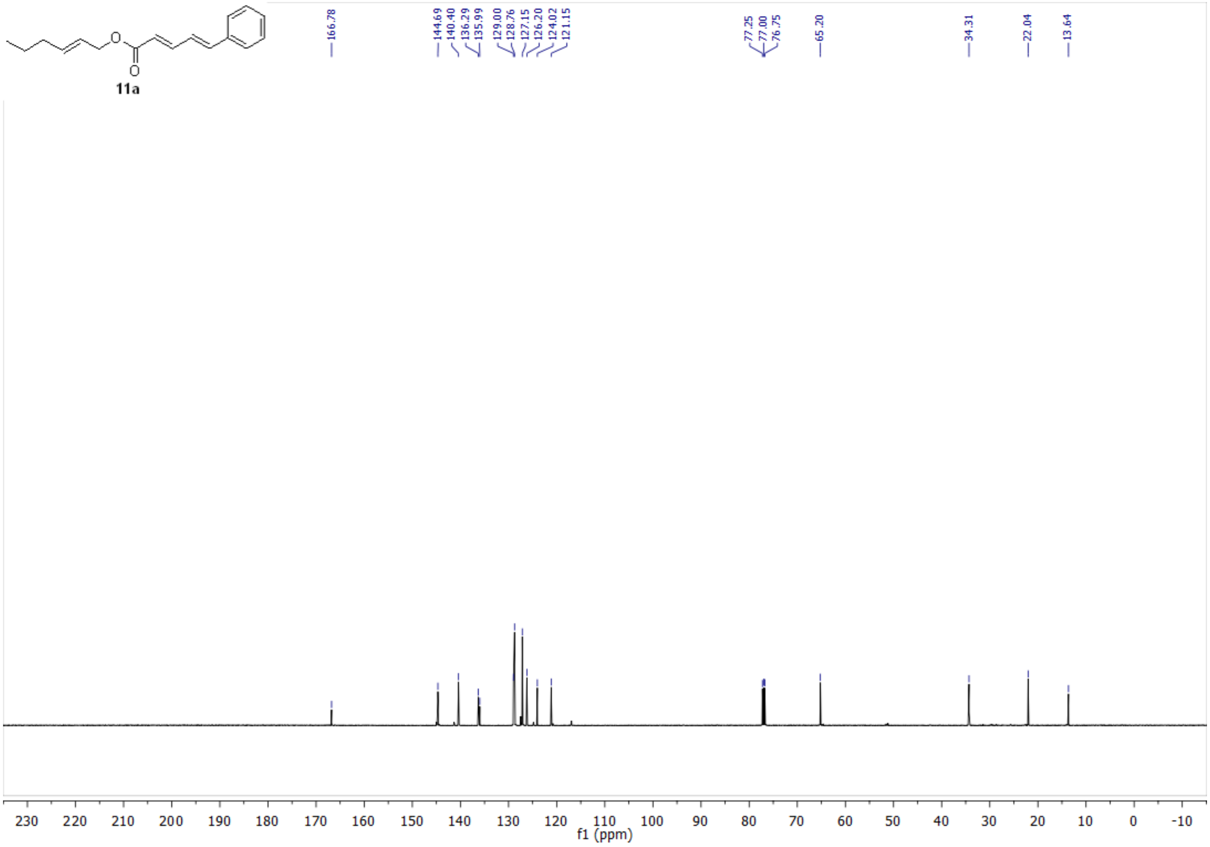
11a
166.78
144.69
140.40
136.29
135.99
129.00
128.76
127.15
126.20
124.02
121.15
77.25
77.00
76.75
65.20
34.31
22.04
13.64
230 220 210 200 190 180 170 160 150 140 130 120 110 100 90 80 70 60 50 40 30 20 10 0 -10
f1 (ppm)

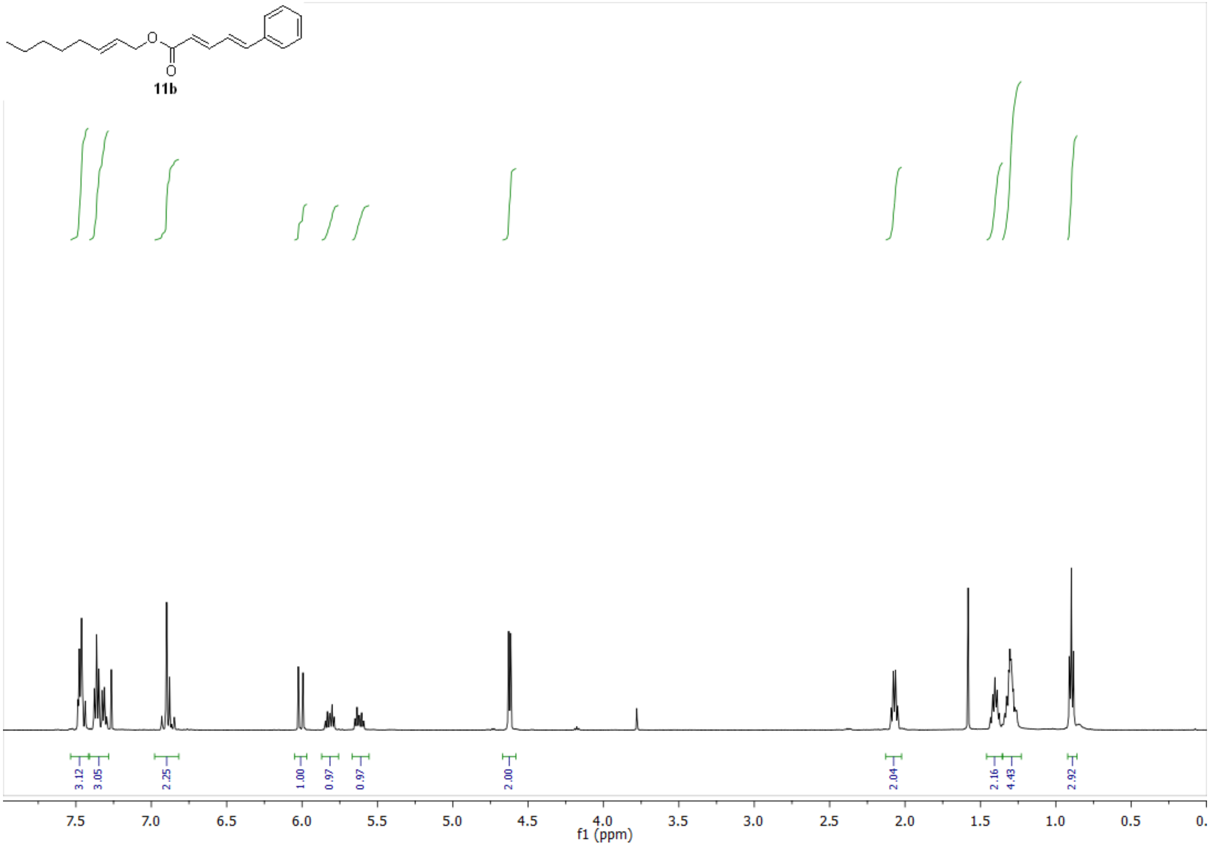

O
O
11b
3.12
3.05
2.25
1.00
0.97
0.97
2.00
2.04
2.16
4.43
2.92
f1 (ppm)

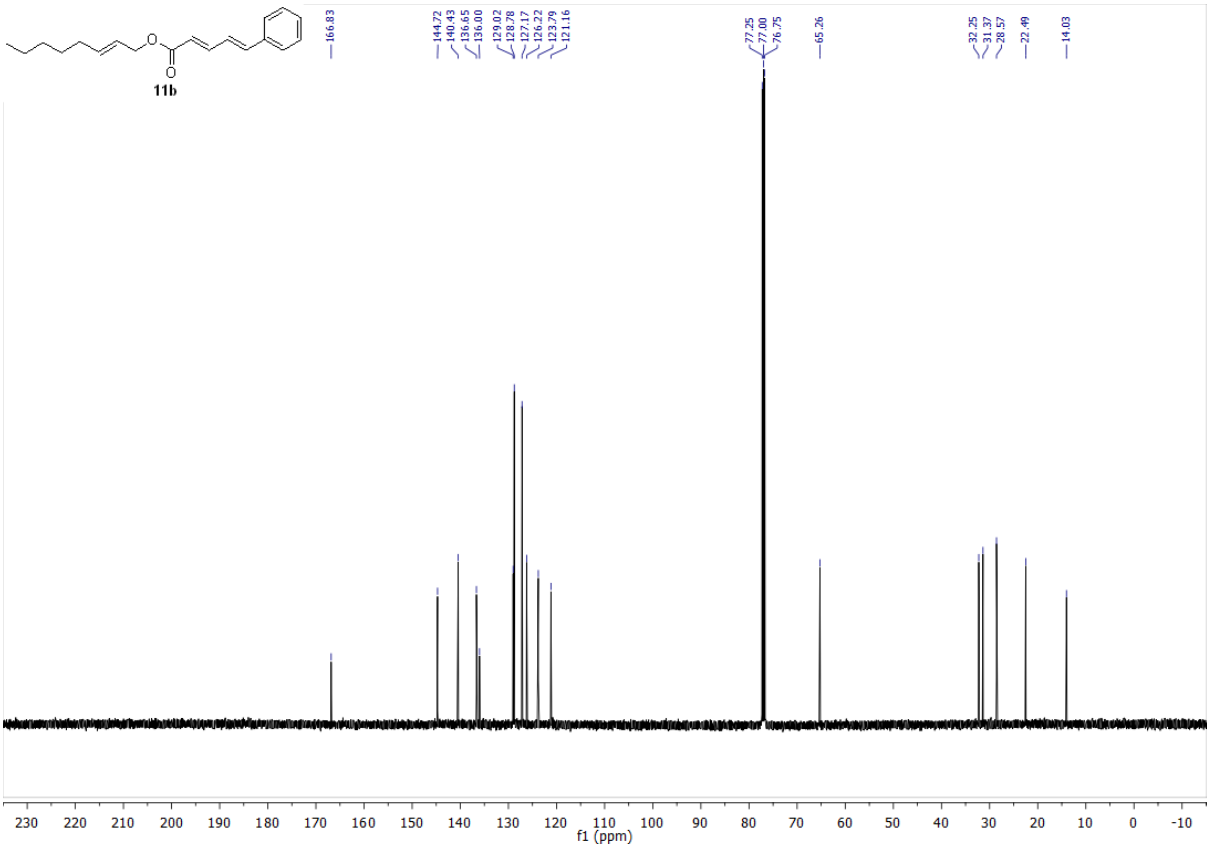

O
O
11b
166.83
144.72
140.43
136.65
136.00
129.02
128.78
127.17
126.22
123.79
121.16
77.25
77.00
76.75
65.26
32.25
31.37
28.57
22.49
14.03
f1 (ppm)

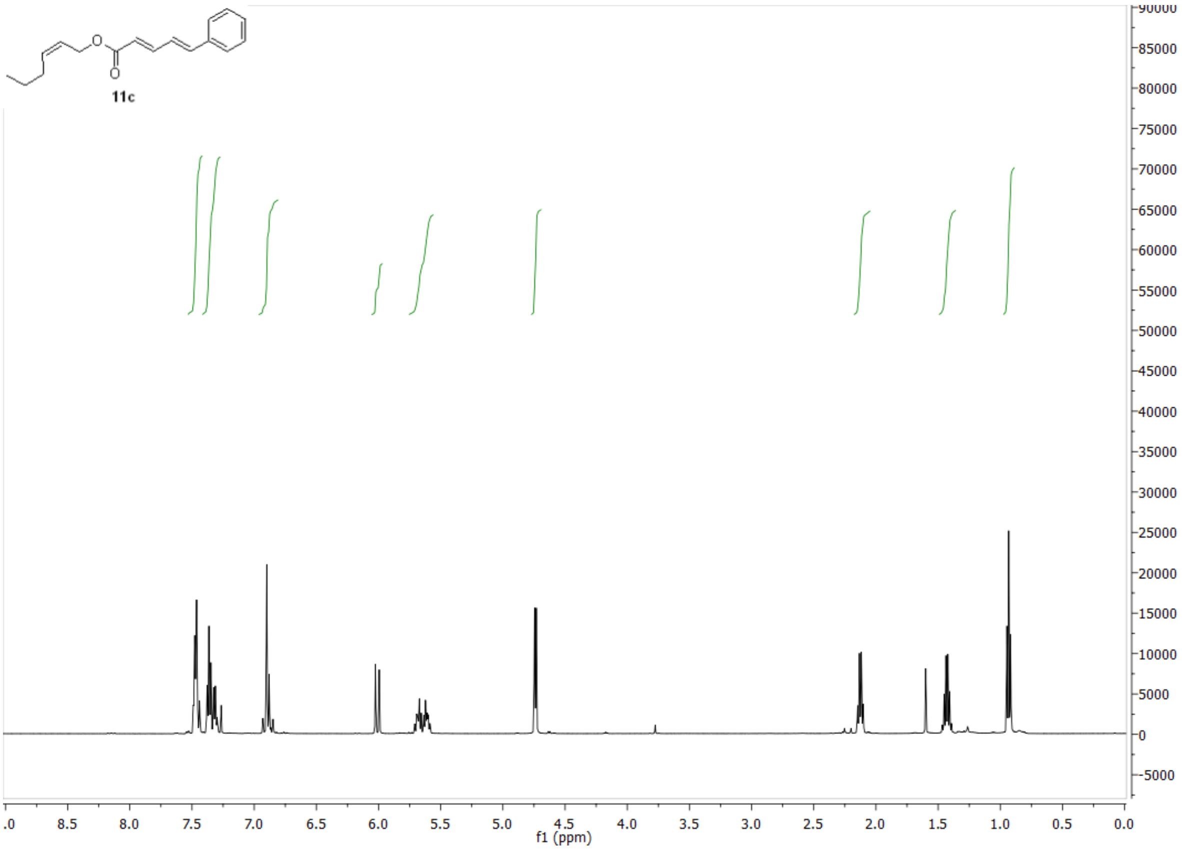

11c
f1 (ppm)

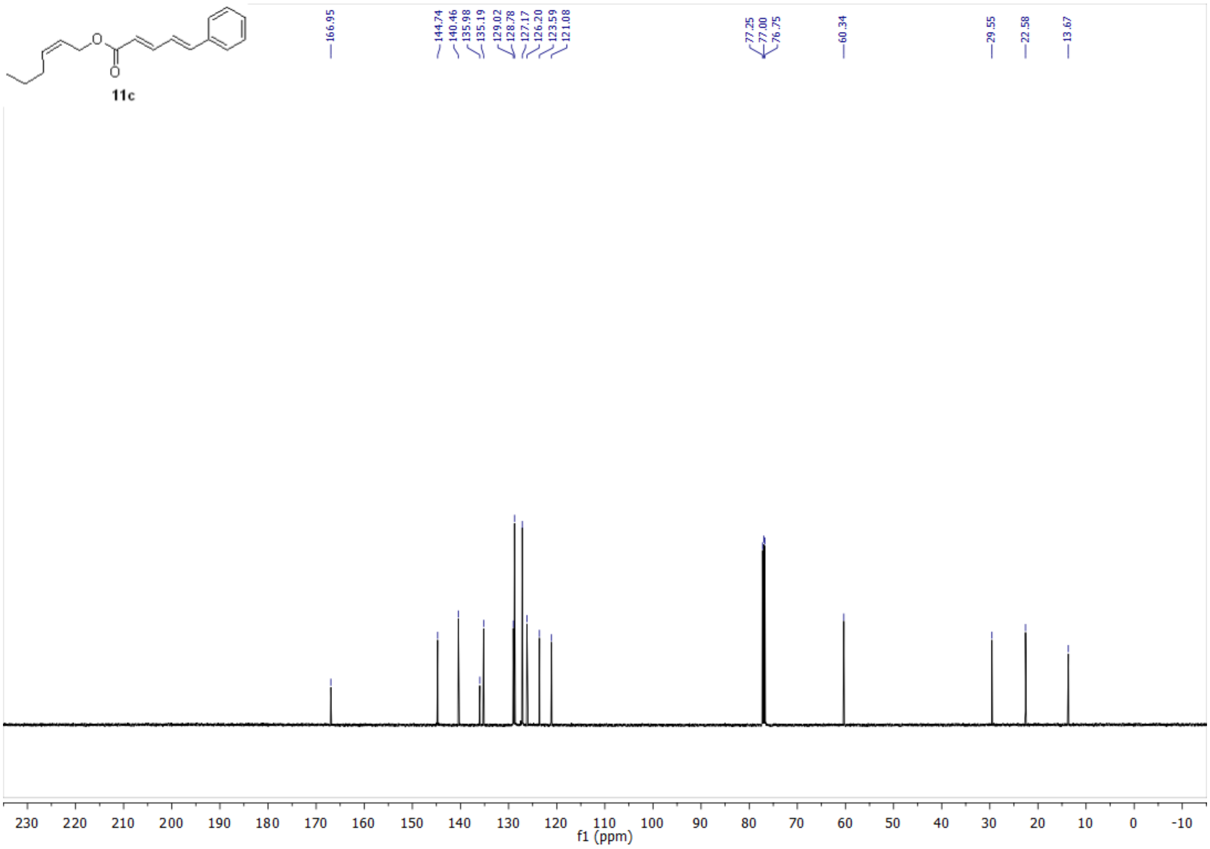

11c
166.95
144.74
140.46
135.98
135.19
129.02
128.78
127.17
126.20
123.59
121.08
77.25
77.00
76.75
60.34
29.55
22.58
13.67
f1 (ppm)

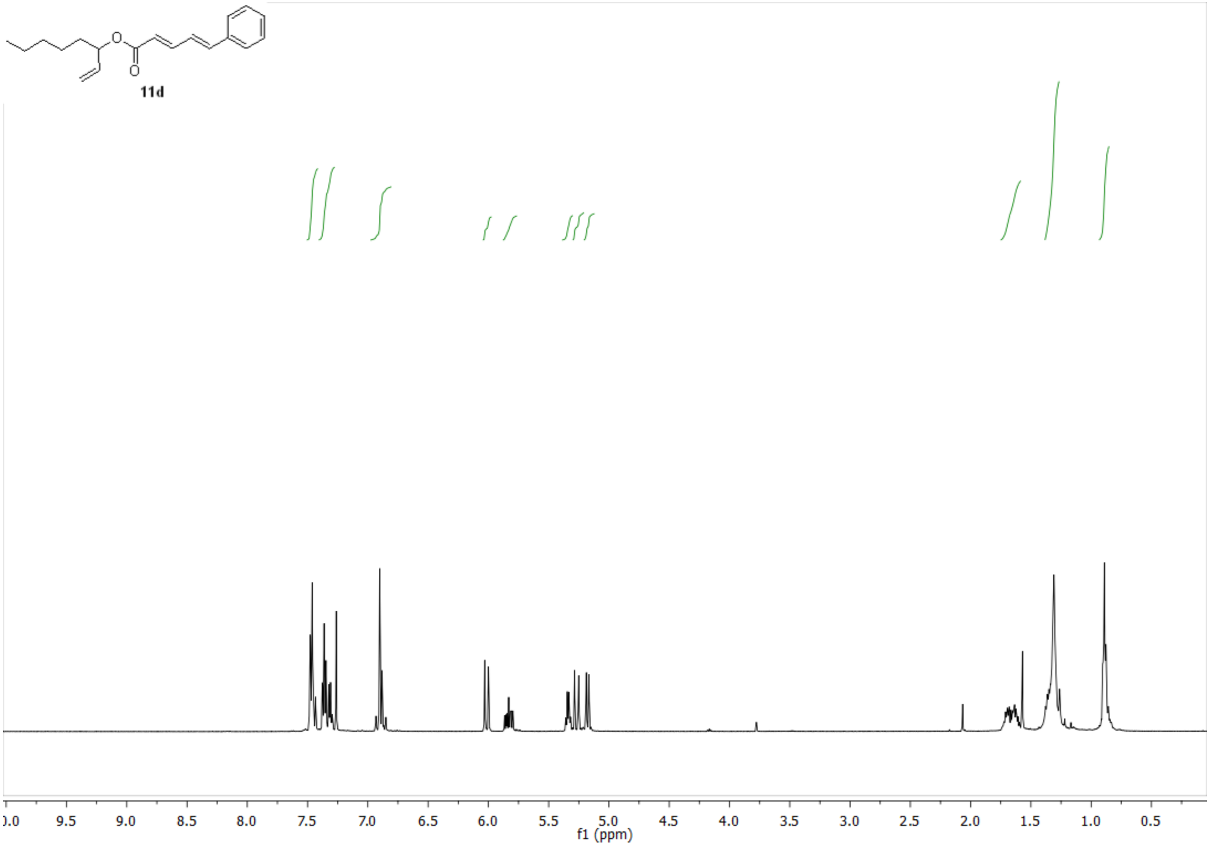
11d
f1 (ppm)

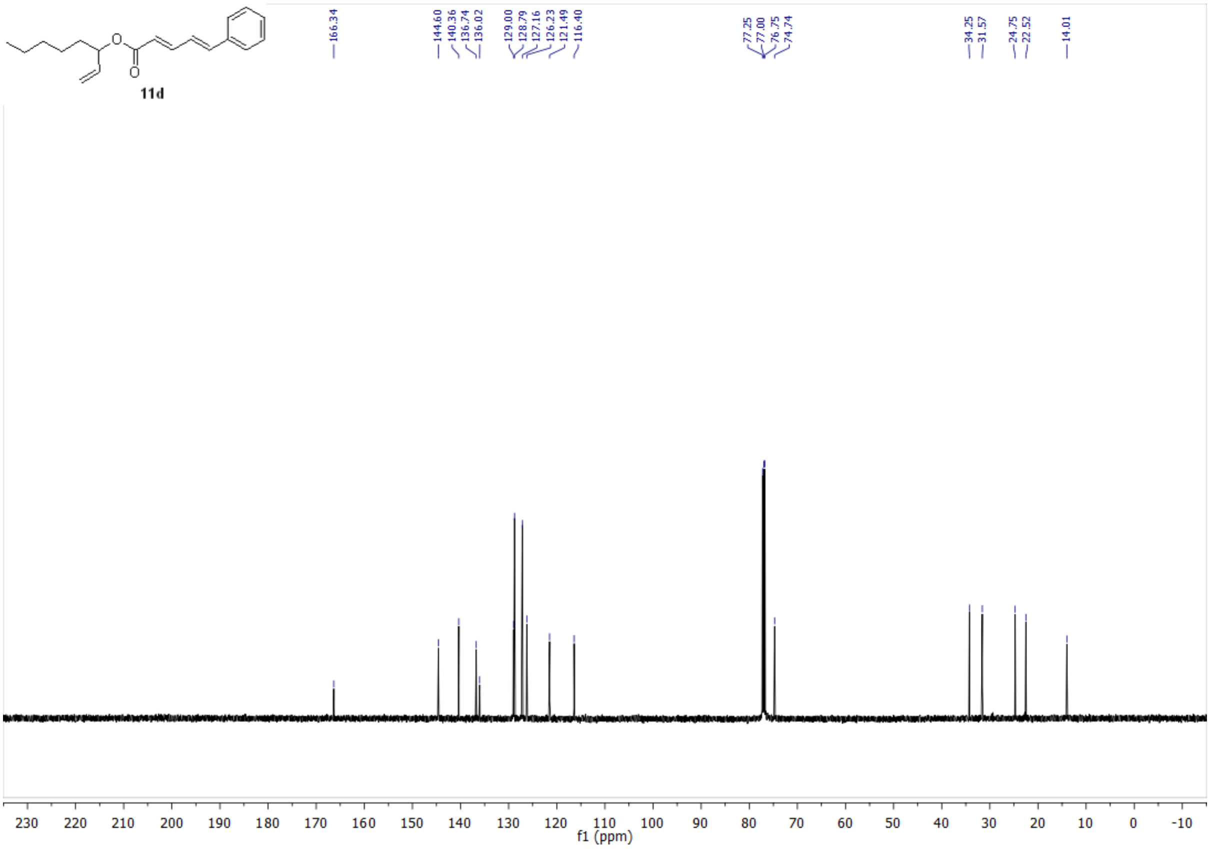
11d
166.34
144.60
140.36
136.74
136.02
129.00
128.79
127.16
126.23
121.49
116.40
77.25
77.00
76.75
74.74
34.25
31.57
24.75
22.52
14.01
f1 (ppm)

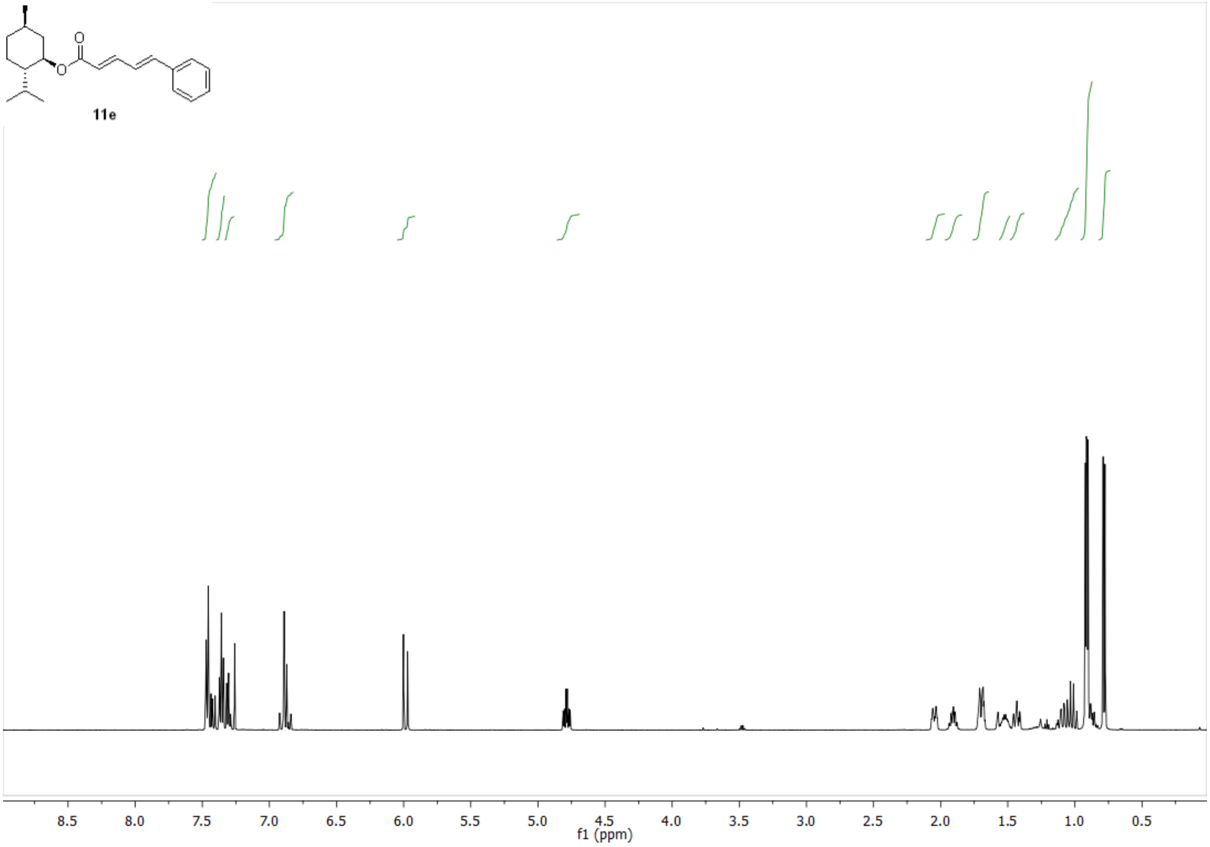
11e
f1 (ppm)

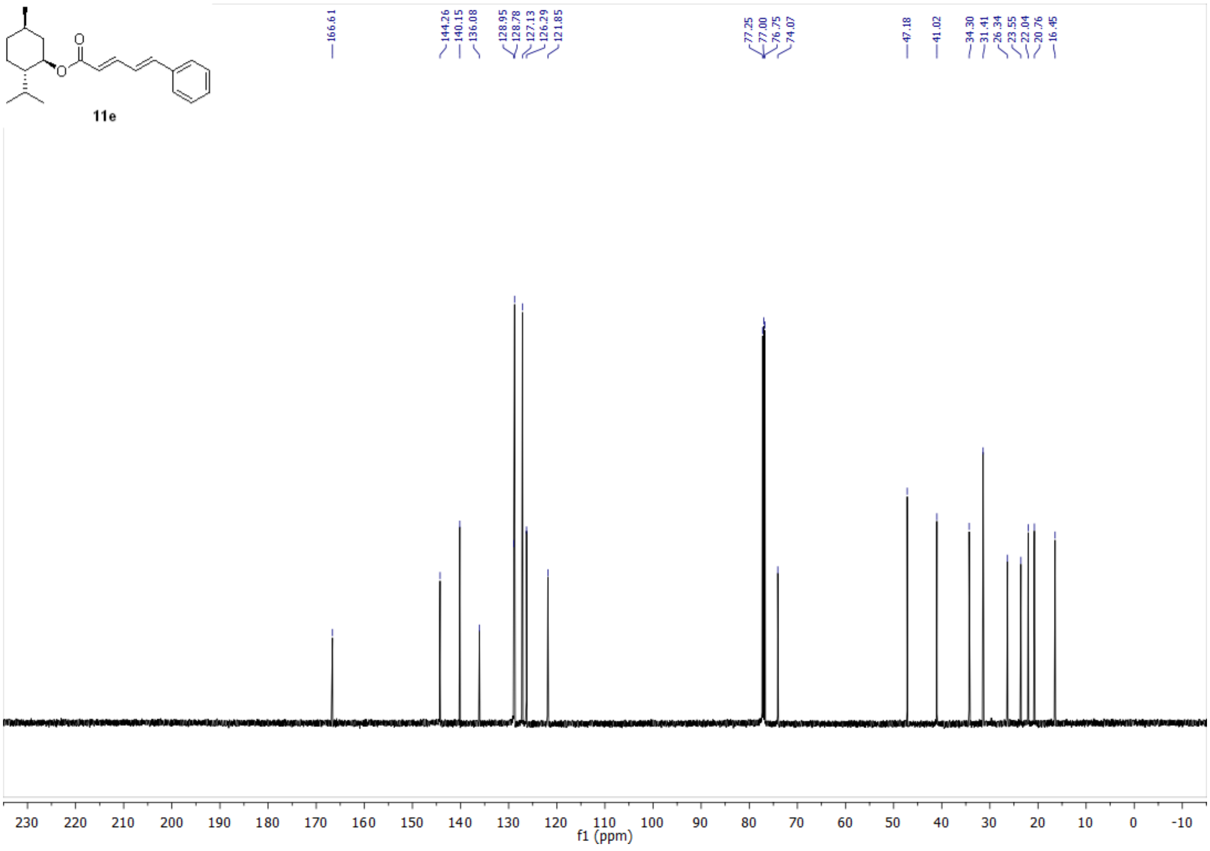
11e
166.61
144.26
140.15
136.08
128.95
128.78
127.13
126.29
121.85
77.25
77.00
76.75
74.07
47.18
41.02
34.30
31.41
26.34
23.55
22.04
20.76
16.45
f1 (ppm)

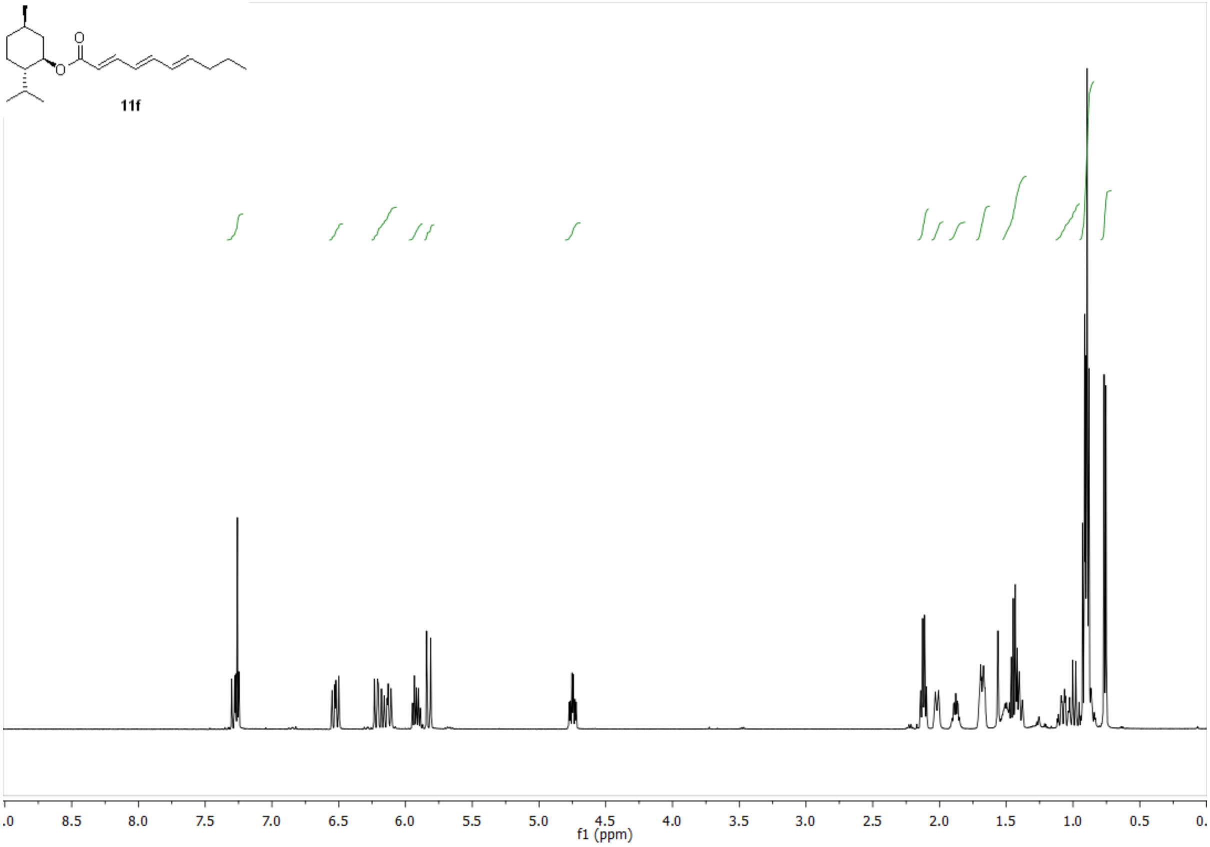
O
O
11f
9.0
8.5
8.0
7.5
7.0
6.5
6.0
5.5
5.0
4.5
4.0
3.5
3.0
2.5
2.0
1.5
1.0
0.5
0.0
f1 (ppm)

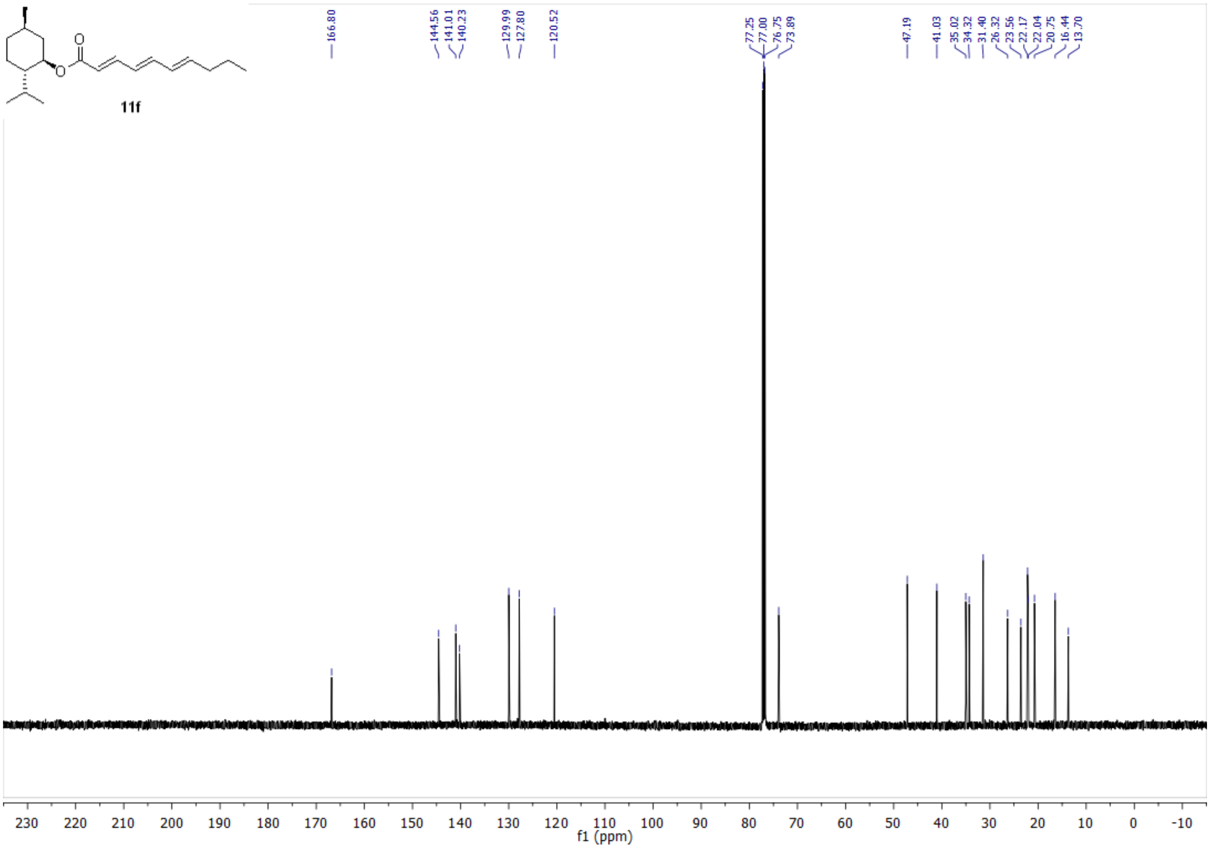
O
O
11f
166.80
144.56
141.01
140.23
129.99
127.80
120.52
77.25
77.00
76.75
73.89
47.19
41.03
35.02
34.32
31.40
26.32
23.56
22.17
22.04
20.75
16.44
13.70
230
220
210
200
190
180
170
160
150
140
130
120
110
100
90
80
70
60
50
40
30
20
10
0
-10
f1 (ppm)

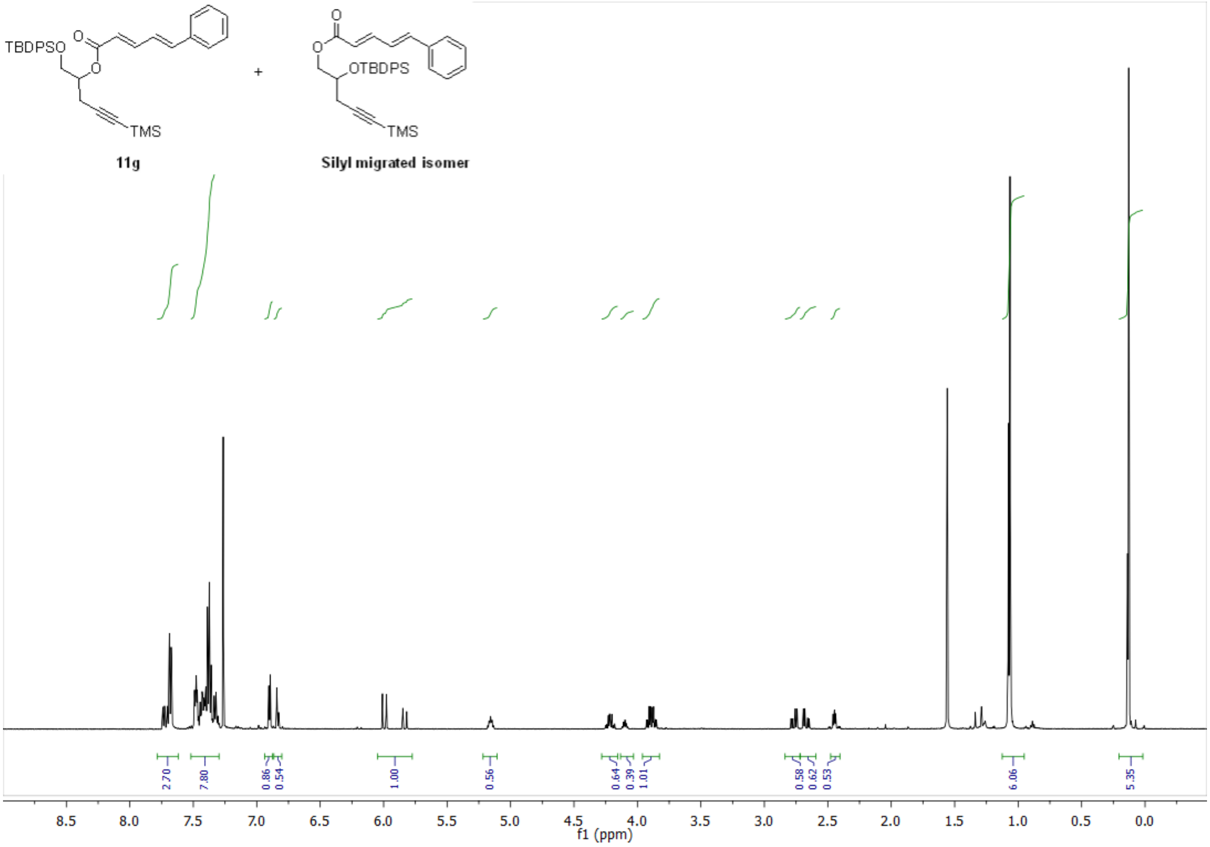
TBDPSO
O
O
TMS
11g
+
O
O
OTBDPS
TMS
Silyl migrated isomer
f1 (ppm)

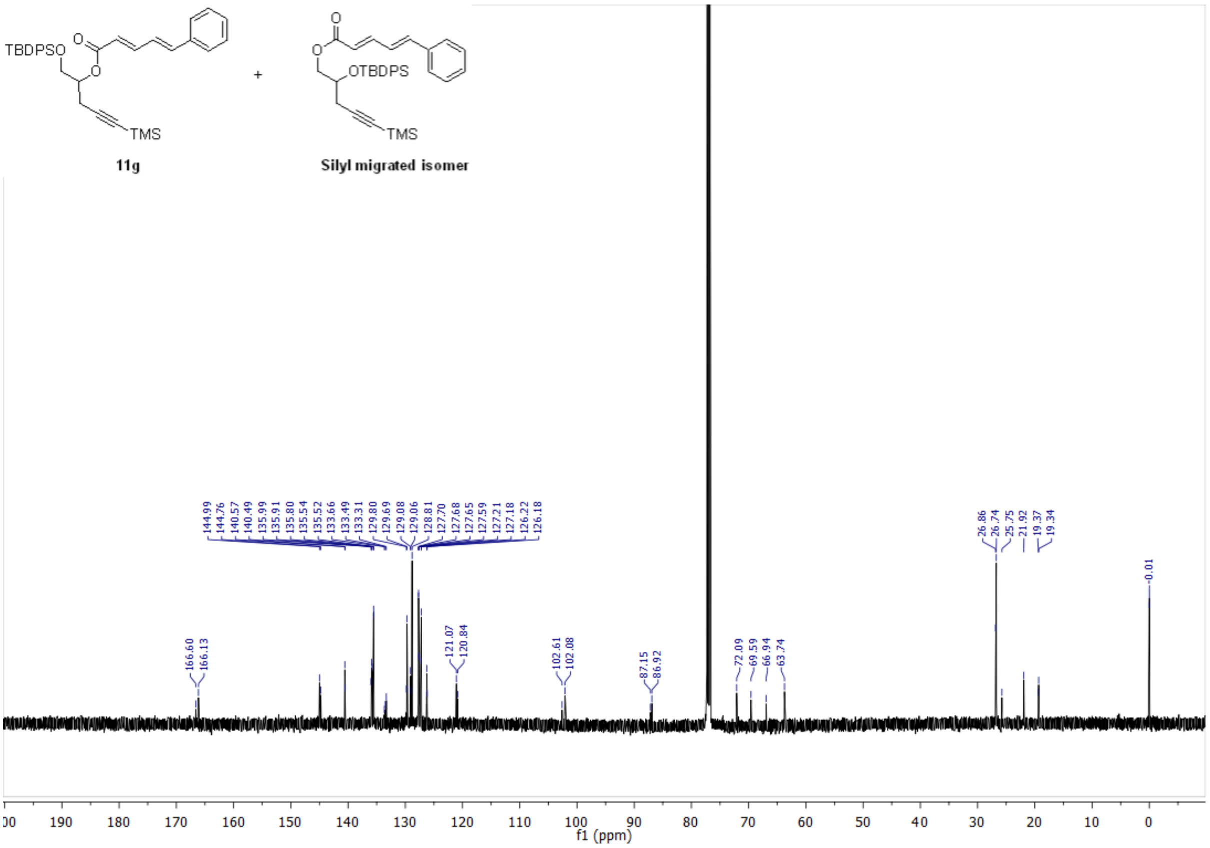
TBDPSO
O
O
TMS
11g
+
O
O
OTBDPS
TMS
Silyl migrated isomer
f1 (ppm)

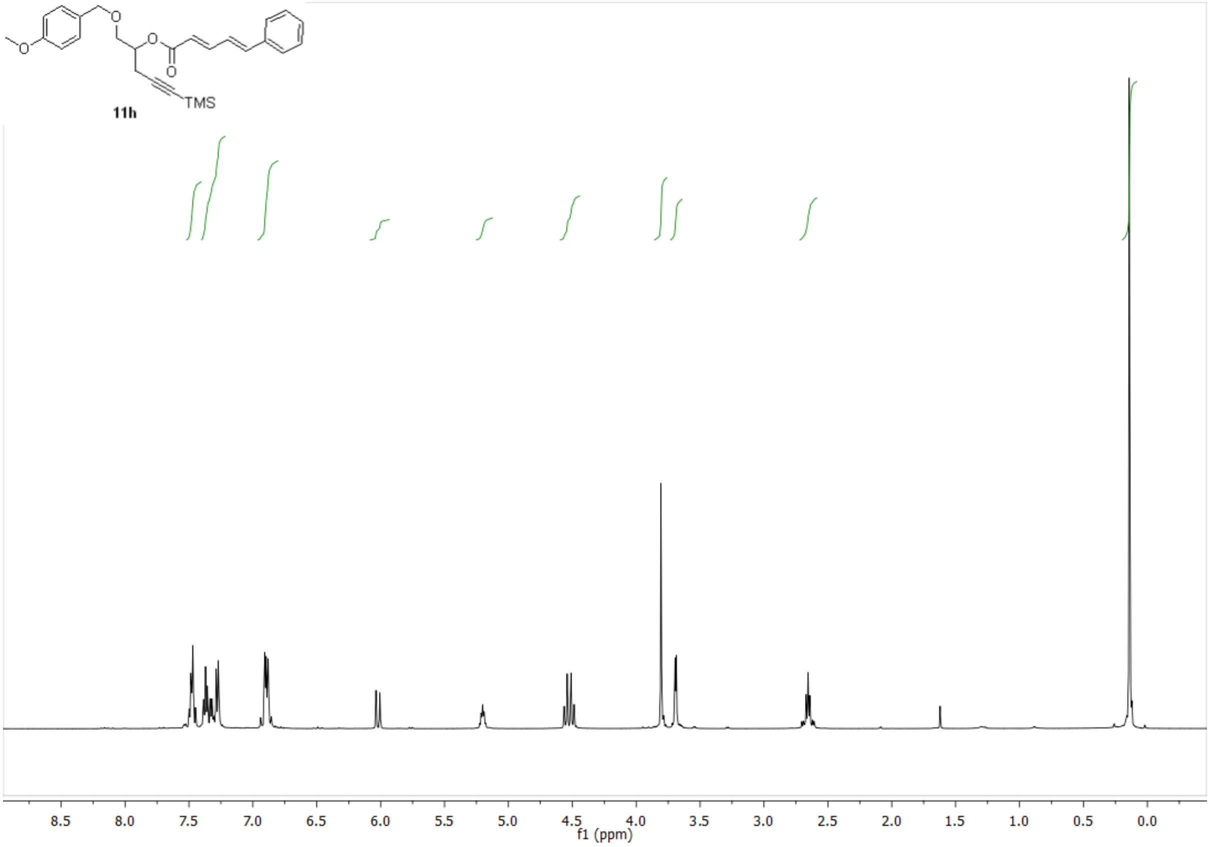
11h
TMS
8.5
8.0
7.5
7.0
6.5
6.0
5.5
5.0
4.5
4.0
3.5
3.0
2.5
2.0
1.5
1.0
0.5
0.0
f1 (ppm)

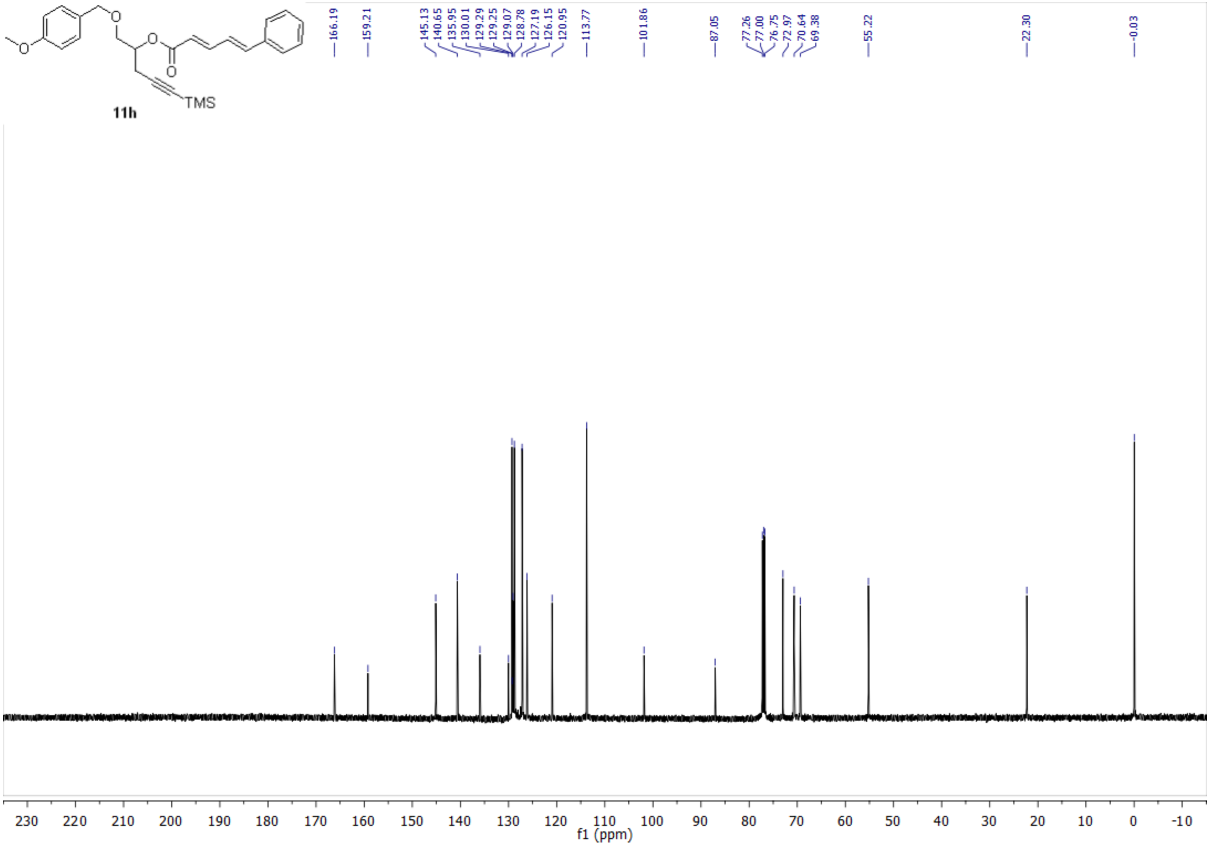
11h
TMS
166.19
159.21
145.13
140.65
135.95
130.01
129.29
129.25
129.07
128.78
127.19
126.15
120.95
113.77
101.86
87.05
77.26
77.00
76.75
72.97
70.64
69.38
55.22
22.30
-0.03
230
220
210
200
190
180
170
160
150
140
130
120
110
100
90
80
70
60
50
40
30
20
10
0
-10
f1 (ppm)

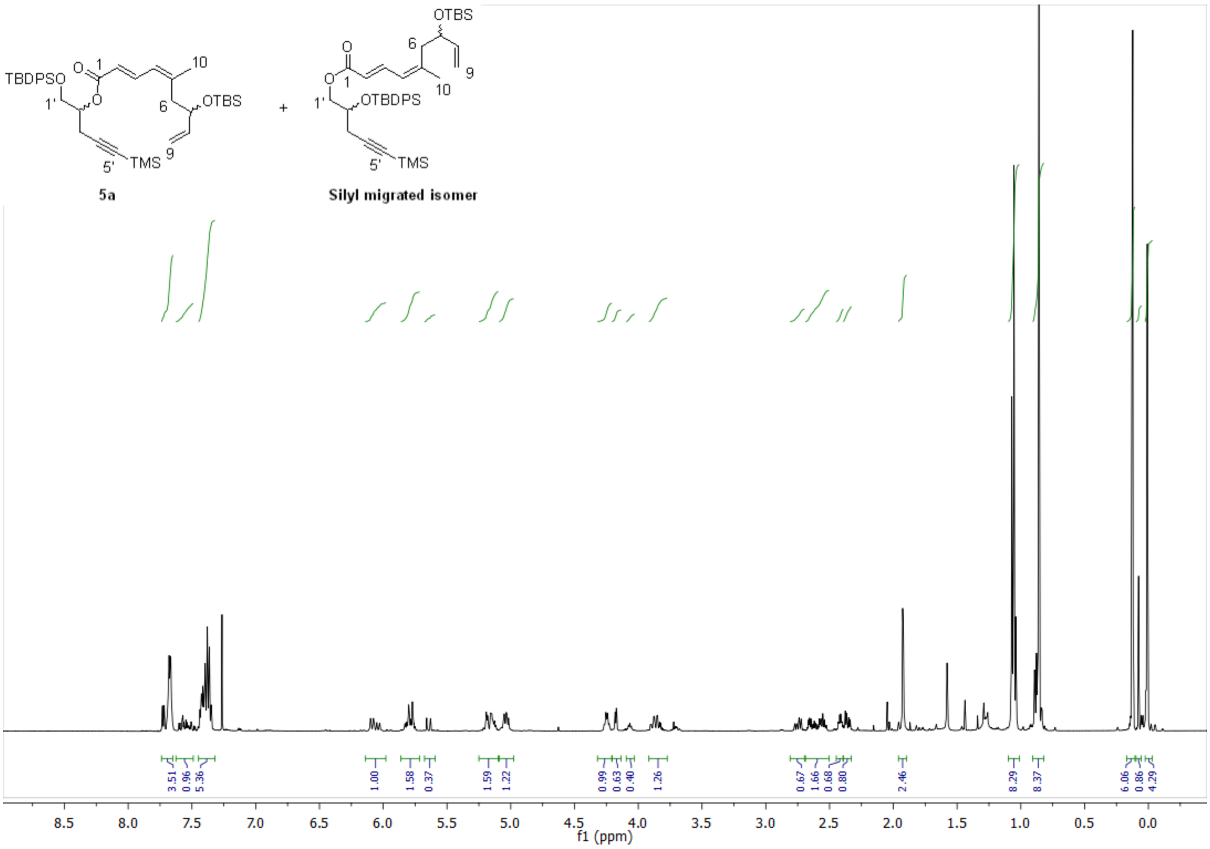
TBDPSO
O
1
10
1'
O
6
OTBS
5'
TMS
9
5a
OTBS
6
O
O
1
9
10
1'
OTBDPS
5'
TMS
Silyl migrated isomer
+
3.51
0.96
5.36
1.00
1.58
0.37
1.59
1.22
0.99
0.63
0.40
1.26
0.67
1.66
0.68
0.80
2.46
8.29
8.37
6.06
0.86
4.29
f1 (ppm)

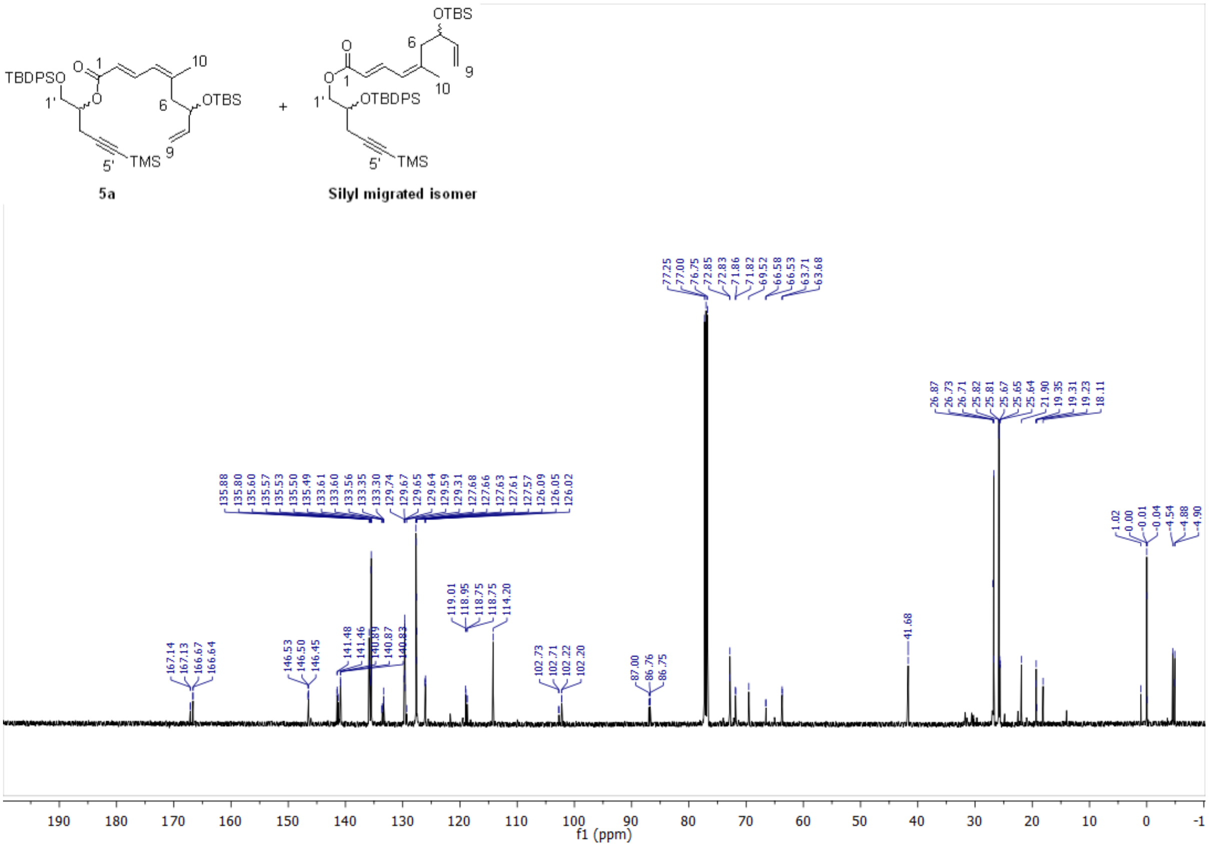
TBDPSO
O
1
10
1'
O
6
OTBS
5'
TMS
9
5a
OTBS
6
O
O
1
9
10
1'
OTBDPS
5'
TMS
Silyl migrated isomer
+
167.14
167.13
166.67
166.64
146.53
146.50
146.45
141.48
141.46
140.89
140.87
140.83
135.88
135.80
135.60
135.57
135.53
135.50
135.49
133.61
133.60
133.56
133.35
133.30
129.74
129.67
129.65
129.64
129.59
129.31
127.68
127.66
127.63
127.61
127.57
126.09
126.05
126.02
119.01
118.95
118.75
118.75
114.20
102.73
102.71
102.22
102.20
87.00
86.76
86.75
77.25
77.00
76.75
72.85
72.83
71.86
71.82
69.52
66.58
66.53
63.71
63.68
41.68
26.87
26.73
26.71
25.82
25.81
25.67
25.65
25.64
21.90
19.35
19.31
19.23
18.11
1.02
0.00
-0.01
-0.04
-4.54
-4.88
-4.90
f1 (ppm)

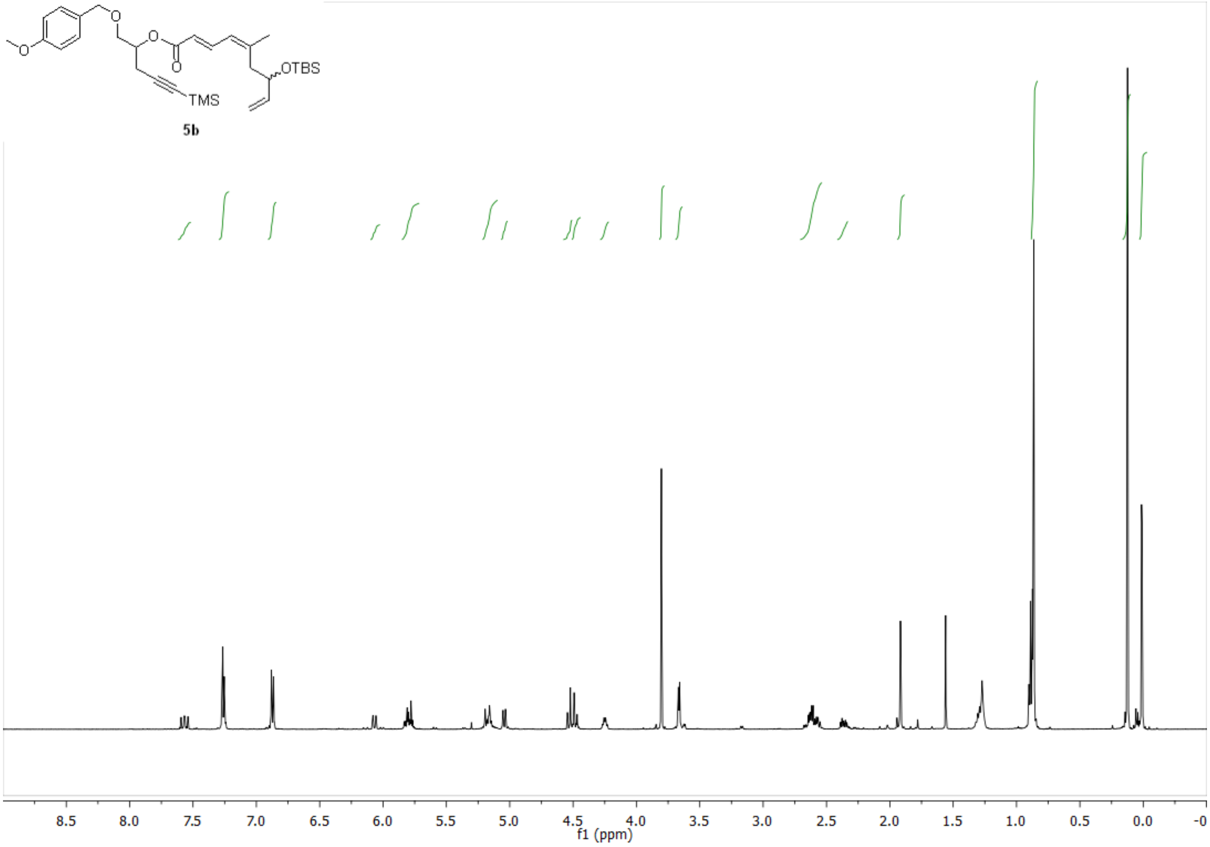
OTBS
TMS
5b
8.5 8.0 7.5 7.0 6.5 6.0 5.5 5.0 4.5 4.0 3.5 3.0 2.5 2.0 1.5 1.0 0.5 0.0
f1 (ppm)

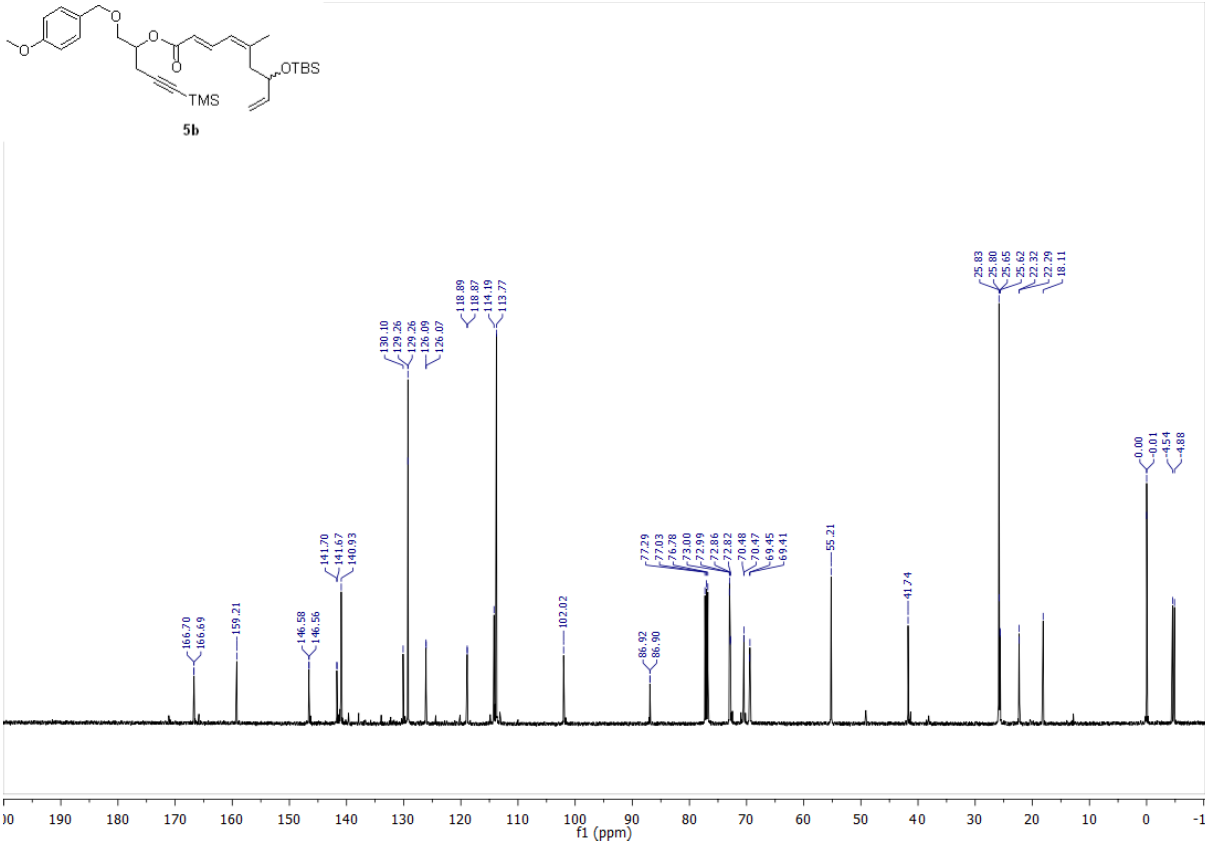
OTBS
TMS
5b
166.70
166.69
159.21
146.58
146.56
141.70
141.67
140.93
130.10
129.26
129.26
126.09
126.07
118.89
118.87
114.19
113.77
102.02
86.92
86.90
77.29
77.03
76.78
73.00
72.99
72.86
72.82
70.48
70.47
69.45
69.41
55.21
41.74
25.83
25.80
25.65
25.62
22.32
22.29
18.11
0.00
-0.01
-4.54
-4.88
190 180 170 160 150 140 130 120 110 100 90 80 70 60 50 40 30 20 10 0
f1 (ppm)

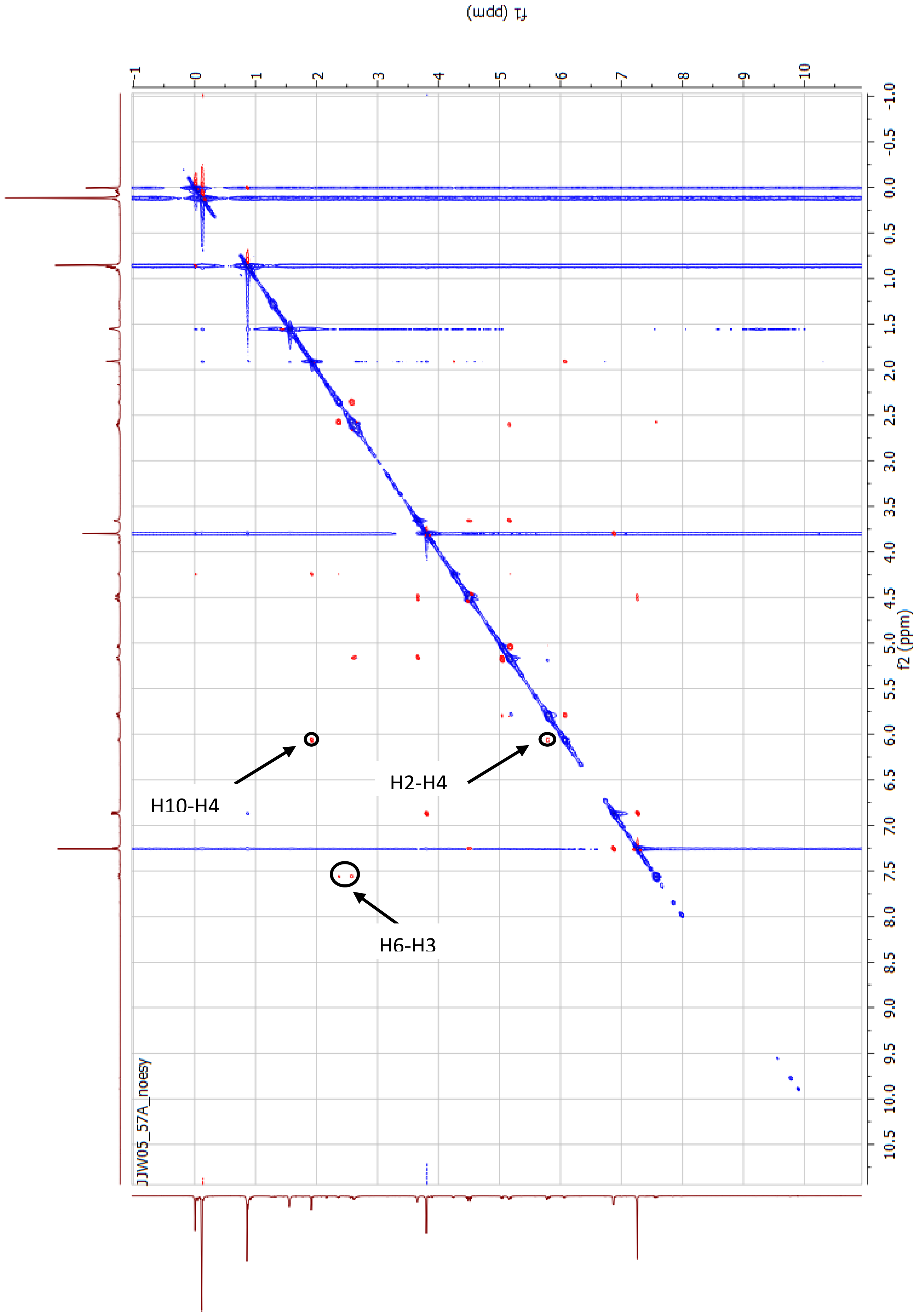
H10-H4
H2-H4
H6-H3
f1 (ppm)
f2 (ppm)
JJW05_57A_noesy

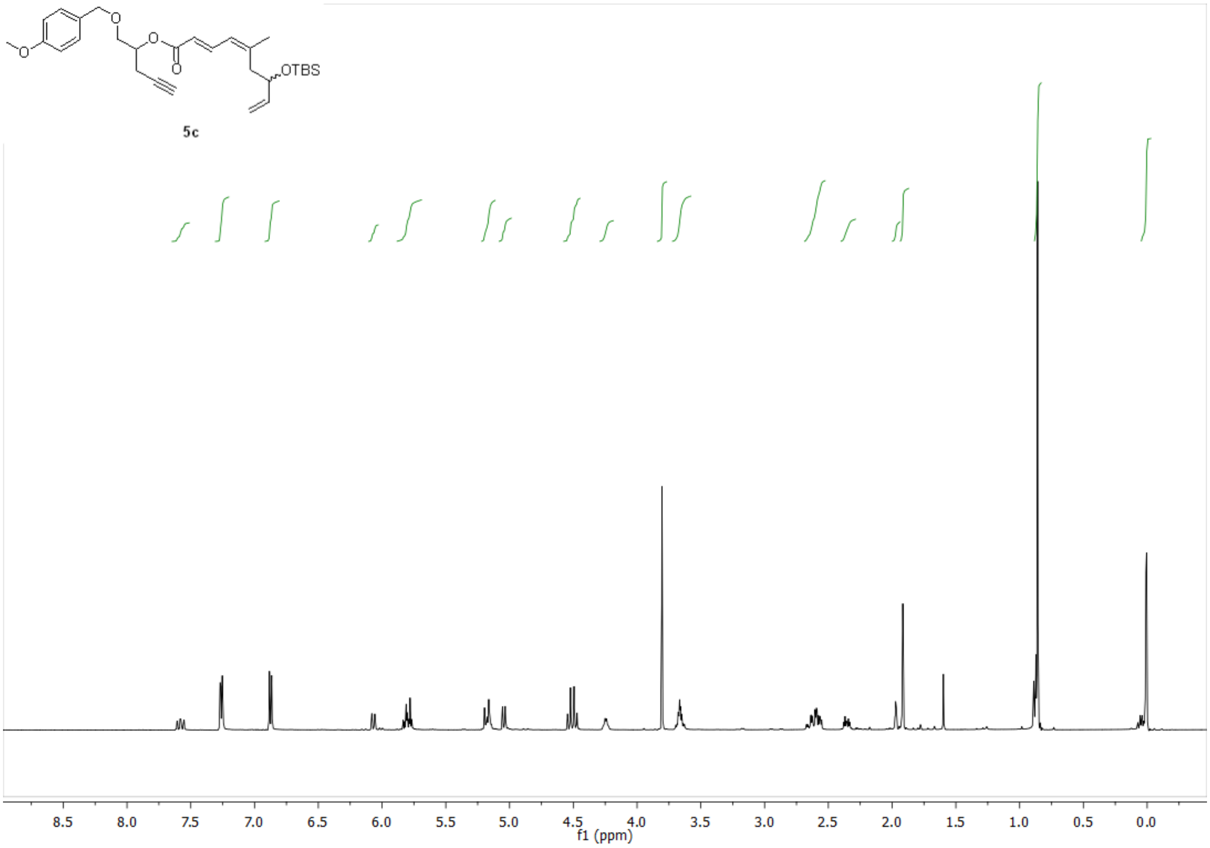
OTBS
O
O
O
O
5c
8.5 8.0 7.5 7.0 6.5 6.0 5.5 5.0 4.5 4.0 3.5 3.0 2.5 2.0 1.5 1.0 0.5 0.0
f1 (ppm)

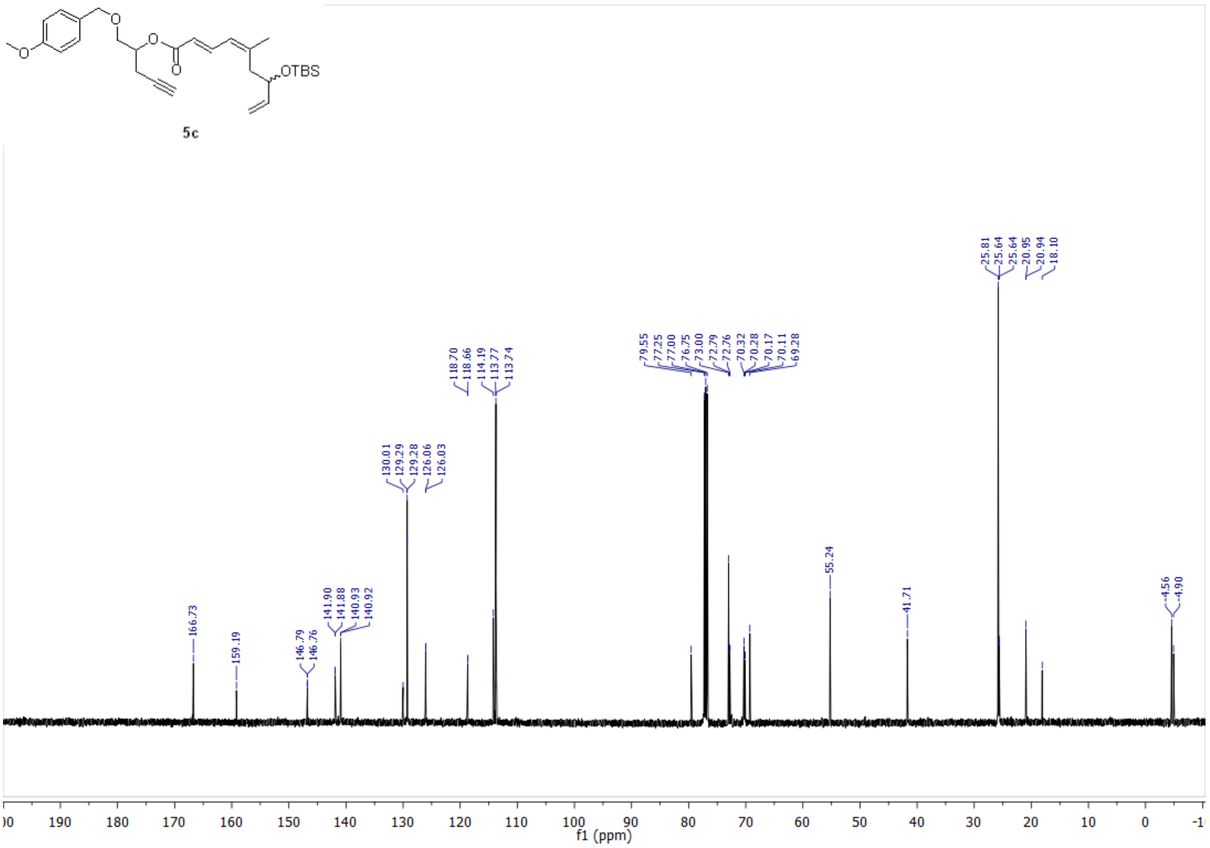
OTBS
O
O
O
O
5c
166.73
159.19
146.79
146.76
141.90
141.88
140.93
140.92
130.01
129.29
129.28
126.06
126.03
118.70
118.66
114.19
113.77
113.74
79.55
77.25
77.00
76.75
73.00
72.79
72.76
70.32
70.28
70.17
70.11
69.28
55.24
41.71
25.81
25.64
25.64
20.95
20.94
18.10
-4.56
-4.90
190 180 170 160 150 140 130 120 110 100 90 80 70 60 50 40 30 20 10 0
f1 (ppm)
